# Stage-dependent effects of systemic ASBT inhibition in a cholestasis-induced cholemic nephropathy mouse model^☆^

**DOI:** 10.1016/j.jhepr.2025.101599

**Published:** 2025-09-23

**Authors:** Ahmed Ghallab, Maiju Myllys, Daniela González, Adrian Friebel, Zaynab Hobloss, Reham Hassan, Hannah Schmidt, Qasim Siddiqui, Deng Zhipeng, Rama Hendawi, Brigitte Begher-Tibbe, Joerg Reinders, Katharina Derksen, Ute Hofmann, Julia C. Duda, Lucia Ameis, Kathrin Möllenhoff, Abdellatief Seddek, Noha Abdelmageed, Ellen Strängberg, Peter Åkerblad, Mihael Vucur, Tom Luedde, Guido Stirnimann, Matthias Schwab, Tahany Abbas, Benedikt Hild, Hartmut Schmidt, Saul J. Karpen, Benedikt Simbrunner, Mattias Mandorfer, Jörg Rahnenführer, Karolina Edlund, Stefan Hoehme, Michael Trauner, Paul A. Dawson, Erik Lindström, Jan G. Hengstler

**Affiliations:** 1 Department of Toxicology, Leibniz Research Centre for Working Environment and Human Factors, Technical University Dortmund, Ardeystr. 67, 44139, Dortmund, Germany; 2 Forensic Medicine and Toxicology Department, Faculty of Veterinary Medicine, South Valley University, Qena, Egypt; 3 Interdisciplinary Centre for Bioinformatics (IZBI) & Saxonian Incubator for Clinical Research (SIKT), University of Leipzig, Haertelstraße 16-18, 04107 Leipzig, Germany; 4 Dr. Margarete Fischer-Bosch Institute of Clinical Pharmacology and University of Tübingen, Auerbachstr. 112, 70376 Stuttgart, Germany; 5 Department of Statistics, TU Dortmund University, 44227 Dortmund, Germany; 6 Institute of Medical Statistics and Bioinformatics, University of Cologne, Germany; 7 Department of Pharmacology, Faculty of Veterinary Medicine, Sohag University, 82524 Sohag, Egypt; 8 Ipsen, Göteborg, Sweden; 9 Department of Gastroenterology, Hepatology and Infectious Diseases, University Hospital Duesseldorf, Medical Faculty at Heinrich-Heine-University, 40225 Dusseldorf, Germany; 10 University Clinic for Visceral Surgery and Medicine, Inselspital University Hospital, University of Bern, 3010 Bern, Switzerland; 11 Departments of Clinical Pharmacology, and of Biochemistry and Pharmacy, University Tuebingen, Tuebingen, Germany; 12 Histology Department, Faculty of Medicine, South Valley University, 83523 Qena, Egypt; 13 Clinic for Gastroenterology, Hepatology and Transplantation Medicine, University Hospital Essen, Essen, Germany; 14 Stravitz-Sanyal Institute for Liver Disease and Metabolic Health, Virginia Commonwealth University, Richmond, VA, United States; 15 Division of Gastroenterology and Hepatology, Department of Internal Medicine III, Medical University of Vienna, 1090 Vienna, Austria; 16 Department of Pediatrics, Division of Gastroenterology, Hepatology, and Nutrition, Emory University, Atlanta, GA 30322, United States

**Keywords:** cholestasis, acute kidney injury, bile duct ligation, entero-nephro-hepatic circulation, bile cast

## Abstract

**Background & Aims:**

Cholemic nephropathy (CN) is a severe complication of liver diseases associated with cholestasis and represents an unmet medical need. Recently, we identified the molecular mechanism of CN and showed that the systemic apical sodium-dependent bile acid transporter inhibitor (ASBTi) AS0369 prevented CN in mice. However, it is not clear if ASBTi is effective in a therapeutic rather than a preventive setting.

**Methods:**

AS0369 was administered daily for 4 weeks to bile duct-ligated (BDL) mice at four CN stages: (1) early stage with proximal tubular epithelial cell (pTEC) death (BDL-day 3); (2) inflammation, leaky peritubular capillaries, and tubular dilatation (BDL-day 21); (3) fibrosis (BDL-day 42); and (4) advanced stage with glomerular cysts (BDL-day 63). Disease progression was evaluated by biochemical, histopathological, and RNA-sequencing analysis.

**Results:**

ASBTi increased urinary excretion of bile acids (BAs) and reciprocally reduced BA concentrations in blood and renal tissue at all disease stages. Therapeutic efficacy was highest when ASBTi was given at early disease stages, *e.g*. urinary BA excretion was increased 9-fold (*p* <0.001) at the early stage compared to 4-fold (*p* = 0.021) at the late stage. ASBTi reduced the pTEC injury biomarker KIM-1, tissue damage, replacement proliferation, peritubular capillary damage and renal fibrosis. Additionally, late-stage disease features, such as glomerular cysts, were ameliorated (46% at the late stage, *p* = 0.005) by the ASBTi. RNA-sequencing revealed that ASBTi attenuated BDL-induced gene deregulation at all stages, with a larger effect size at early stages.

**Conclusions:**

Early systemic ASBTi therapy, initiated at the onset of pTEC death, provides the greatest therapeutic benefit. Nonetheless, even at later stages, ASBTi can ameliorate features of advanced CN.

**Impact and implications:**

This study demonstrates that systemic inhibition of the apical sodium-dependent bile acid transporter (ASBTi) alleviates cholemic nephropathy across disease stages in a bile duct ligation mouse model. The greatest benefit was achieved when treatment was initiated early, coinciding with proximal tubular epithelial cell death, but even advanced features such as glomerular cysts were partially reversed. These findings highlight ASBTi as a promising therapeutic strategy for cholemic nephropathy, addressing a major unmet need in cholestatic liver disease. By targeting bile acid accumulation and related injury pathways, ASBTi may improve renal outcomes and broaden treatment options in affected patients.

## Highlights

• The therapeutic effect of systemic ASBT inhibition in mice with cholemic nephropathy is disease stage dependent.
• The therapeutic effect is smaller in advanced compared to early stages but is still statistically significant.
• ASBT inhibition increases urinary excretion of bile acids and reduces bile acid levels in the kidney and blood.
• ASBT inhibition improves histological hallmarks of cholemic nephropathy, even in advanced stages.
• ASBT inhibition in normal mice has only minor effects on the liver and kidneys.

## Graphical abstract

**Figure:**
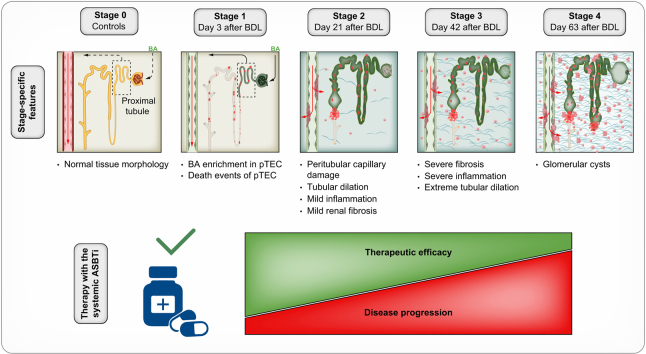

## Introduction

Cholemic nephropathy (CN) is an acute renal dysfunction, associated with high morbidity and mortality, that occurs in the context of liver diseases associated with cholestasis, including acute liver failure, alcohol-related hepatitis and decompensated cirrhosis with acute-on-chronic liver failure, as well as obstructive jaundice.[1], [2], [3], [4], [5] In preclinical studies of CN, bile duct ligated (BDL) mice are commonly used, because the animals develop several features observed in patients with CN, such as tubular epithelial cell damage, tubular casts, cystic dilatation of renal tubules, fibrosis, and compromised renal function.^1^^,^^6^^,^^7^ Recently, the spatio-temporal sequence of events leading to CN was studied in BDL mice using intravital microscopy.^7^ Already within the first hours after BDL, bile acids (BA) strongly increase in blood leading to enhanced glomerular filtration and increased BA concentrations in renal tubules. Consequently, proximal tubular epithelial cells (pTECs) enrich BA due to the activity of the a*pical* sodium-dependent bile acid transporter (ASBT; SLC10A2) leading to oxidative stress and cell death. Dying pTECs release cell debris, which floats downstream and forms obstructions in distal tubules and collecting ducts leading to dilatations upstream of the casts. Concurrently, pTECs enrich BA at the interstitial side via the activities of OSTα/β (SLC51A/SLC51B) and MRP3 (ABCC3), which leads to leaky peritubular capillaries. Next, fibrosis is triggered and – as a late event – glomerular cysts are formed.

An early key event initiating the above-described hallmarks of CN is ASBT-mediated BA enrichment in pTECs.^7^ Blocking BA enrichment in pTECs via inhibition of renal ASBT has been shown to prevent cell death events and further adverse effects of CN. Recently, a systemically bioavailable ASBT inhibitor (ASBTi) AS0369 was developed.^7^ AS0369 potently inhibits the mouse ASBT with a half-maximal inhibitory concentration (IC_50_) of 1.31 nM, shows a more than 100-fold higher affinity for mouse ASBT compared to mouse NTCP (sodium-taurocholate co-transporting polypeptide; SLC10A1), and has appropriate pharmacokinetics after oral administration so that inhibitory concentrations can be reliably maintained by two oral doses of 60 mg/kg a day.

A key limitation of the above-described study was that systemic ASBT inhibition was initiated on the same day as BDL was performed.^7^ Using this experimental design, it was demonstrated that systemic ASBT inhibition prevented CN when applied at the same time as the intervention that causes cholestasis. It is not yet known whether systemic ASBTi can also be used for therapy, and if their efficacy depends on the stage of the disease. Notably, patients with the above-mentioned conditions often present when acute kidney injury (AKI) has already occurred. Moreover, even in those without AKI at presentation, the need to apply ASBTi preventively would result in a fundamentally different approach to clinical development compared to only treating the subset of patients with evident kidney injury.

In the present study, we investigated the therapeutic efficacy of ASBTi in mice representing four increasingly advanced stages of CN. We report that while early ASBTi application in CN is essential for maximum benefit, treatment with the systemic ASBTi AS0369 yielded a clear improvement in renal histopathology and gene expression changes, even when given at the most advanced CN stage.

## Materials and methods

A detailed description of the materials and methods is provided in the supplementary data and CTAT table.

### Induction of obstructive cholestasis in mice and administration of the systemic ASBTi

Eight-to-ten-week-old male C57BL/6N mice (Janvier Labs, France) were used. The mice were housed at standard environmental conditions with free access to water, and *ad libitum* feeding on a standard rodent diet (Ssniff, Soest, Germany). All experiments were ethically approved by the local committee (LANUV, North Rhine-Westphalia, Germany, application number: 81-02.04.2022.A286). To induce obstructive cholestasis, the extrahepatic common bile duct was ligated at a position between the gallbladder and the duodenum, as previously described.^7^ Sham control mice underwent the same operative procedure but without BDL. The BDL mice received AS0369 (60 mg/kg) or vehicle (0.5% methyl cellulose and 0.1 % tween 80) orally by gavage twice per day for 4 weeks starting on either day 3, 21, 42, or 63 after the surgery.

### BA analysis

Concentrations of BA in liver and kidney tissues were determined by negative electrospray liquid chromatography-tandem mass spectrometry in MRM (multiple-reaction-monitoring) mode on an Agilent 6495B triple quadrupole mass spectrometer (Agilent, Germany) coupled to an Agilent Infinity II HPLC system. BA analysis in blood plasma and urine was accomplished by liquid chromatography-mass spectrometry (supplementary methods).

## Results

### Stage-dependent improvement of kidney injury markers with systemic ASBTi therapy

To study the stage-dependent therapeutic efficacy of systemic ASBT inhibition we chose the following disease stages after BDL: (1) day 3, when pTEC death occurs but peritubular capillaries, renal tubules, and glomeruli are intact (Fig. S1A and B); (2) day 21, when peritubular capillaries are compromised as evidenced by reduced MECA-32 immunostaining (Fig. S1A) and capillary leakage in intravital imaging (Fig. S1B) – moreover, tubular dilatation, leukocyte infiltration, but only very mild renal fibrosis are observed (Fig. S1A); (3) day 42, with severe fibrosis and extreme tubular dilatation (Fig. S1A); (4) day 63, when glomerular cysts occur, and all other above-mentioned disease characteristics become more severe (Fig. S1A).

An interesting difference in the adaptation of the liver and kidney to cholestasis was observed with respect to BA uptake transporters (Fig. S2A). Protein levels of NTCP, the major BA uptake transporter in hepatocytes, were strongly downregulated on day 3 after BDL and thereafter (Fig. S2A and B). In contrast, ASBT, the major BA uptake transporter in renal pTECs was unaffected or only moderately downregulated (Fig. S2A and C). Thus, the liver and kidney show marked differences in their ability to adapt to cholestasis.

**Fig. 1:**
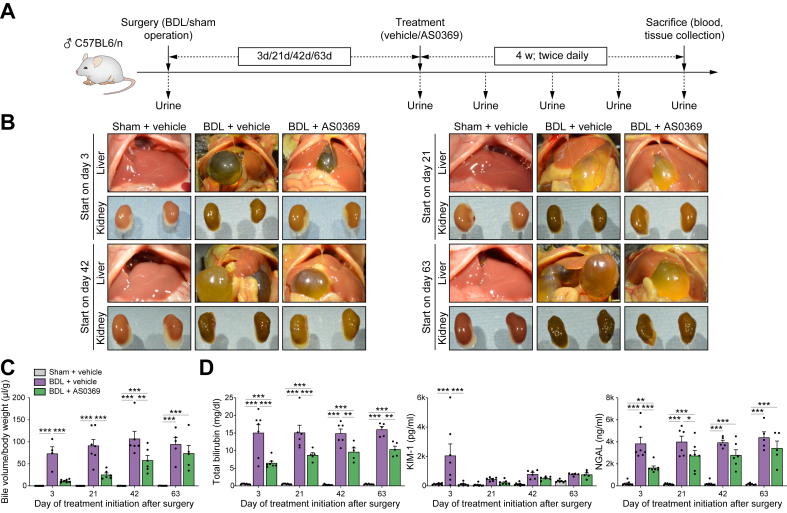
**Therapeutic efficacy of AS0369 in four disease stages.** (A) Experimental design; 60 mg/kg AS0369 or vehicle were given twice daily by gavage for 4 weeks. (B) Macroscopic appearance of the livers and kidneys; (C) Ratio of bile volume to body weight; (D) Concentrations of total bilirubin, KIM-1 and NGAL in the blood. ∗*p* <0.05; ∗∗*p* <0.01; ∗∗∗*p* <0.001; Tukey's multiple comparisons test. Data are presented as mean ± SEM. n = 4-9 mice per group; the dots in the bars represent individual mice. BDL: bile duct ligation; KIM-1: kidney injury molecule1; NGAL: neutrophil gelatinase-associated lipocalin.

To study the influence of the stage of CN on therapeutic efficacy, we used a 4-week treatment period with the systemic ASBTi AS0369, starting 3 (stage 1), 21 (stage 2), 42 (stage 3), or 63 (stage 4) days after BDL (Fig. 1A). BDL caused a macroscopically visible greenish discoloration of the kidneys, which was ameliorated by AS0369 therapy in all disease stages (Fig. 1B). BDL massively increased the size of the gallbladder and bile volume, which was reduced by AS0369 (Fig. 1B,C). This macroscopic observation was evident when quantified as the bile volume to body weight ratio, and the AS0369 effect size was more pronounced when initiated at the earlier stages (Fig. 1B,C). Total bilirubin concentrations in the blood were significantly decreased by the AS0369 therapy at all disease stages (Fig. 1D). KIM-1, a biomarker of pTEC injury, is known to increase only in the first days after BDL.^7^ Consistent with previous results, KIM-1 strongly increased in blood at the early stage after BDL, followed by lower levels in the later stages (Fig. 1D). This early (day 3) increase was completely suppressed by AS0369 (Fig. 1D). Neutrophil gelatinase-associated lipocalin (NGAL) is an injury marker of all tubular epithelial cells and increased in blood to a similar extent at all periods after BDL (Fig. 1D). AS0369 significantly reduced NGAL levels when therapy started on days 3 and 21 but the reduction in NGAL levels did not reach statistical significance at the later stages (Fig. 1D). BDL caused a loss of body weight that was ameliorated by AS0369 if initiated up to stage 3, but not at stage 4, where no significant effect was obtained (Fig. S3A). Approximately 20–30% of BDL mice either died or exceeded our health score criteria, requiring euthanasia. Treatment with AS0369 improved survival (defined by death or exceedance of the score sheet criteria) for the groups treated at stages 1 to 3 after BDL (Fig. S3B). If therapy was initiated at stage 4, the percentage of surviving mice during the 4-week treatment period was higher, which may reflect adaptation to the cholestatic situation for mice that have survived the longer BDL periods (*i.e.* survivorship bias).

### Target protection and reduced systemic BA load by systemic ASBT inhibition in all disease stages

**Fig. 2:**
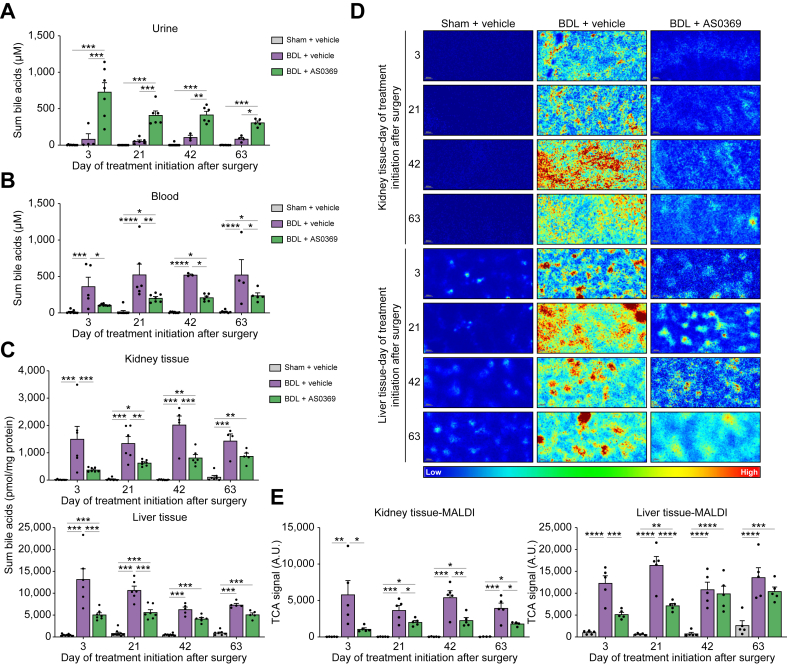
Influence of AS0369 on BA concentrations. (A-C) Sum BA in urine, blood plasma, and kidney as well as liver tissue homogenates. (D) Tissue TCA levels analyzed by MALDI-MSI in frozen kidney and liver tissues. (E) Quantifications of TCA MALDI-MSI signals in kidney and the liver tissues. ∗*p* <0.05; ∗∗*p* <0.01; ∗∗∗*p* <0.001; ∗∗∗∗*p* <0.0001; Tukey's multiple comparisons test. Data are presented as mean ± SEM. n = 3-9 mice per group; the dots in the bars represent individual mice. BA, bile acid; BDL: bile duct ligation; MALDI-MSI, matrix assisted laser desorption/ionization mass spectrometry imaging; TCA, taurocholic acid; TCMA, tauro-muricholic acid.

Therapeutic effects of the systemic ASBTi can be achieved via two mechanisms: by reduced uptake of BA into the target cells (pTECs) of the kidney (target protection), and by reduced systemic BA load because of increased urinary BA excretion (systemic effect). AS0369 caused a marked increase in urinary BAs and a corresponding decrease in blood BA at all four disease stages, with a smaller effect when therapy was initiated later after BDL (Fig. 2A,B). Total BAs detected in kidney tissue homogenate were significantly reduced by AS0369 when treatment began up to stage 3, indicating efficient target protection (Fig. 2C). The results obtained by mass spectrometry of tissue homogenate were confirmed by matrix assisted laser desorption/ionization mass spectrometry imaging of tissue sections, demonstrating that AS0369 strongly reduced the TCA signal in the kidney tissue of BDL mice at all disease stages (Figs 2D,E and S4). Also in liver tissue, AS0369 significantly reduced levels of BAs in BDL mice in the two earliest (days 3 and 21) but not the later (days 42 and 63) disease stages (Figs 2C-E and S5). The changes in BA concentrations in kidney tissues following AS0369 treatment were associated with changes in BA transporter expression (Fig. S6). AS0369 ameliorated the influence of BDL on *Asbt, Oat3, Mrp3* and *Mrp4* RNA levels in the kidney at early disease stages, with the effect size decreasing in later stages (Fig. S6). No significant alterations in *Ost-α* and *Mrp2* expression were observed in the kidney tissues following AS0369 treatment (Fig. S6). In contrast to the kidney, BDL-induced alterations of the hepatic BA transporters remained mainly unaffected by the ASBTi (Fig. S7).

### Stage-dependent improvement of renal tissue injury by systemic ASBT inhibition

**Fig. 3:**
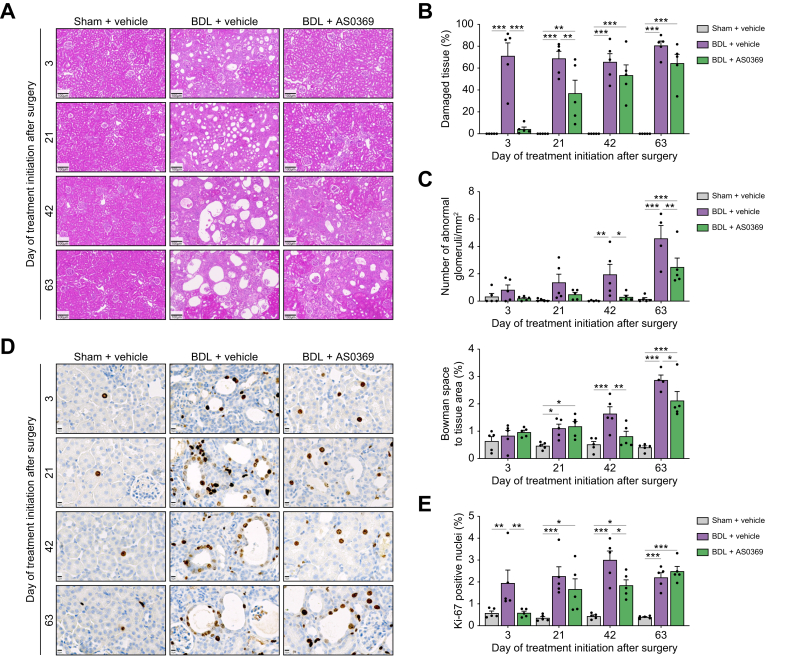
Renal tissue damage and replacement proliferation after obstructive cholestasis and therapy by AS0369. (A) H&E-stained renal tissue sections; scale bars: 100 μm. (B) Quantification of damaged tissue; (C) Number of abnormal glomeruli per area and overall area of Bowman space to the total tissue area; (D) Proliferating cells (brown nuclei) based on ki67 staining; scale bars: 10 μm; (E) Percentage of Ki-67 positive nuclei. ∗*p* <0.05; ∗∗*p* <0.01; ∗∗∗*p* <0.001; Tukey's multiple comparisons test. Data are presented as mean ± SEM. n = 4-5 mice per group; the dots in the bars represent individual mice. BDL: bile duct ligation.

A relatively early feature of CN is the occurrence of damaged tissue and dilated renal tubules, which occur 3-4 weeks after BDL.^7^ H&E-stained kidney tissue revealed that AS0369 therapy ameliorates renal damage, including tubular dilation, across all disease stages (Fig. 3A). To quantify the extent of damaged renal tissue in whole-organ sections, the intensity of the eosin staining can be used,^7^ since features of tissue damage, such as dilated tubules, fibrosis, and peritubular capillary damage occur predominantly in the paler regions (Fig. S8). Automated whole-organ analysis revealed a numerical reduction in damaged tissue across all disease stages following AS0369 treatment, but statistical significance was only achieved in the two earliest stages (Fig. 3A, B). Glomerular cyst formation, a hallmark of advanced CN^7^ was assessed by quantifying the areas of glomeruli and Bowman’s space in whole-organ scans. This analysis revealed that this advanced CN feature could be significantly ameliorated even when therapy started at stages 3 and 4 (Fig. 3A,C). This finding was further supported by automated analysis of Bowman’s space on whole-organ sections, which showed increased Bowman’s space area at stage 3 and 4 post-BDL. Notably, AS0369 therapy significantly mitigated this pathological expansion (Fig. 3C).

**Fig. 4:**
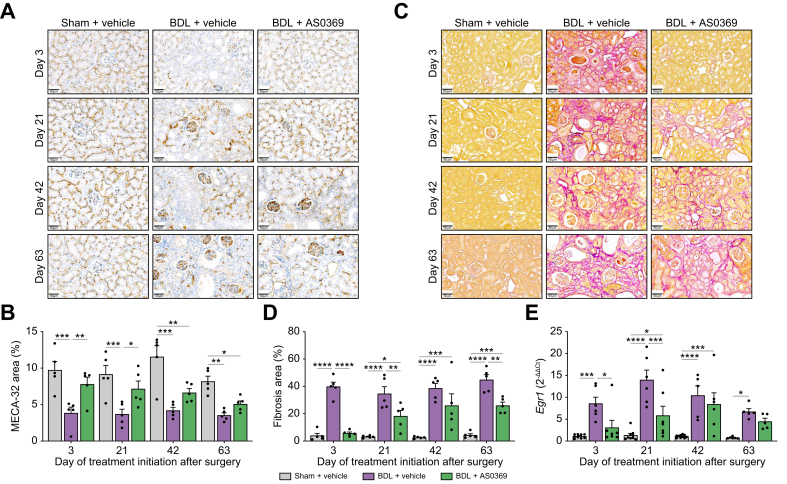
Damage of peritubular capillaries and renal fibrosis after obstructive cholestasis and rescue by AS0369 treatment. (A, B) Immunostaining of the endothelial cell marker MECA-32 and corresponding quantifications; scale bars: 50 μm. (C, D) Sirius red staining and corresponding quantifications; scale bars: 50 μm. (E) RNA expression of *Egr1* in kidney tissues. ∗*p* <0.05; ∗∗*p* <0.01; ∗∗∗*p* <0.001; ∗∗∗∗*p* <0.0001; Tukey's multiple comparisons test. Data are presented as mean ± SEM. n = 5-7 mice per group; the dots in the bars represent individual mice. BDL: bile duct ligation.

A typical response to tissue injury is replacement proliferation to regenerate the lost tissue. Following BDL, tubular epithelial cells begin to proliferate because of cell death (Fig. 3D). AS0369 treatment significantly reduced proliferation when initiated on stage 1 or 3, while no signification reduction was observed for stage 2 or 4 (Fig. 3E). Compromised capillary integrity, a further feature of BDL-induced damage (Fig. 4A), was also improved by AS0369 when therapy began on stage 1 or 2 post-BDL (Fig. 4B); however, statistical significance was not achieved for later stages. Renal fibrosis, a key feature of CN, was strongly reduced by AS0369 treatment at all disease stages except for stage 3 where statistical significance was not reached (Fig. 4C, D). Additionally, *Egr1* RNA, a well-established marker of tissue inflammation and fibrogenesis,^7^ was significantly reduced by AS0369 therapy at stages 1 and 2 but not at the later stages (Fig. 4E).

### Smaller therapeutic effect size on the liver compared to kidney after systemic ASBT inhibition in obstructive cholestasis

**Fig. 5:**
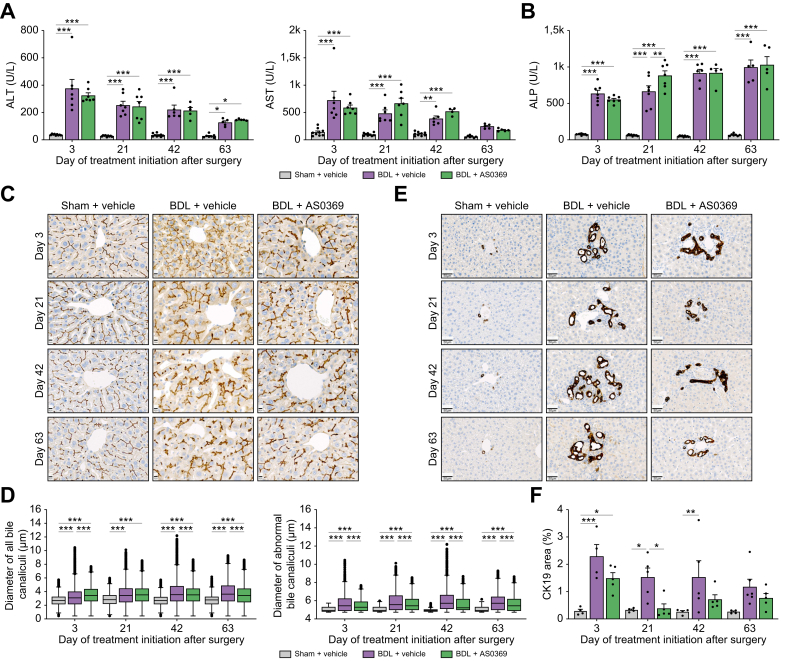
Characterization of liver damage after obstructive cholestasis and influence of AS0369 treatment. (A, B) Transaminases and alkaline phosphatase activities measured in plasma; n = 4-10 mice per group; the dots in the bars represent individual mice. (C) Visualization of bile canaliculi by immunostaining against CD13; scale bars: 10 μm; (D) Quantification of bile canalicular diameter; n = 5 mice per group. (E) Visualization of bile ducts by immunostaining against CK19; scale bars: 50 μm; (F) Percentage of CK19-positive area to the total tissue area; n = 4-5 mice per group; the dots in the bars represent individual mice. Data are presented as mean ± SEM. ∗*p* <0.05; ∗∗*p* <0.01; ∗∗∗*p* <0.001; Tukey's multiple comparisons test. BDL: bile duct ligation; ALT: alanine transaminase; AST: aspartate transaminase; ALP: alkaline phosphatase.

Next, we analyzed the livers of the same mice whose kidneys have already been described above, to directly compare the therapeutic efficacy of systemic ASBT inhibition in both organs. No significant reduction in the activity of transaminases was achieved with AS0369 (Fig. 5A,B). BDL caused a dilation of bile canaliculi that appeared to be ameliorated by AS0369 in tissue sections immunostained against CD13 (Fig. 5C). To quantify this effect, the bile canalicular diameter was determined in whole slide scans to avoid any selection bias. When all bile canalicular diameters were included into the analysis, an increase in the mean canalicular diameter due to BDL was seen, but the effect size of AS0369 therapy was extremely small (Fig. 5D). However, when only bile canaliculi with an abnormally wide diameter were included in the analysis (defined as bile canaliculi with diameter exceeding Q3+1.5 × [Q3−Q1], where Q3 and Q1 represent the upper and lower quartiles of the sham control distribution), a larger AS0369 treatment-associated reduction in canaliculi diameter was readily observed (Fig. 5D, right panel). It should be noted that when interpreting the results of the bile canaliculi analysis, diameters were measured for every pixel of the medial lines, to account for diameter variability along individual branches, leading to very high numbers of analyzed structures. To avoid test overfitting due to large numbers, we performed stratified statistical sampling using a fixed number of measurements per stratum (Fig. S9). Here, strata refer to the experimental groups formed by each unique combination of treatment and timestamp. Initially, we selected a large subset comprising 1,000,000 measurements per group, subsequently reducing the subset sizes by one-tenth increments, down to 1,000 measurements, and examined their statistical features (mean and standard deviation) compared to the base line of the original groups. The analysis showed that the statistical features remained consistent across subsets down to sample sizes of 10,000 measurements, with maximum changes of 0.49% in the mean and 10.67% in the standard deviation compared to the original sample. In contrast, for subsets of 1,000 measurements maximal changes were 2.19% in the mean and 10.60% in the standard deviation (Fig. S9).

Quantification of the cholangiocyte marker CK19-positive area on whole slide scans revealed a numerical reduction with AS0369 therapy in all disease stages, but statistical significance was only obtained for day 21 (Fig. 5E,F). Analysis of the Sirius red-positive fibrotic area showed a reduction with AS0369 therapy, particularly in the perisinusoidal region, with a relatively small effect size for all disease stages, but statistical significance was only reached for day 42 (Fig. S10).

### Amelioration of BDL-induced gene expression changes by systemic ASBT inhibition

**Fig. 6:**
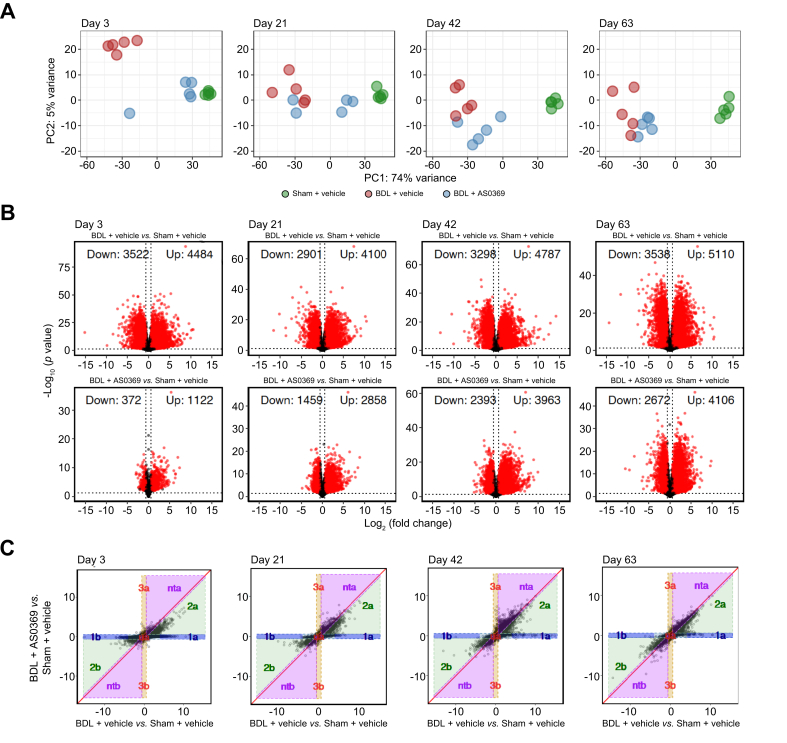
RNA-sequencing analysis of renal tissue after obstructive cholestasis with and without ASBT inhibition. (A) Principal component analysis; each dot represents an individual mouse. (B) Volcano plots illustrate differentially expressed genes; red dots: significantly deregulated genes; black dots: not significantly altered genes; vertical dotted lines: threshold values log_2_(1.5) and -log_2_(1.5); horizontal dotted line: threshold value 0.05 transformed via -log_10_(0.05). (C) DiPa-plots illustrating the therapeutic efficacy of AS0369. The nta area contains “not treatable” genes. n = 5 mice per group. BDL: bile duct ligation; DiPa, differentiation pattern.

RNA-sequencing (RNA-seq) analysis of the above-described kidney tissue specimens (Fig. 1A) was performed using genome-wide expression changes as an unbiased measure of the efficacy of therapy. Sham-operated controls and vehicle-treated BDL mice clustered in distinct regions of the principal component analysis (Fig. 6A). Treatment of BDL mice with AS0369 led to a shift almost completely back to controls when the therapy began at stage 1 (3 days after BDL). The extent of this shift decreased when therapy began later. Number, fold-change, and *p* values of the individual genes were visualized by volcano plots, illustrating the stage dependence of the ASBT-inhibiting therapy (Fig. 6B). For example, the number of downregulated genes in BDL mice in the day 3-cohort (stage 1) was 3,522 (BDL and vehicle), compared to only 372 downregulated genes in BDL mice who were treated with AS0369. The corresponding numbers were 3,538 (BDL and vehicle) and 2,672 (BDL and AS0369) when AS0369 therapy was initiated at stage 4 (63 days after BDL).

The efficacy of systemic ASBT inhibition was visualized by contrasting the ratio of BDL (vehicle) *vs.* sham (vehicle) against the ratio of BDL (AS0369) *vs.* sham (vehicle), with each dot representing an individual gene in the resulting “differentiation pattern (DiPa) plot” (Fig. 6C). If the treatment with AS0369 did not affect gene expression, the individual genes would cluster on and around the diagonal. In contrast, an effect on gene expression is represented by expression pattern groups 1a and 1b, including genes that are up- (or down-) regulated by BDL but brought back to their normal expression range by AS0369 therapy. Correspondingly, expression pattern groups 2a and 2b represent a partial success regarding AS0369 counteracting gene expression changes induced by BDL. On the contrary, for genes in the purple region (nta = not treatable) AS0369 therapy would even enhance the effect of BDL. It is interesting to note the relatively high number of genes in expression pattern groups 2a and 2b when therapy was initiated at stage 1 after BDL, which decreased when treatment was initiated in more advanced disease stages. Correspondingly, the number of genes on and around the diagonal increased the later ASBT inhibition was initiated. Expression pattern groups 3a and 3b represent genes that were not influenced by BDL but were up- or downregulated by AS0369.

Since the efficacy of AS0369 therapy decreased when initiated at stage 4 compared to stage 1, we next focused specifically on the set of initially (stage 1) responsive genes (Fig. S11A; Table S3). Genes falling into expression pattern group 1a upon treatment initiation at stage 1 (n = 2,342) were, when therapy was initiated stage 4, primarily found in the nta expression pattern group (n = 1657), while 470 genes “migrated” to expression pattern group 2a and 215 genes remained in 1a; a similar pattern was observed for genes initially located in expression pattern group 1b (Fig. S11A). This highlights the stage-dependent effect of the treatment: with delayed initiation, genes that were fully rescued at stage 1 (1a) were largely unaffected at stage 4 (nta), some were only partially rescued (2a), and only a small fraction remained fully rescued (1a). Conversely, most genes initially in expression pattern groups nta and ntb remained in these groups upon later treatment initiation. Interestingly, genes found in the subsets of expression pattern groups 1a and 1b which are here denoted as “extreme regions” (1a extreme or 1b extreme), for which BDL caused a particularly strong (at least 17-fold) up- or downregulation, were mostly responsive and primarily migrated to expression pattern group 2a and 2b (Fig. S11A). This demonstrates that the expression of genes most strongly altered due to BDL at stage 1, were still modified with partial success by AS0369 treatment initiated at stage 4 after BDL.

Finally, to gain functional insights, we analyzed Gene Ontology groups influenced by BDL as well as BDL followed by AS0369 therapy. Genes upregulated in response to BDL were mostly inflammation-associated, such as ‘inflammatory response’, ‘interleukin-6’, ‘neutrophil chemotaxis’, ‘ERK1, ERK2’, and ‘lipopolysaccharide’ (Fig. S11B). These motives were similar in all disease stages. Among downregulated genes, mostly metabolism-associated functions were enriched, such as beta-oxidation or cholesterol biosynthesis. Upregulated genes that were 'treatable' (1a and 2a) at different time points showed an enrichment of inflammation-associated motives (Fig. S12); downregulated genes (1b and 2b) that were not treatable were enriched with metabolism-associated motives. This suggests that treatable and not treatable genes do not show major differences with respect to their Gene Ontology groups.

**Fig. 7:**
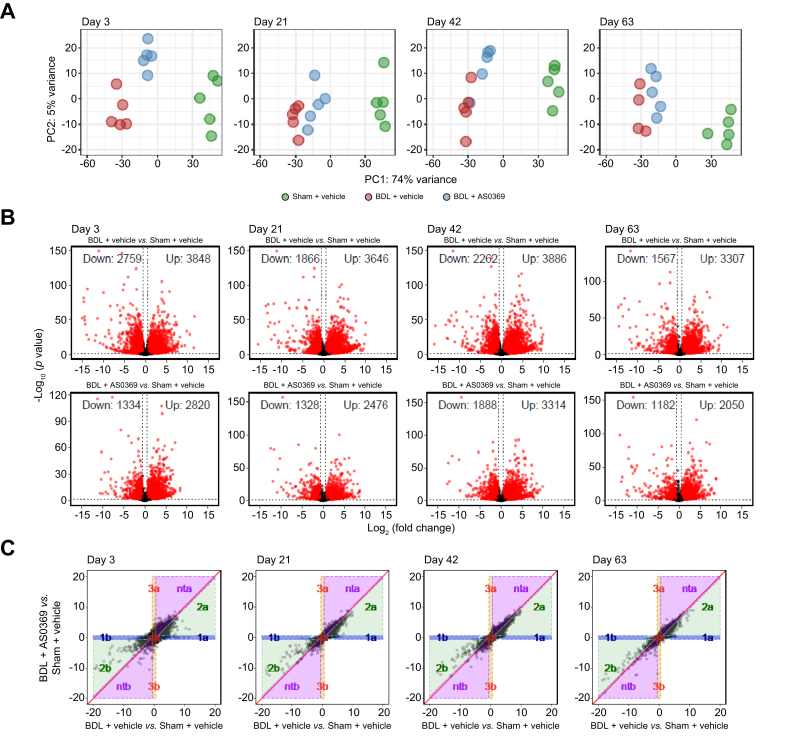
RNA-sequencing analysis of liver tissue after obstructive cholestasis with and without ASBT inhibition. (A) Principal component analysis; each dot represents an individual mouse. (B) Volcano plots illustrate differentially expressed genes; red dots: significantly deregulated genes; black dots: not significantly altered genes; vertical dotted lines: threshold values log_2_(1.5) and -log_2_(1.5); horizontal dotted line: threshold value 0.05 transformed via -log_10_(0.05). (C) DiPa-plots illustrating the therapeutic efficacy of AS0369. n = 5 mice per group. BDL: bile duct ligation; DiPa, differentiation pattern.

A similar RNA-seq analysis as for the kidney was also performed in liver tissue of the same mice (Figs 7, S13, and S14; Table S4). An important difference compared to kidney was the much smaller effect size of AS0369 therapy for the liver. Principal component analysis of the liver highlights the strong effect of BDL on global gene expression and the only moderate effect of AS0369, which decreased from the stage 1 to stage 4 treatment groups (Fig. 7A). This corresponds to the results of the volcano plots, where 2,759 genes were downregulated by BDL in the stage 1 cohort (vehicle control) compared to only 1,334 downregulated genes with AS0369 therapy (Fig. 7B). Up to stage 4 these numbers decreased to 1,567 (BDL, vehicle) and 1,182 (BDL, AS0369 therapy), respectively (Fig. 7B). The decrease in deregulated genes when treatment is initiated after longer periods after BDL is probably explained by hepatic adaptation to the cholestatic situation.^8^ The smaller effect size of AS0369 therapy in the liver compared to the kidney is also illustrated by the relatively small fraction of genes in expression pattern groups 1a and 1b of the DiPa-plots (Figs 7C and S13). Gene Ontology analysis showed an enrichment of inflammation-associated motifs among upregulated genes and metabolism-associated motifs among downregulated genes (Fig. S13 and 14).

### Systemic ASBT inhibition in healthy mice causes no morphological or functional alterations and only minor molecular changes in the liver and kidneys

**Fig. 8:**
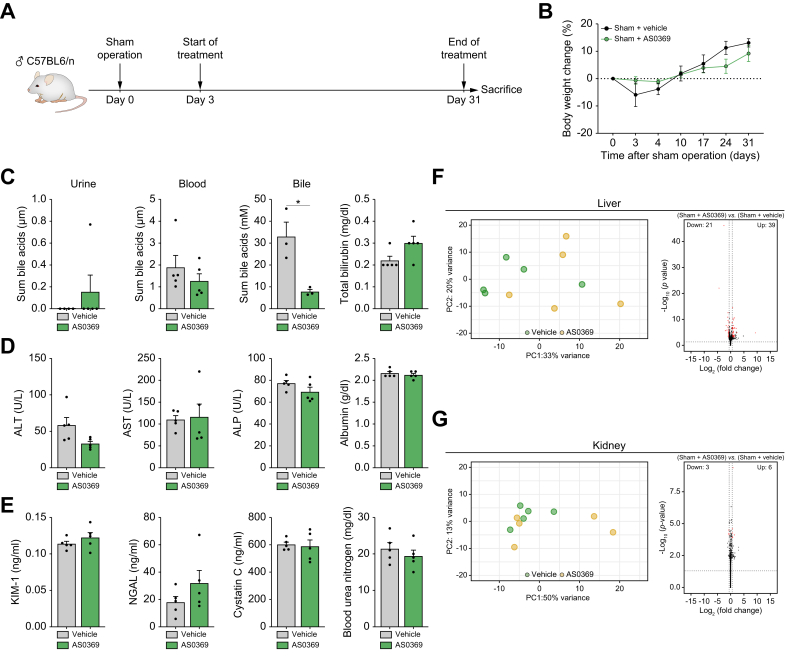
Systemic ASBT inhibition has only minor effects in the liver and kidneys of healthy mice. (A) Experimental design. (B) Body weight changes. (C) Sum bile acid concentrations in blood, urine and bile (∗*p* = 0.0206; Unpaired *t* test), and total bilirubin levels in blood. (D) Blood biomarkers of liver injury or function. (E) Biomarkers of kidney injury in urine (KIM-1, NGAL) and kidney functions in blood (cystatin C, blood urea nitrogen). Data are presented as mean± SEM. n = 3-5 mice per group; the dots in the bars represent individual mice. (F, G) RNA-sequencing analysis of liver (F) and kidney (G) tissues including principal component analysis (left panels) and volcano plots illustrating differentially expressed genes (right panels); red dots: significantly deregulated genes; black dots: not significantly altered genes; vertical dotted lines: threshold values log_2_(1.5) and -log_2_(1.5); horizontal dotted line: threshold value 0.05 transformed via -log_10_(0.05); n = 5 mice per group. ALT: alanine transaminase; AST: aspartate transaminase; ALP: alkaline phosphatase; KIM-1: kidney injury molecule1; NGAL: neutrophil gelatinase-associated lipocalin.

To assess the influence of ASBTi in non-cholestatic controls, mice on day 3 after sham operation were repeatedly treated with AS0369 or vehicle twice daily for 4 weeks (Fig. 8A). Analysis of body weight changes revealed no difference in the ASBTi-treated group compared to the vehicle controls (Fig. 8B). No significant differences in BA concentrations in blood and urine were observed in response to ASBTi treatment; in contrast, ASBTi caused significant reduction of BA concentrations in bile (Fig. 8C). Analysis of total bilirubin, liver enzymes, albumin, cystatin C, blood urea nitrogen, and kidney injury biomarkers (KIM-1, NGAL) in blood revealed no significant changes between ASBTi- and vehicle-treated mice (Fig. 8C-E). In agreement, histological analysis of the liver and kidneys revealed no alterations (Fig. S15). Thus, ASBTi treatment in healthy mice causes no morphological/functional adverse effects.

To investigate possible molecular changes, RNA-seq analysis was performed in the liver and kidney tissues. In the liver, a few moderate gene expression changes were observed (Fig. 8F; Table S5A). As expected, Cyp7a1 was among the upregulated genes, since ASBTi decreases BA reabsorption from the intestine and the upregulation of genes responsible for BA synthesis in hepatocytes represents an adaptive response. Nevertheless, the extent of global expression changes in the liver in response to AS0369 is small. In the kidney, only very minor gene expression changes were observed after ASBTi (Fig. 8G; Table S5B).

## Discussion

CN is a severe, often lethal complication of liver disease associated with cholestasis and represents an unmet therapeutic need. To study the therapeutic efficacy of the systemically bioavailable ASBT inhibitor AS0369, we used an animal model of obstructive cholestasis where four distinct stages ranging from an early to a severe stage of CN can be reproducibly generated. Mice in stages 1 to 4 were treated for 4 weeks with two oral doses of AS0369 per day. For interpretation of the data, it should be considered that the time periods of the four disease stages (days 1, 21, 42 and 63 after BDL) indicate the beginning of the 4-week treatment period with AS0369 or vehicle. Data after 4 weeks of AS0369 therapy were then compared to the corresponding vehicle controls.

The results of this study demonstrate that systemic ASBT inhibition caused specific improvements in all stages, but the effect on some disease features was stage dependent. In all stages, AS0369 therapy caused an increase of total BA concentrations in urine and a reciprocal BA reduction in the blood, as well as in renal and liver tissues. Also, total bilirubin concentrations decreased in all disease stages. In contrast, the levels of the pTEC injury marker KIM-1 decreased only in stage 1, where AS0369 reduced KIM-1 almost to control levels. This stage dependency is probably explained by the fact that the death events for the pTECs are mostly observed early after BDL. A strong reduction of kidney tissue damage was seen in stage 1, while the effect size of AS0369 therapy decreased for stage 2 and was no longer significant for stages 3 and 4. A similar tendency was observed for the replacement proliferation of TECs and amelioration of tubular dilatation. In addition, the therapeutic effect on fibrogenesis was much stronger in early compared to late stages. Nevertheless, AS0369 therapy also ameliorated some specific features of stages 3 and 4. The size of glomeruli (glomerular cysts) begins to increase at stage 3 and progresses to extremely large glomeruli with dilated Bowman’s spaces in stage 4. In both stage 3 and 4, AS0369 treatment significantly reduced glomerular cysts. The results demonstrate that the onset of therapy with systemic ASBT inhibitors should be early, ideally when cell death events are beginning for pTECs. This aspect is critical for evaluating potential indications and clinical development, as the management of AKI in patients with liver disease largely relies on serum creatinine as an indicator of kidney dysfunction, which rises after damage has occurred.^9^ Besides monitoring the pTEC injury marker KIM-1 which may be an early marker of CN in patients with cholestasis,^7^ monitoring serum and/or urinary NGAL is a promising strategy for detecting tubular damage (*i.e.* subclinical AKI) that preceded the functional impairment that defines AKI in the clinic. This concept (damage *vs*. function) has been emphasized in the recent ADQI (Acute Disease Quality Initiative) and ICA (International Club of Ascites) joint multidisciplinary consensus meeting.^4^ Moreover, urinary levels of the latter biomarker have been shown to differentiate between prerenal AKI, hepatorenal syndrome, and acute tubular necrosis, *i.e*. AKI subtypes in patients with cirrhosis that are characterized by increasing severity of tubular injury and decreasing probability of functional recovery.[10], [11], [12] Thus, even in patients with clinically evident AKI/kidney dysfunction, low levels of damage markers may identify those with prerenal AKI, an exclusively functional and reversible form of kidney dysfunction due to volume depletion. Notably, in patients with acute tubular necrosis, urinary NGAL remained persistently high,^13^ which resembles observations in animals with CN, in which ASBTi lowered NGAL levels, particularly if initiated within the first two stages of CN. In line, ASBTi decreased KIM-1, which is also increased in human AKI in the context of liver disease.^14^ However, systemic ASBTi may still be therapeutically beneficial in advanced stages, since systemic and tissue levels of BAs can also be reduced in stages 3 and 4, which possibly explains the amelioration of stage-specific disease features such as glomerular cysts. Since the current study summarizes data from a 4-week treatment period with AS0369, it cannot be excluded that longer treatment periods would ameliorate advanced CN further. From a clinical perspective, ASBTi treatment, if effective in human disease, could serve as a bridging therapy until the recovery of liver function/cholestasis in those with acute or acute-on-chronic liver injury/failure, or liver transplantation.

Interestingly, a striking difference was observed in the therapeutic efficacy of systemic ASBTi in the kidney *vs*. the liver in this model of obstructive cholestasis. While the therapeutic effect of AS0369 therapy on the kidney was large, only a comparatively small effect size was observed in the liver. A consistent, statistically significant influence of AS0369 in the liver in all four disease stages was the amelioration of bile canalicular dilation caused by BDL. It is known that cholestasis leads to the dilation of bile canaliculi,^8^ and the improved canalicular diameter may be a particularly sensitive marker that is responsive to the reduced total BA concentrations in the blood and liver tissue. It should be considered that hepatocytes adapt to BDL-induced cholestasis by strongly downregulating NTCP and inducing expression of sinusoidal membrane BA exporters in an attempt to reduce the hepatocyte BA burden. However, hepatocyte BA synthesis is ongoing, and plasma BAs continue to be taken up via non-NTCP BA carriers and any residual NTCP activity. In contrast to hepatocytes, renal pTECs do not show a similarly efficient adaptation since ASBT remained expressed and functional in all disease stages. Thus, protection against intracellular BA enrichment in the pTECs can be achieved solely by inhibiting ASBT. Although AS0369 increased urinary BA excretion and reduced plasma BA levels, the treatment does not block ongoing hepatocyte BA synthesis, and the new steady-state hepatic BA burden remains above the threshold for liver injury in this model of severe obstructive cholestasis. These differences in target protection achieved in the kidney but not in the liver may explain the different therapeutic efficacy of ASBTi in both organs.

Analysis of renal genome-wide expression profiles by RNA-seq after BDL demonstrated a remarkable therapeutic effect size of AS0369. In the earliest disease stage (day 3 after BDL, stage 1), AS0369 therapy rescued 89.4% of all genes downregulated in response to BDL and 74.9% of all upregulated genes. This percentage decreased when therapy began at later stages, but the number of rescued genes was still 24.5% (down) and 19.6% (up) in the most advanced stage (stage 4). Plotting the effects of BDL with vehicle *vs*. BDL with ASBT inhibition resulted in three expression pattern groups, where genes up- or downregulated in response to BDL are either completely, partially or not rescued by the therapy. A fraction of the genes completely rescued in stage 1 moved to the partially or not rescued category when the therapy began at later disease stages. RNA-seq analysis of the livers of the same mice demonstrates a smaller therapeutic effect compared to the kidney. However, in disease stage 1, AS0369 therapy rescued 51.6% of downregulated genes and 26.7% of upregulated genes in liver tissue, illustrating that the effect of the ASBTi could still be quantified.

Although systemic ASBTi efficiently enhances urinary BA excretion and reduces BA blood concentrations, it should be considered that the blood BA concentrations of BDL mice were not reduced to normal levels of less than 2 μM (in mice) after AS0369 treatment but remained between 100 and 200 μM. As noted above, this is likely explained by continued synthesis of BA in the cholestatic liver as evidenced by high levels of C4 under AS0369 exposure.^7^ Future studies should address if blood BA concentrations in cholestasis can be decreased even further by combining systemic ASBTi^7^^,^^15^ with additional drugs, such as synthetic FXR agonists, FGF15/19 mimetics or PPAR agonists to inhibit BA synthesis,[16], [17], [18], [19], [20] NTCP inhibitors to block hepatic BA uptake, or with therapeutic BAs to reduce lipophilicity of the BA pool.[21], [22], [23]

In conclusion, systemic inhibition of ASBT is an efficient therapeutic option for CN in mice. Our study demonstrated that therapy should ideally begin as soon as pTEC damage occurs to maximize treatment benefit, but ASBTi still showed an effect at late stages of CN. These observations are instrumental for identifying potential indications in humans as well as informing clinical drug development.

## Abbreviations

ASBT, apical sodium-dependent bile acid transporter; BAs, bile acids; BDL, bile duct ligation; CN, cholemic nephropathy; DiPa, differentiation pattern; Egr1, early growth response protein 1; KIM-1, kidney injury molecule 1; MRP, multidrug resistance-associated protein; NGAL, neutrophil gelatinase-associated lipocalin; NTCP, Na+-taurocholate co-transporting polypeptide; OAT3, organic anion transporter3; OST-α/β, Organic Solute Transporter-α/β; pTECs, proximal tubular epithelial cells.

## Financial support

A.G. was funded by the German Research Foundation (DFG; Project IDs 517010379& 457840828). UH and MS were funded by the Robert Bosch Stiftung, Stuttgart, Germany. LA was funded by the DFG (the Research Training Group “Biostatistical Methods for High-Dimensional Data in Toxicology”; RTG 2624, Project P7; Project Number 427806116). MT was funded by the Austrian Science Fund FWF (F7301). P.A.D. was supported by NIH R01 DK140485. SH was supported by the BMBF (031L0257J, 031L0256C, 031L0314I, 031L0313C); DFG (HO4772/5-2); and EU (ARTEMIS/101136299).

## Authors’ contributions

AG and JGH: study concept and design, data acquisition, analysis and interpretation of data, manuscript writing, funding, study supervision; MM (Myllys), DG, ZH, RH, Hannah S, RH (Hendawi) BBT, JR (Reinders), KD, MV, AS, NA, MV, TA, BH: contributed to study concept and design, data acquisition, manuscript writing, analysis and interpretation of data; AF, QS, DZ, SH: image analysis, contributed to manuscript writing, critical revision of the manuscript; JD, LA, KM, JR: bioinformatics analysis, contributed to manuscript writing, critical revision of the manuscript; UH, MS: bile acid analysis, contributed to manuscript writing, critical revision of the manuscript; ES, PÅ, EL: synthetized AS0369, contributed to study concept and design, manuscript writing, analysis and interpretation of data; KE: RNA-sequencing, contributed to bioinformatics analysis, study concept and design, data acquisition, analysis and interpretation of data, critical revision of the manuscript; TL, GS, Hartmut S, SJK, BS, MM (Mandorfer), MT, PAD: contributed to study concept and design, analysis and interpretation of data, critical revision of the manuscript.

## Data availability

All data presented in this manuscript will be made available to other researchers upon request. The raw RNA-sequencing data can be accessed via the Sequence Read Archive (SRA) with the accession number PRJNA1224581.

## Conflict of interest

AG and JGH have advised for Albireo. M.M. served as a speaker and/or consultant and/or advisory board member for AbbVie, AstraZeneca, Collective Acumen, Eli Lilly, Gilead, Echosens, Ipsen, Takeda and W. L. Gore & Associates and received grants from Echosens as well as travel support from AbbVie and Gilead. MT has received research grants from Albireo, Alnylam, Cymabay, Falk, Genentech, Gilead, Intercept, MSD, Takeda and Ultragenyx and travel grants from AbbVie, Falk, Gilead Intercept and Jannsen; he further has advised for AbbVie, Albireo, Agomab, BiomX, Boehringer Ingelheim, Chemomab Falk, Genfit, Gilead, Hightide, Intercept, Ipsen, Janssen, MSD, Mirum, Novartis, ProQR Therapeutics Phenex, Pliant, Regulus, Siemens and Shire and has served as speaker for Albireo, BMS, Boehringer Ingelheim, Falk, Gilead, Intercept, Ipsen, Madrigal and MSD. He is a co-inventor of patents for the medical use of norUDCA (nor-ursodeoxycholic acid/norucholic acid) filed by the Medical Universities of Graz and Vienna (service inventions). Outside of the submitted work, MS received funding from Green Cross WellBeing, Gilead Sciences, Robert Bosch GmbH, CORAT Therapeutics, HepaRegeniX GmbH, Boehringer Ingelheim, and Agena Bioscience. GS declares consulting or advisory board activities for Albireo, Ipsen, Sanofi, CSL Behring, Ideogen and Advanz Pharma and travel grants/educational support from Alnylam, Falk and Gilead. PAD has received research grant support from Albireo.

Please refer to the accompanying ICMJE disclosure forms for further details.
