# Supplementary material for: Stage-dependent effects of systemic ASBT inhibition in a cholestasis-induced cholemic nephropathy mouse model
Source: JHEP Rep. 2025 Sep 23;7(12):101599. doi: 10.1016/j.jhepr.2025.101599 (PMC12657750; doi:10.1016/j.jhepr.2025.101599)
Supplement: Multimedia component 1 [file mmc1.docx]

**Supplementary data to**

**Stage-dependent Effects of Systemic ASBT Inhibition in a Cholestasis-induced Cholemic Nephropathy Mouse Model**

Ahmed Ghallab, Maiju Myllys, Daniela González, Adrian Friebel, Zaynab Hobloss, Reham Hassan, Hannah Schmidt, Qasim Siddiqui, Deng Zhipeng, Rama Hendawi, Brigitte Begher-Tibbe, Joerg Reinders, Katharina Derksen, Ute Hofmann, Julia C Duda, Lucia Ameis, Kathrin Möllenhoff, , Abdellatief Seddek, Noha Abdelmageed, Ellen Strängberg, Peter Åkerblad, Mihael Vucur, Tom Luedde, Guido Stirnimann, Matthias Schwab, Tahany Abbas, Benedikt Hild, Hartmut Schmidt, Saul J Karpen, Benedikt Simbrunner, [Mattias Mandorfer](https://pubmed.ncbi.nlm.nih.gov/?term=Mandorfer+M&cauthor_id=37939855), Jörg Rahnenführer, Karolina Edlund, Stefan Hoehme, Michael Trauner, Paul A Dawson, Erik Lindström, Jan G Hengstler

Table of Contents

Supplementary methods 3

Supplementary Figures 9

Supplementary Tables 23

Supplementary references 26

# **Supplementary methods**

***Induction of obstructive cholestasis in mice and administration of the systemic ASBT inhibitor.*** Eight-to-ten-week-old male C57BL/6N mice (Janvier Labs, France) were used. Obstructive cholestasis was induced by ligating the extrahepatic bile duct (BDL) at a position between the gallbladder and the duodenum. Sham control mice underwent the same operative procedure but without BDL. The BDL mice received AS0369 (60 mg/kg) or vehicle (0.5% methyl cellulose and 0.1 % tween 80) orally by gavage twice per day for 4 weeks starting on either day 3, 21, 42, or 63 after the surgery. The sham operated mice received AS0369 for 4 weeks starting on day 3 after the surgery or vehicle starting on either day 3, 21, 42, or 63 after the surgery.

***Sample collection and processing.* *Blood sampling.*** Heart blood samples were collected from anaesthetised mice in syringes precoated with heparin and used freshly for analyses of liver damage biomarkers. The remaining blood samples were centrifuged, and the isolated plasma were stored at -80 °C until used for analysis. ***Urine sample collection.*** 24-hour urine was collected in single mouse metabolic cages (Tecniplast Deutschland GmbH, Germany) and stored at -80 °C until used for analysis. ***Tissue sample collection.*** Liver and kidney tissue samples were collected from defined anatomical positions and processed using standard protocols as previously described [1]*.*

***Biochemical analysis.*** Analysis of total bilirubin, alanine transaminase, aspartate transaminase, albumin, blood urea nitrogen and alkaline phosphatase was done in freshly collected heparinized heart blood using the Piccolo Xpress Chemistry Analyzer and the Piccolo General Chemistry 13 Panel Kit.

***Bile acid assay.*** Concentrations of bile acids in liver and kidney tissues were determined by negative electrospray (ESI) liquid chromatography tandem mass spectrometry (LC-MS/MS) in multiple-reaction-monitoring (MRM) mode on an Agilent 6495B triple quadrupole mass spectrometer (Agilent, Germany) coupled to an Agilent Infinity II HPLC system as described previously [2, 3]. Bile acid analysis in bile, blood plasma and urine was accomplished by LC-MS as published previously [4]. Briefly, the urine and bile samples were diluted 10-fold and 100-fold with water for analysis of bile acids while plasma samples were used undiluted. Proteins were precipitated with 80% methanol containing internal standards. After centrifugation, 5 µL of the supernatant were injected for LC-MS-measurement on a QExactive mass spectrometer coupled to a Vanquish Horizon UHPLC (ThermoFisher). Quantification was done using the Skyline software (version 24.1).

***MALDI-MS-Imaging and quantifications.*** MALDI-MS images of taurocholic acid (TCA) were acquired on a timsTOF fleX (Bruker Daltonics, Bremen, Germany) as published earlier [1]. Briefly, 5 µm-thick frozen liver and kidney tissue sections were sprayed with 2-mercaptobenzothiazole and measured in negative mode over a mass range of 85-800 m/z and internally calibrated on taurocholic acid and the 2-mercaptobenzothiazole matrix peak. Data was evaluated using the Scils Lab MVS software (version 2024b Pro, build 12.01.16059). Since TCA and TMCA have the same sum formula (same m/z), the here reported TCA-signal is indeed the sum of TCA and TMCA.

***NGAL, KIM1, and cystatin C assays.*** Concentrations of NGAL/Lcn-2 were analyzed in mouse plasma using the Mouse Lipocalin-2/NGAL DuoSet ELISA kit plus the DuoSet ELISA Ancillary Reagent Kit 2 from R&D systems according to manufacturer’s protocol. Concentrations of Kim-1 were determined in mouse plasma using the Mouse TIM1 ELISA Kit (KIM-1) from Abcam according to the manufacturer’s protocol. Concentrations of cystatin C were determined in plasma using the Mouse Cystatin C ELISA Kit from Abcam according to manufacturer’s protocol.

***Histopathology.*** Histopathological analysis was performed in 4 µm-thick paraformaldehyde-fixed paraffin-embedded liver and kidney tissue sections. Hematoxylin and eosin (H&E) staining was performed using the Discovery Ultra Automated Slide Preparation System [1, 5]. Sirius red staining was done using a commercially available kit, according to the manufacturer’s instruction [6]. Whole slide scans were acquired (Axio Scan.Z1) for the quantifications and representative snapshots are shown in the result section.

***Immunohistochemistry.*** Immunostainings were performed in 4 µm-thick paraformaldehyde-fixed paraffin-embedded liver or kidney tissue sections using the Discovery Ultra Automated Slide Preparation System, as previously described [1]. The used antibodies and staining conditions are given in Suppl. Table 1. Whole slide scans were acquired (Axio Scan.Z1) for the quantifications and representative snapshots are shown in the result section.

***Image analysis.*** Image processing and quantification of series of brightfield tissue slides stained with H&E, Sirius Red, CK19, MECA-32, ASBT, NTCP, Ki-67, and CD13, was conducted to segment relevant regions of interest (ROI) and investigate region-specific features as described below:

For the H&E, ASBT, and MECA-32 staining, tissue and ROI segmentation was performed interactively with QuPath [7] using Random Trees R-Tree [8] or Artificial Neural Network [9] based Pixel classifiers, working at 1.77 μm/px (ROI) / 14.15 μm/px (Tissue) resolution on the Eosin channel (H&E staining), and at 0.22/0.88 μm/px resolution on the DAB channel (ASBT and MECA-32 staining).

For the Sirius Red, CK19, NTCP, and CD13 staining, U-Net convolutional neural networks [10] were trained and deployed for the automated semantic segmentation of tissue and ROIs, using the nnU-Net framework [11]. Training data was generated interactively using QuPath for representative image subsets and downscaled by a factor of two (0.44 μm/px) due to GPU memory limitations for U-Net training. The glomeruli segmentation model was trained with data from H&E and MECA-32 staining in order to focus on structural aspects for a staining independent model, improving generalization.

Nuclei segmentation in Ki-67 staining was performed using the StarDist [12] algorithm. For subsequent classification into Ki-67+/-nuclei, an ANN-based object detector was interactively trained in QuPath.

Quantification of H&E, Sirius Red, CK19, and MECA-32 was implemented as relative ROI area measurement normalized by tissue area. For Ki-67 quantifications, the density of Ki-67+ nuclei were computed.

For the H&E staining, glomeruli with dilated Bowman’s space (BS) were defined as glomeruli with BS area exceeding Q3+1.5×(Q3−Q1), where Q3 and Q1 represent the upper and lower quartiles of the sham controls (from all analysed time periods) distribution. The number of dilated glomeruli was normalized by tissue area.

To assess bile canaliculi (BC) diameters in the CD13 staining, a connectivity preserving medial axis skeleton was computed for each BC object using a thinning algorithm [13]. For each skeleton pixel, its distance to the boundary was measured. These measurements provided a comprehensive representation of BC diameter. To minimize the influence of outliers and false-positive segmentations, any diameter measurements above the 99th percentile were discarded. The dilated diameters were defined as BC with diameter exceeding Q3+1.5×(Q3−Q1), where Q3 and Q1 represent the upper and lower quartiles of the sham controls (from all analysed time periods) distribution.

***Gene expression analysis***

***RNA isolation and cDNA synthesis.*** RNA was isolated from snap-frozen liver and kidney tissue samples using RNeasy Mini Kit, followed by cDNA synthesis using the High-Capacity cDNA Reverse Transcription Kit. ***qRT-PCR assays.*** Quantitative RT-PCR analyses were performed with cDNA using TaqMan 7500 Real-Time PCR, TaqMan universal PCR Master Mix, and TaqMan gene expression assays (Suppl. Table 2). The data were normalized to the housekeeping gene GAPDH, and gene expression changes were calculated using the ΔΔCt method. The values were expressed as fold changes over the corresponding control samples.

***RNA-sequencing.*** RNA was isolated from liver and kidney tissue using the RNeasy Mini Kit (Qiagen). RNA integrity was assessed on a 2100 Bioanalyzer with the RNA 6000 Nano Kit (Agilent Technologies); all samples had an RNA Integrity Value (RIN) above 8 or slightly below (7.8, 7.9 and 7.9). RNA concentrations were measured on a Qubit 4 Fluorometer with the RNA BR Assay Kit (Thermo Fisher, Waltham, USA). Sequencing libraries were generated from 500 ng RNA, using the TruSeq Stranded mRNA Kit with unique dual indexes (Illumina, San Diego, CA, USA), according to the manufacturer’s protocol. Quantification of the final libraries was performed with the Qubit 1X dsDNA HS Assay Kit (Thermo Fisher, Waltham, USA), and library sizes were checked on an Agilent 2100 Bioanalyzer with the DNA 1000 Kit (Agilent Technologies, Santa Clara, CA, USA).

The libraries were then normalized, pooled, diluted to 1.015-1.05 pM, and paired end sequenced (bp) using the 500/550 High Output Kit v2.5 (Illumina, San Diego, CA, USA) on an Illumina NextSeq 550.

***Bioinformatics.*** The transcript quantification and mapping of the FASTQ files were pre-processed with the software salmon, using option ‘partial alignment’ and the online provided decoy-aware index for the mouse genome [14]. All further analyses were performed with R, version 4.4.1 [15]. Transcript reads were summarized on gene level using the R package tximeta [16]. The following analyses were performed identically, but independently on the kidney and liver samples. For pre-filtering, from all 35,727 genes that have reads, those with less than 10 reads across all mice were removed from the data, such that 20,982 genes for kidney and 19,306 genes for liver remained. Principal component analysis was performed using the top 1,000 most variable genes after variance stabilization of the gene counts. Differential gene expression analysis was calculated using the R package DESeq2 [17]. A general linear model with one factor that combines the treatments “Sham + vehicle”, “BDL + vehicle” and “BDL + AS0369” with the time points day 3, day 21, day 42, and day 63 was fitted to calculate differentially expressed genes (DEGs). This leads to a single factor model with 12 levels “Sham + vehicle, day 3”, “Sham + vehicle, day 21”, …, “BDL + AS0369, day 42”, “BDL + AS0369, day 63”. DEGs were calculated for the comparisons “Sham + vehicle vs. BDL + vehicle” at each time point. For more reliable effect estimates, adaptive shrinkage was applied [18]. This leads to shrinkage of log-2 transformed fold changes (log_2_(FCs)) towards zero if expression changes are mostly due to noise, whereas relevant log_2_(FCs) are preserved. For each comparison, a gene is finally counted as DEG if the effect size fulfils log_2_(FC) > log_2_(1.5) for upregulation (log_2_(FC) < -log_2_(1.5) for downregulation) and the estimate is significantly different from zero (i.e. no effect) with a false discovery rate (FDR)-adjusted p-value p_adj_ < 0.05. Enrichment analysis for biological processes gene ontology (GO) terms was applied separately for up and down regulated genes for the above-described comparison “Sham + vehicle vs. BDL + vehicle” for the first and last time point and of each area defined in the differentiation pattern (DiPa) plot for all time points. The R package topGO, version 2.56.0, with the elim algorithm was used to focus on more specific biological process GO groups [19]. P-values of GO groups were FDR-adjusted and considered significant if the adjusted p-value was smaller than 0.05. The DiPa plot was inspired by previous works of [20]. It defines the gene groups unaffected (ua), 1a/1b, 2a/2b and not treatable a (nta) and not treatable b (ntb) by comparing the log_2_(FC)s of the comparisons BDL + vehicle vs. Sham + vehicle (x-axis) and BDL + AS0369 vs. Sham + vehicle (y-axis). Using *thres* = log_2_(1.5), the areas are defined as:

- ua: (|x| < thres and |y| < thres) or (|x| < thres and |x-y| < thres)
- 1a: x > thres and |y| < thres and y<x –thres
- 2a: x > thres and y > thres and y<x – thres
- nta: x>thres and y>x-thres.

And accordingly, we define

- 1b: x < -thres and |y|<thres and y>x
- 2b: x < thres and y < -thres and y>x+thres
- ntb: x < thres and y<x+thres
- 3b: |x|<thres and y < -thres.

Genes in 1a that additionally fulfill the stricter |x|>7∙ log_2_(1.5) are considered extreme genes 1a (accordingly for 1b), as they are extremely affected by the BDL, but rescued by AS0369.

***Statistical analysis.*** Data were analyzed using GraphPad Prism version 10.4.0 Software. The statistical tests used are indicated in the figure legends.

# **Supplementary Figures**


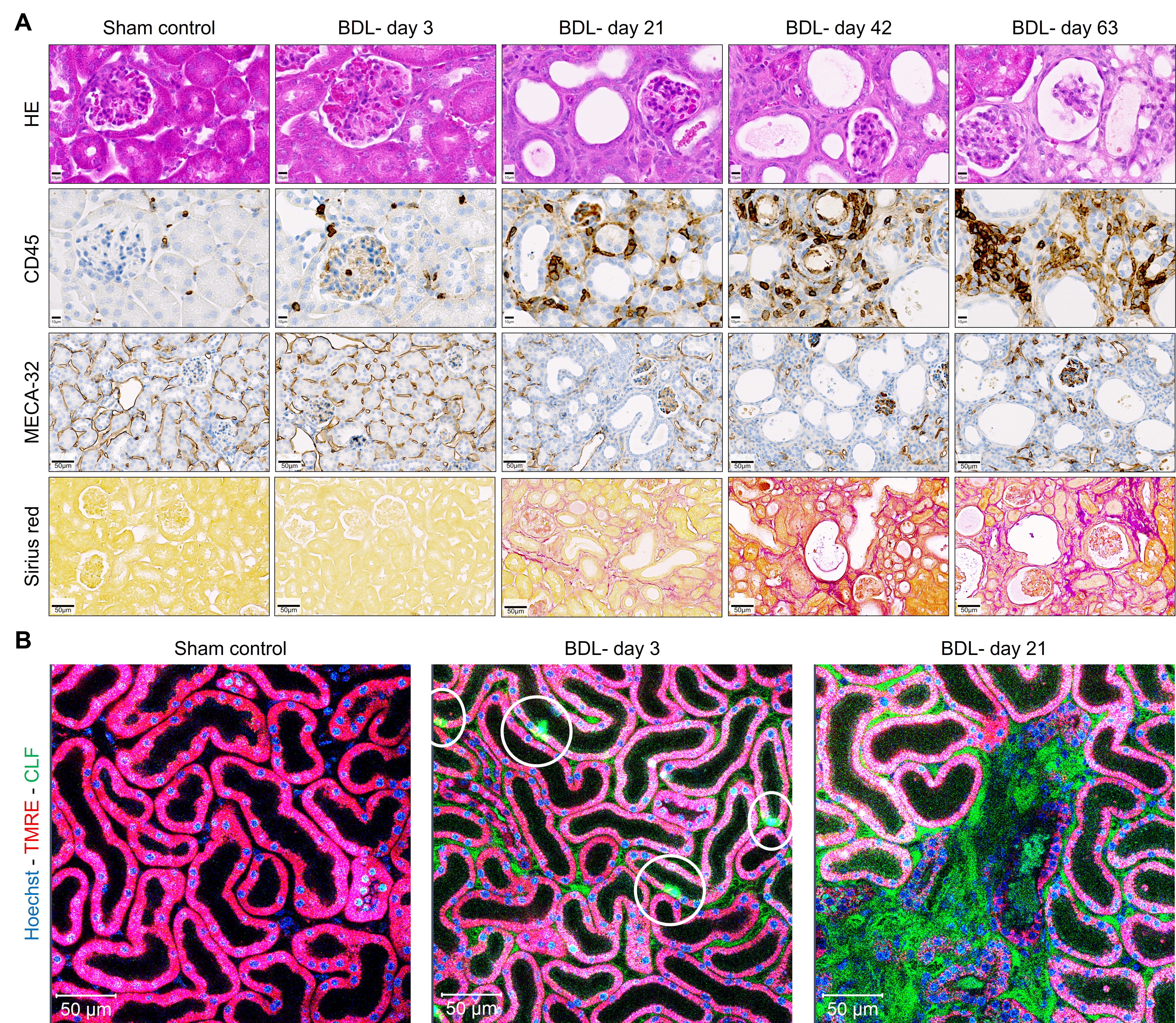


Suppl. Fig. 1: Renal tissue at days 3, 21, 42 and 63 after BDL to characterize the tissue before the onset of therapy. (A) H&E staining, immunostaining against the leukocyte marker CD45, the endothelial marker MECA-32 and staining of fibrotic structures by Sirius red. (B) Intravital imaging of the kidney. Hoechst dye for visualization of nuclei; tetramethylrhodamine ethyl ester (TMRE) for visualization of mitochondria; Cholyl-lys-fluorescein (CLF): green-fluorescent bile salt analogue; the circles on BDL-day 3 indicate CLF flooded dead cells.


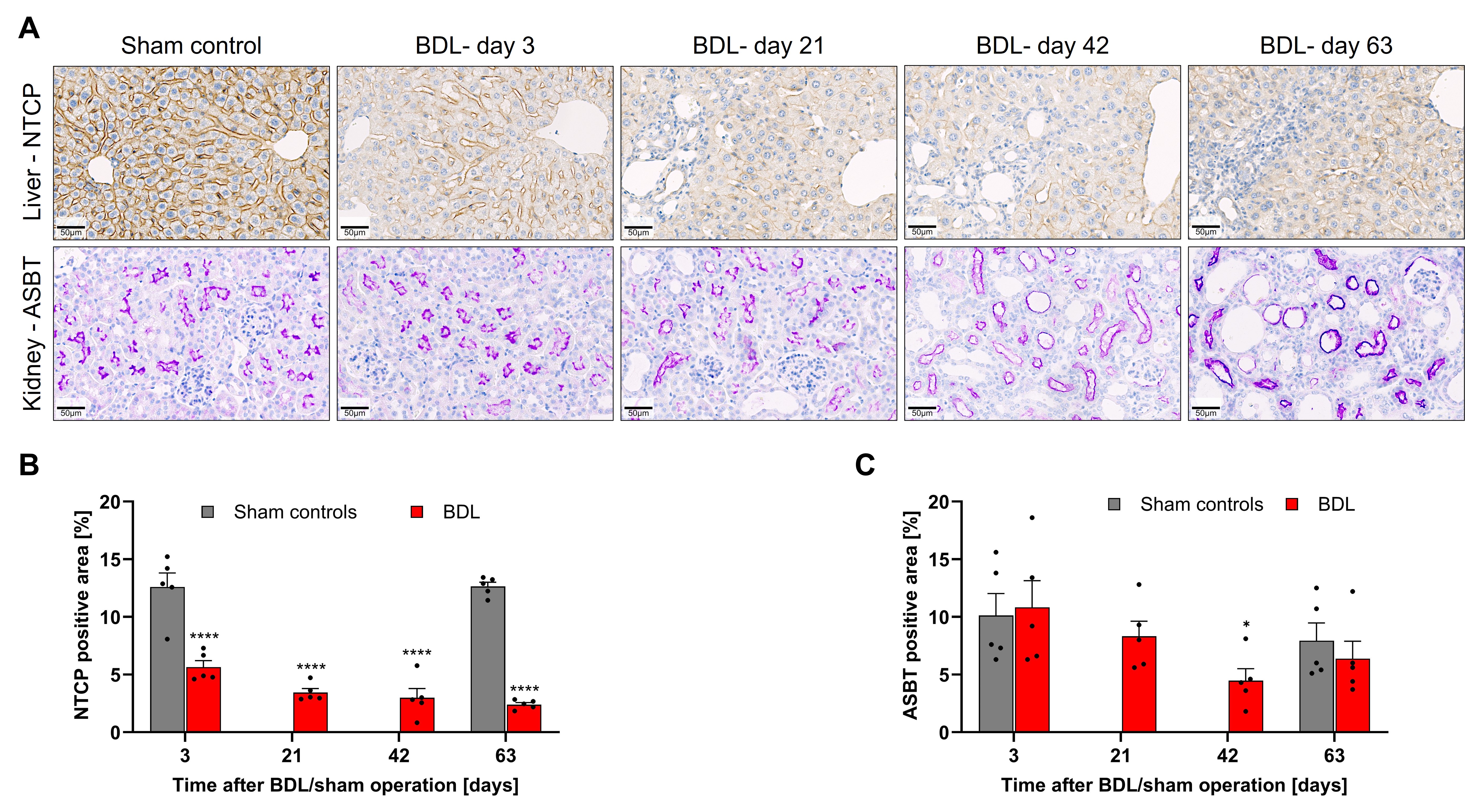
Suppl. Fig. 2: Expression of the bile acid uptake transporters NTCP in liver and ASBT in kidney tissue. (A) Immunostaining of liver sections against NTCP; (B) Immunostaining of kidney sections against ASBT; (C) Percentage of the NTCP positive area; (D) Percentage of the ASBT positive area. * p<0.05; **** p<0.0001, Šídák's multiple comparisons test.


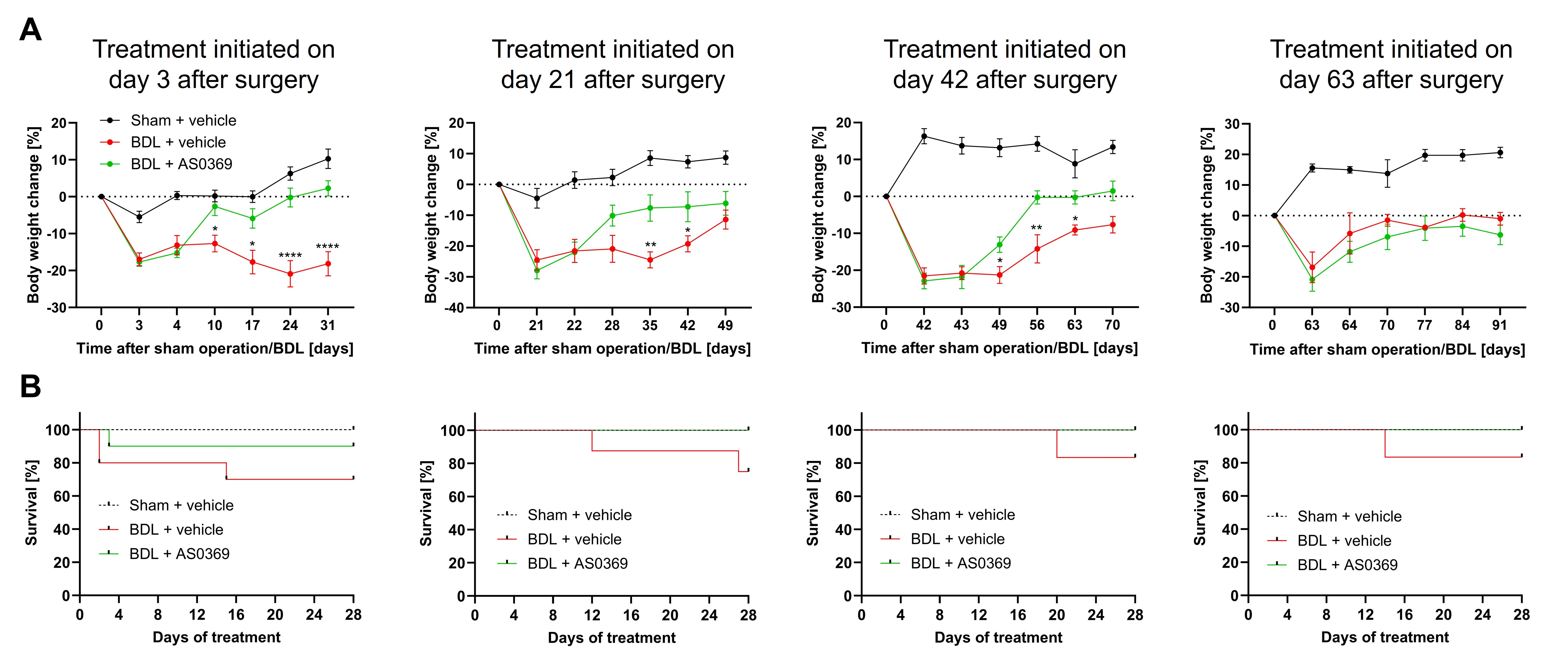


Suppl. Fig. 3: Influence of AS0369 treatment on body weight and survival. (A) body weight changes; **p* <0.05; ***p* <0.01; *****p* <0.0001 compared to the corresponding BDL + AS0369 group; Unpaired t test; (B) Survival during the 28-days treatment period.


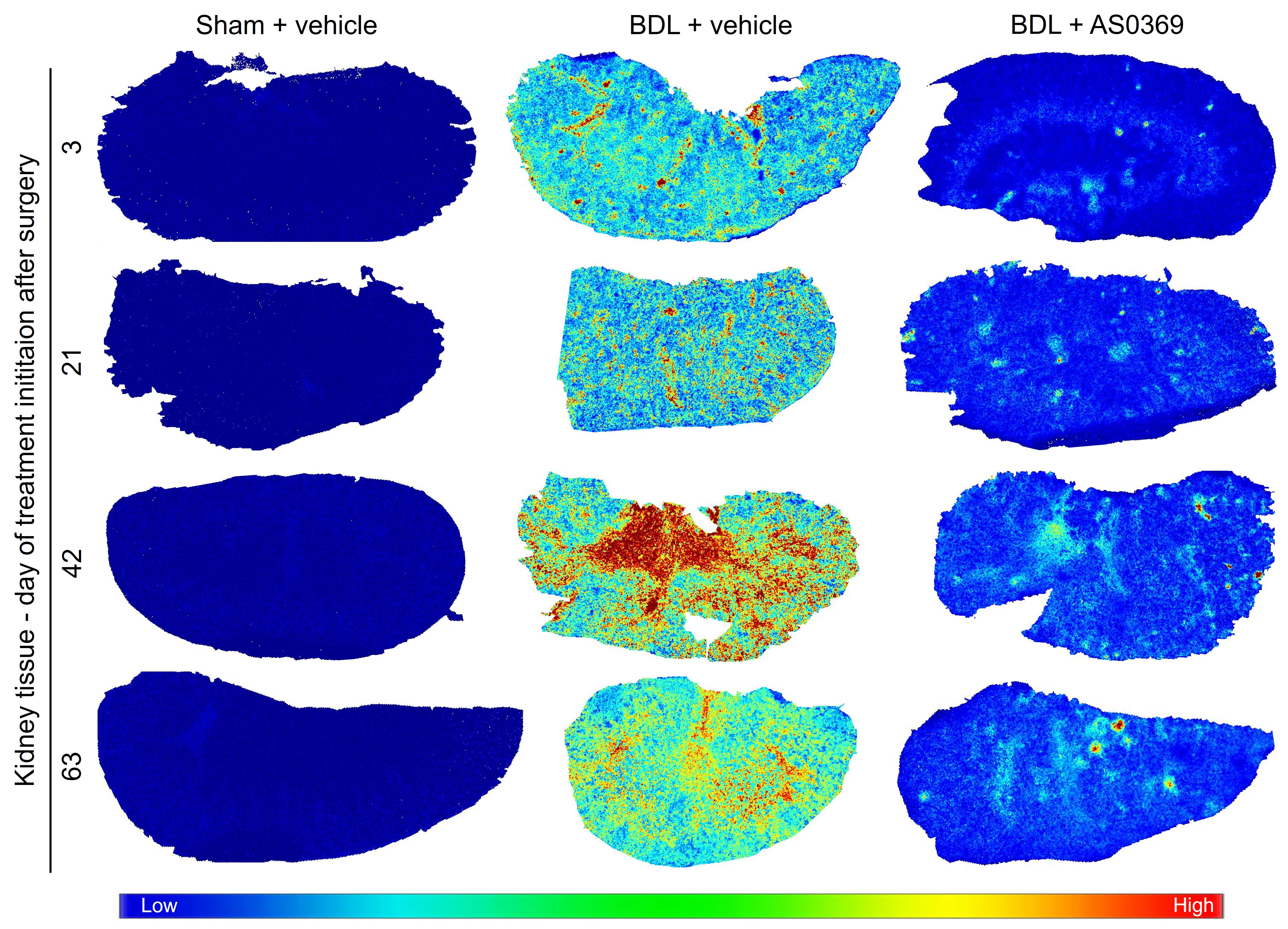


Suppl. Fig. 4: MALDI-MSI imaging of taurocholic acid (TCA) in renal whole organ sections.


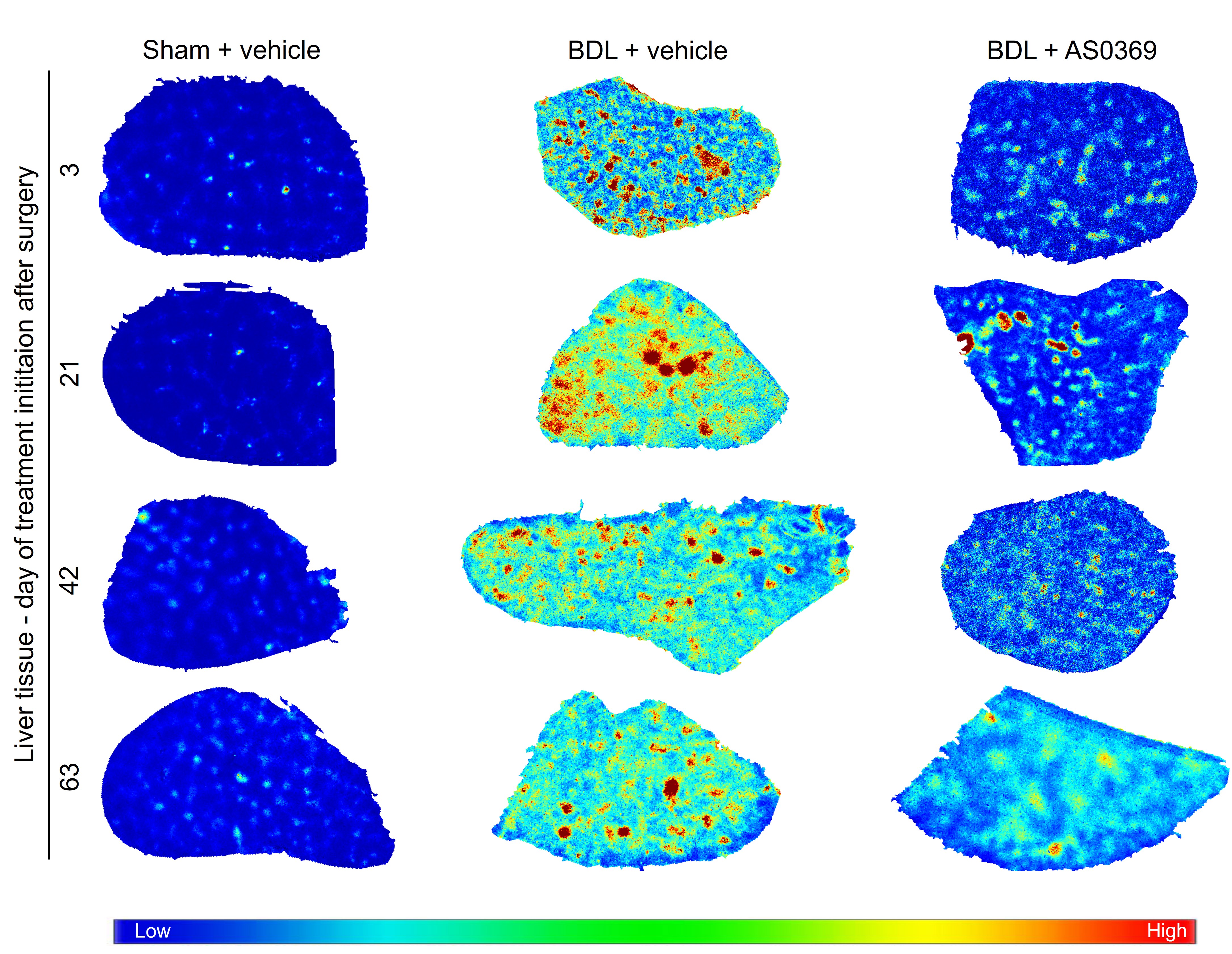


Suppl. Fig. 5: MALDI-MSI imaging of taurocholic acid (TCA) in whole slide liver tissue sections.


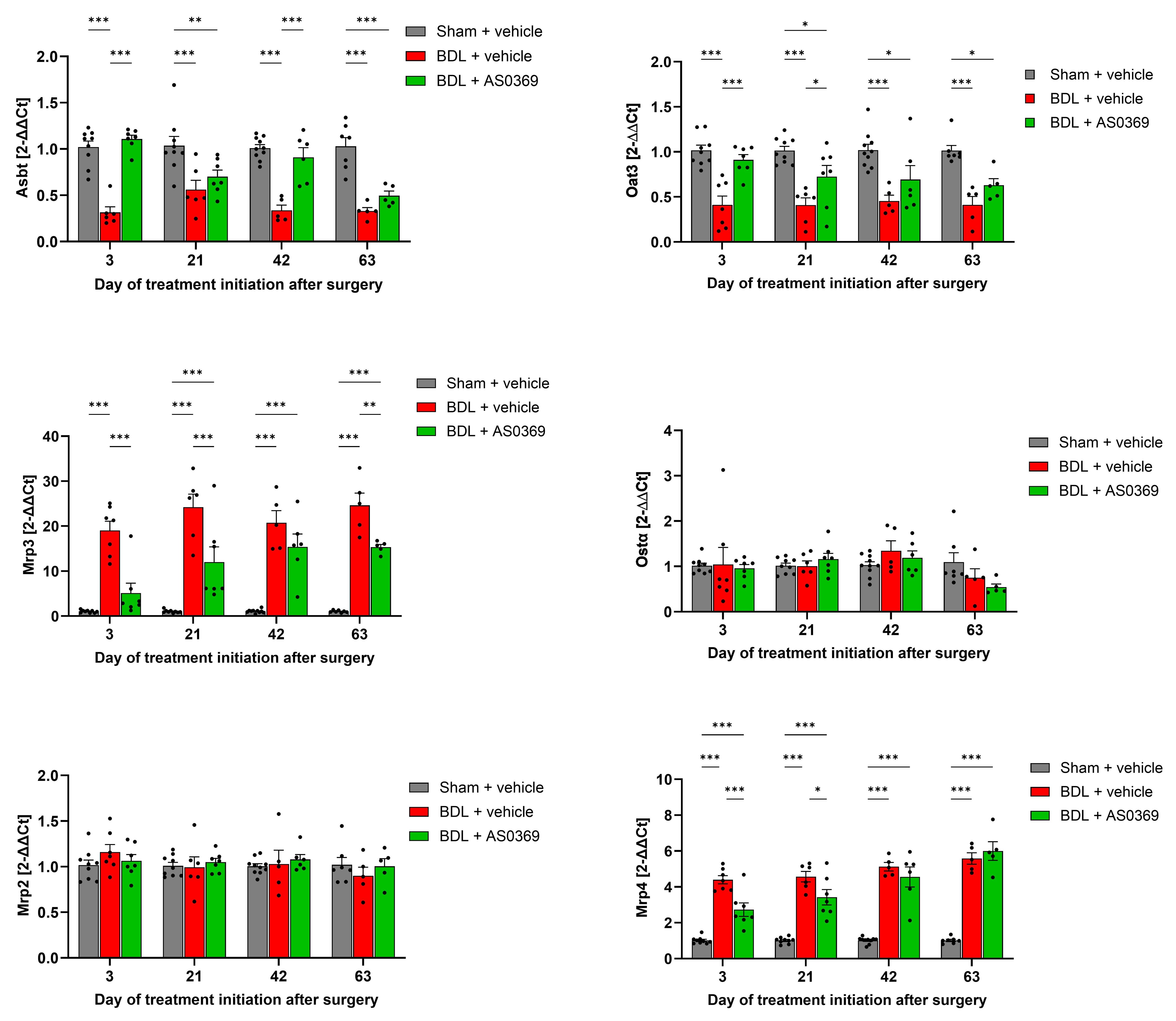
Suppl. Fig. 6: RNA levels of bile acid transporters in renal tissue homogenate analyzed by qRT-PCR. *p<0.05; **p<0.01; ***p<0.001; Tukey's multiple comparisons test.


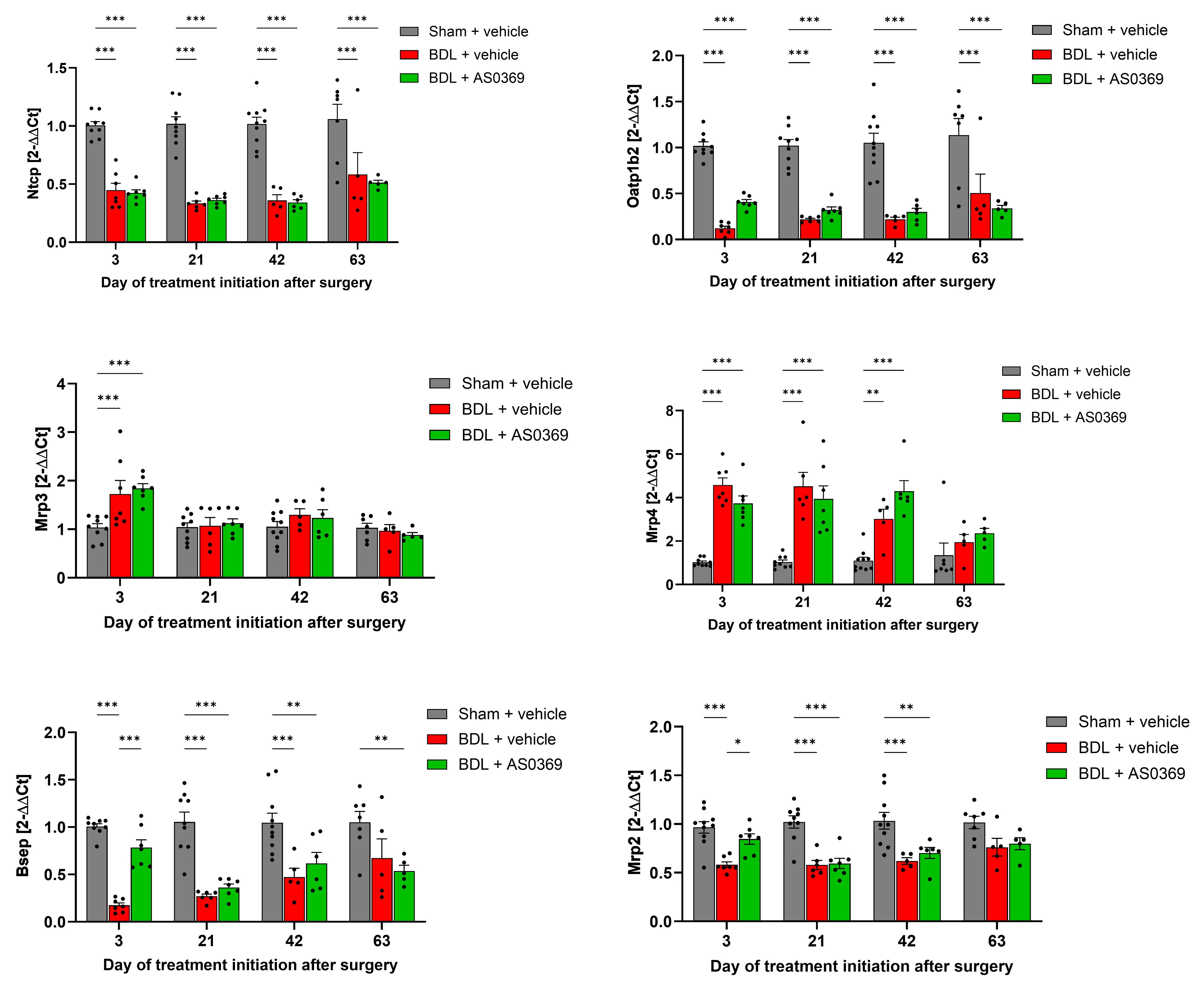
Suppl. Fig. 7: RNA levels of bile acid transporters in liver tissue homogenate analyzed by qRT-PCR. *p<0.05; **p<0.01; ***p<0.001; Tukey's multiple comparisons test.


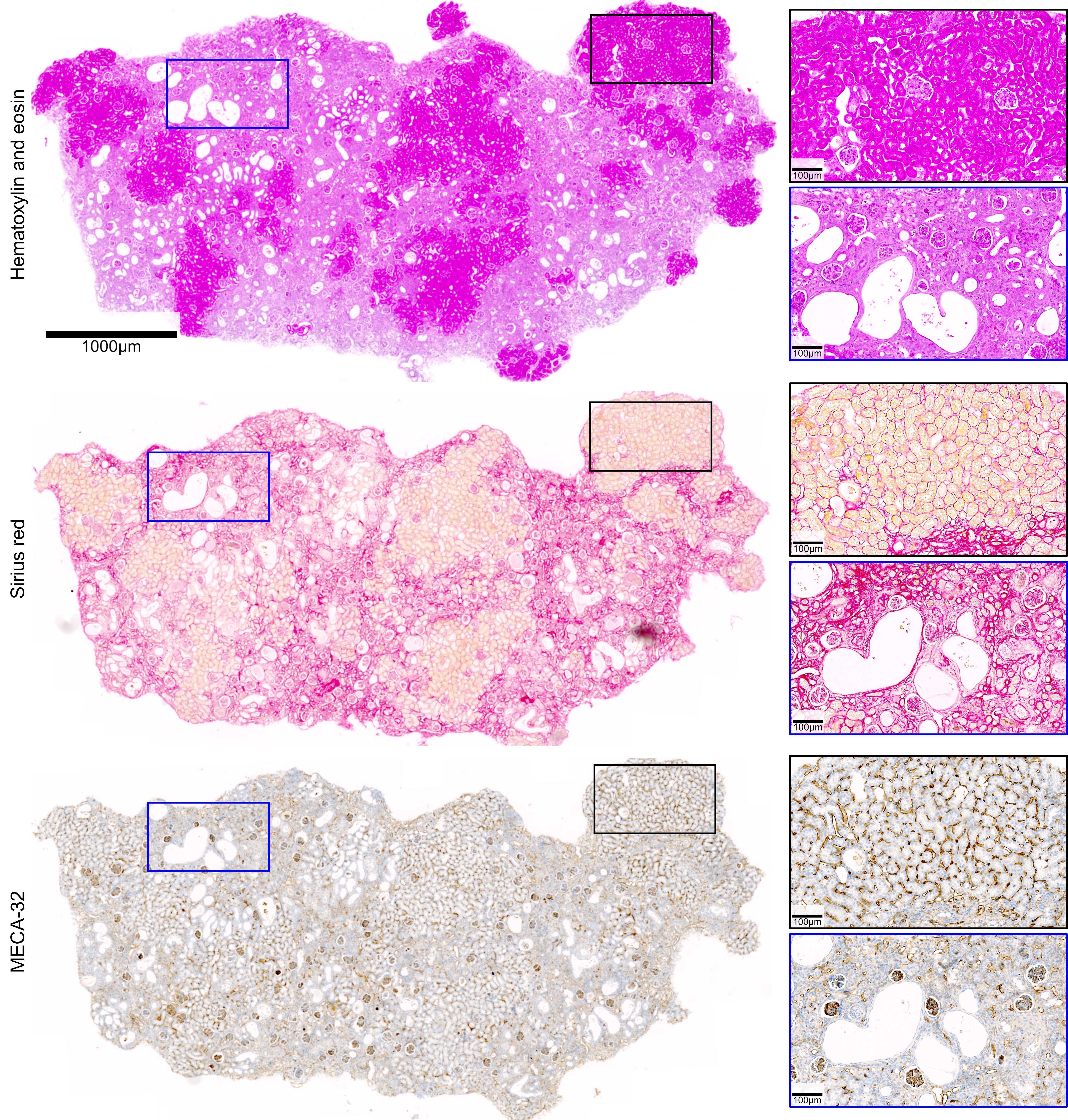


Suppl. Fig. 8. Hematoxylin and eosin staining of 10-week bile duct-ligated mouse; the pale eosin staining indicates damaged tissue. This fits well with the massive fibrosis (indicated by Sirius red staining) and damage of the peritubular capillaries (indicated by loss of MECA-32 staining).


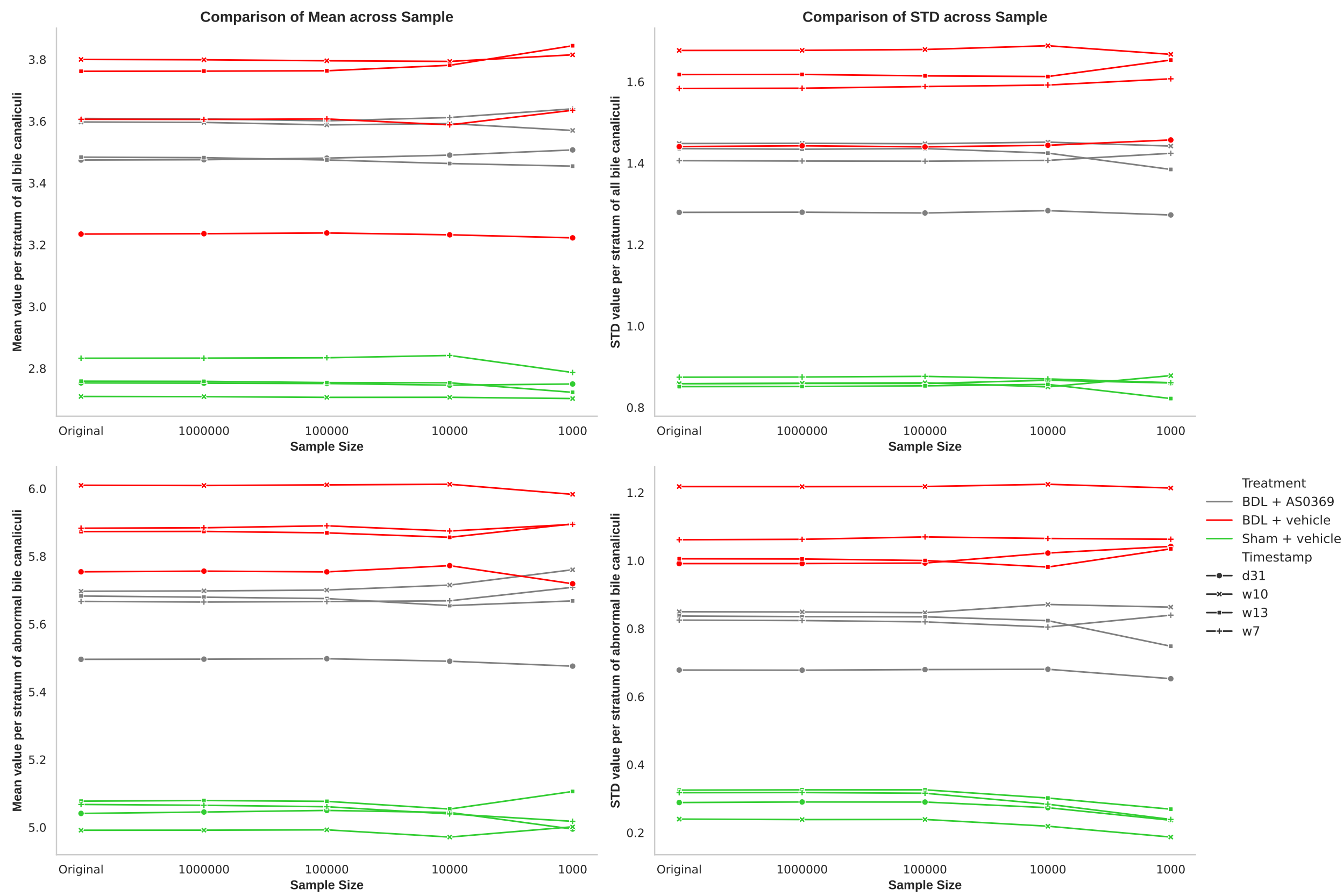


Suppl. Fig. 9. Consistency of mean and standard deviation under stratified stochastic sampling. The plots show the mean and standard deviation (STD) per experimental group for all bile canaliculi (top row) and abnormal bile canaliculi (bottom row), using decreasing sample sizes per group. Results remain consistent down to 10,000 samples, with only minor changes (maximum changes: mean 0.49%, STD 10.67%), but increase notably at 1,000 samples (maximum changes: mean 2.19%, STD 10.60%).


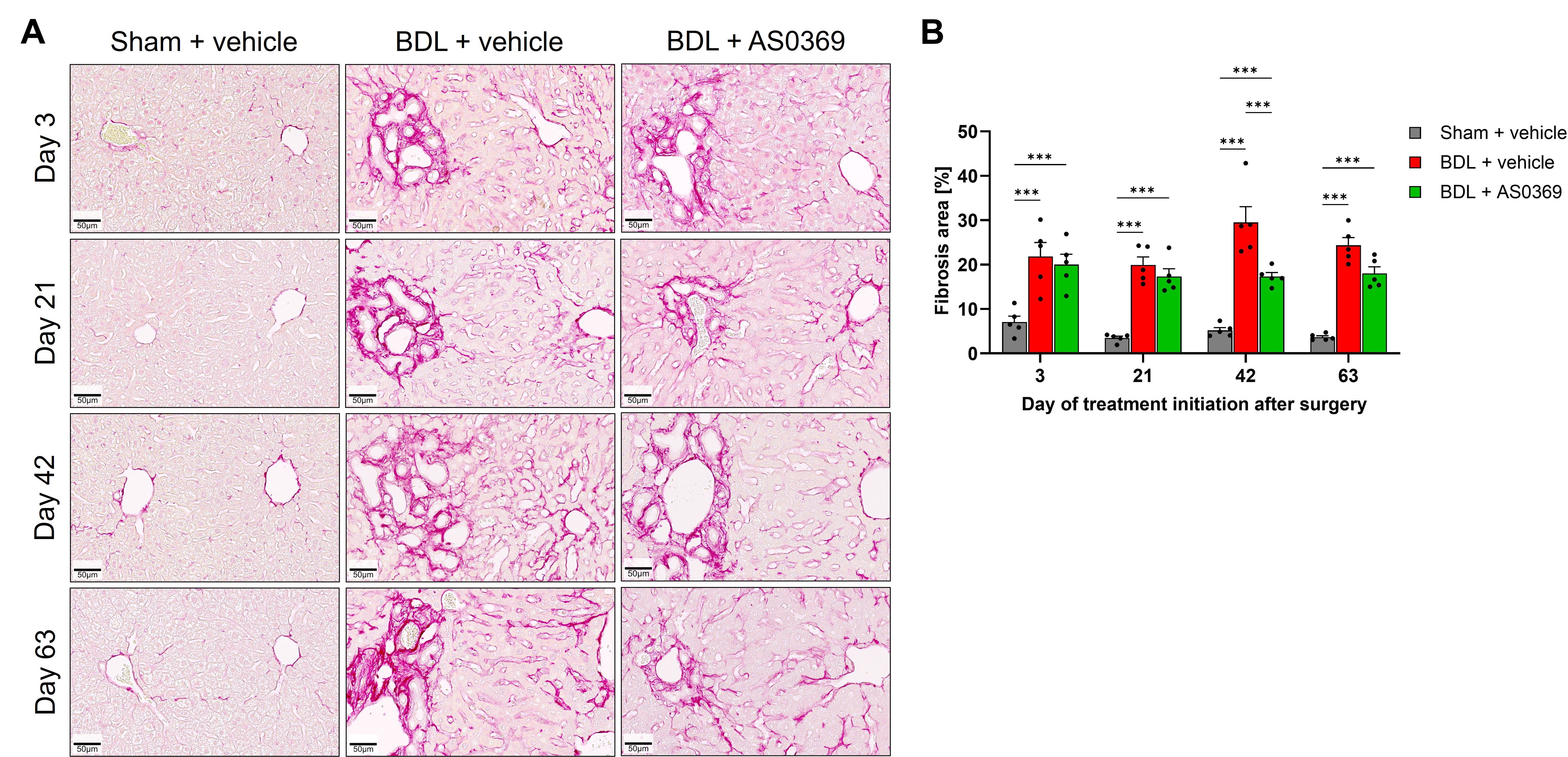


Suppl. Fig. 10: Fibrosis of liver tissue. (A) Sirius red staining visualizing periportal and perisinusoidal fibrosis. (B) Quantification of the Sirius red positive (fibrotic) area. *** p<0.001, Tukey's multiple comparisons test.


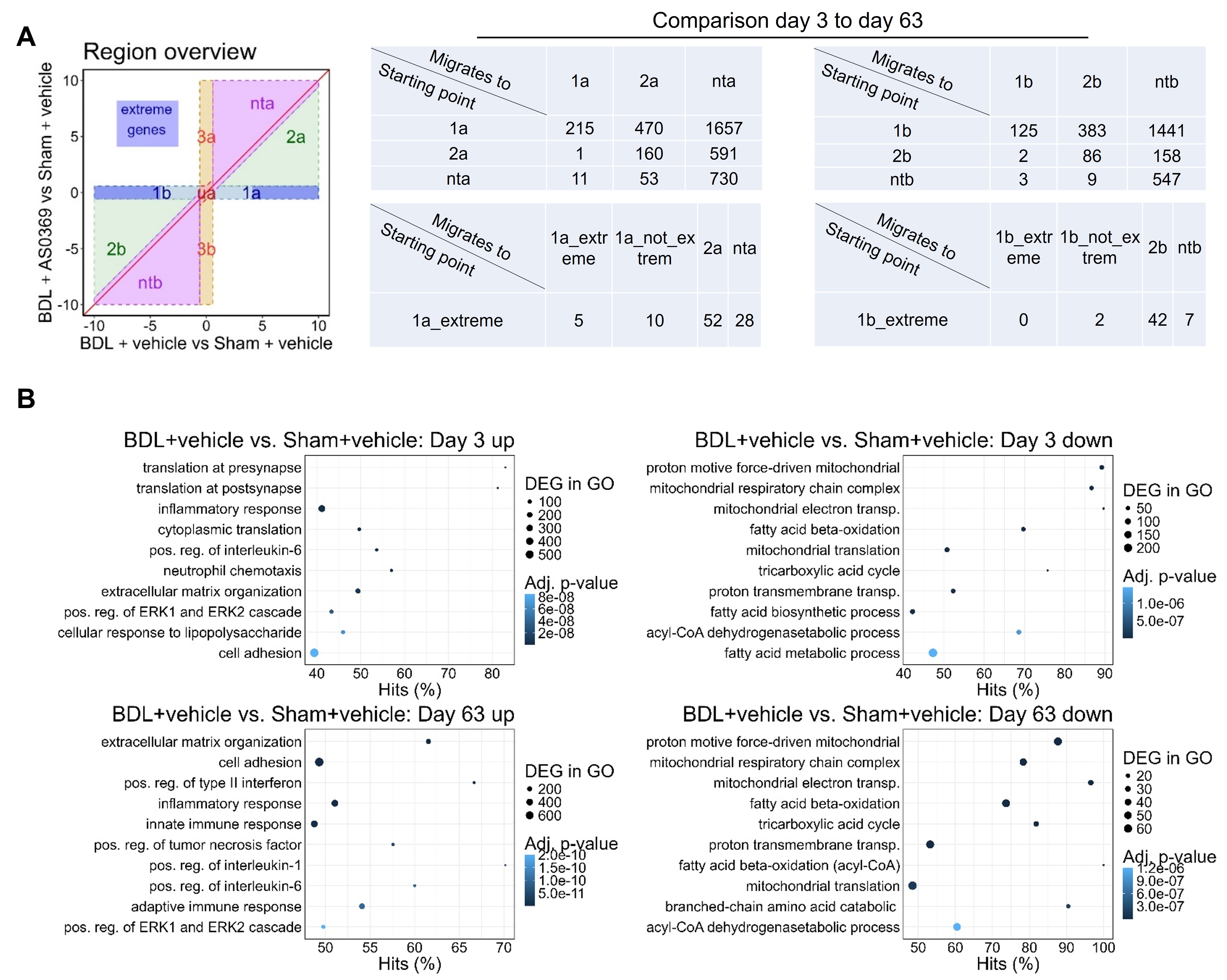
Suppl. Fig. 11: Gene expression migration and gene ontology (GO) analysis of RNA-seq data of kidney tissue homogenate. (A) DiPa-plot illustrating response to AS0369 therapy. Expression pattern groups 1a and 1b contain genes that are up or down regulated by BDL and are brought back to the normal range by AS0369 therapy. Expression pattern groups 2a and 2b contain genes up or down regulated by BDL that are improved but not completely back to the normal range by AS0369 therapy. nta and ntb represent genes for which AS0369 therapy did not lead to any improvement. The gene expression migration analysis (comparison day 3 to day 63) gives the number of genes in specific expression pattern groups of the day 3 treatment group and informs to which region they migrated in the day 63 treatment group. For example, genes (n=2342) that clustered to region 1a for the day 3 treatment/group clustered to nta (n=1657), 2a (n=470) or remained in 1a for the day 63 treatment group. Expression pattern groups “1a or 1b extreme” contain genes that were up or downregulated by BDL a factor of at least 17 (=1.5^7^). A relatively high fraction of genes in the “1a or 1b extreme” expression pattern groups for the day 3 treatment remained in 1a/b or migrated to 2a/b compared to the day 63 treatment group. (B) Gene ontology (GO) analysis of genes up or down regulated in day 3 or day 63 treatment groups.


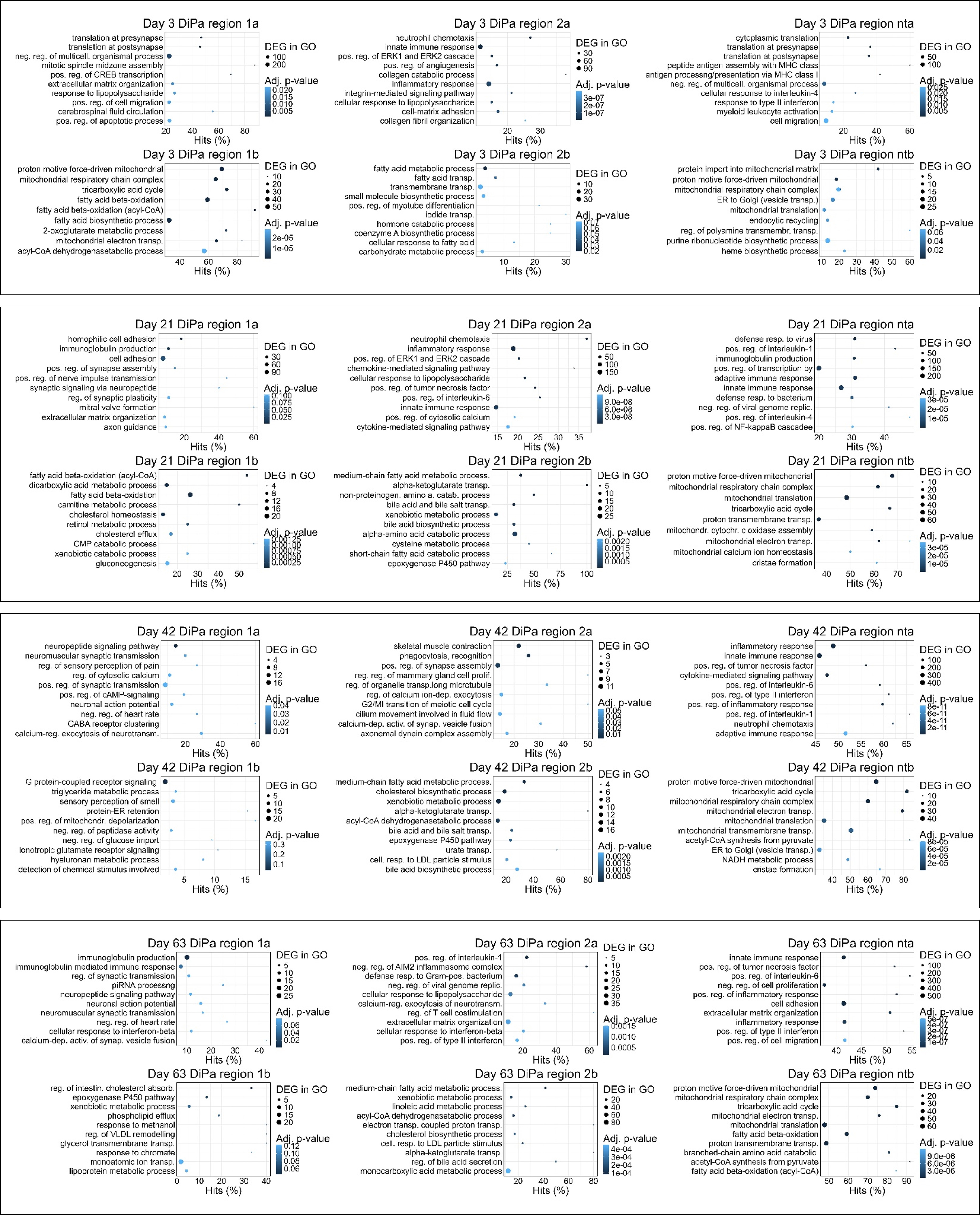


Suppl. Fig. 12: Gene ontology analysis of genes in the individual DiPa cluster regions for the RNA-seq analysis of kidney tissue homogenate.


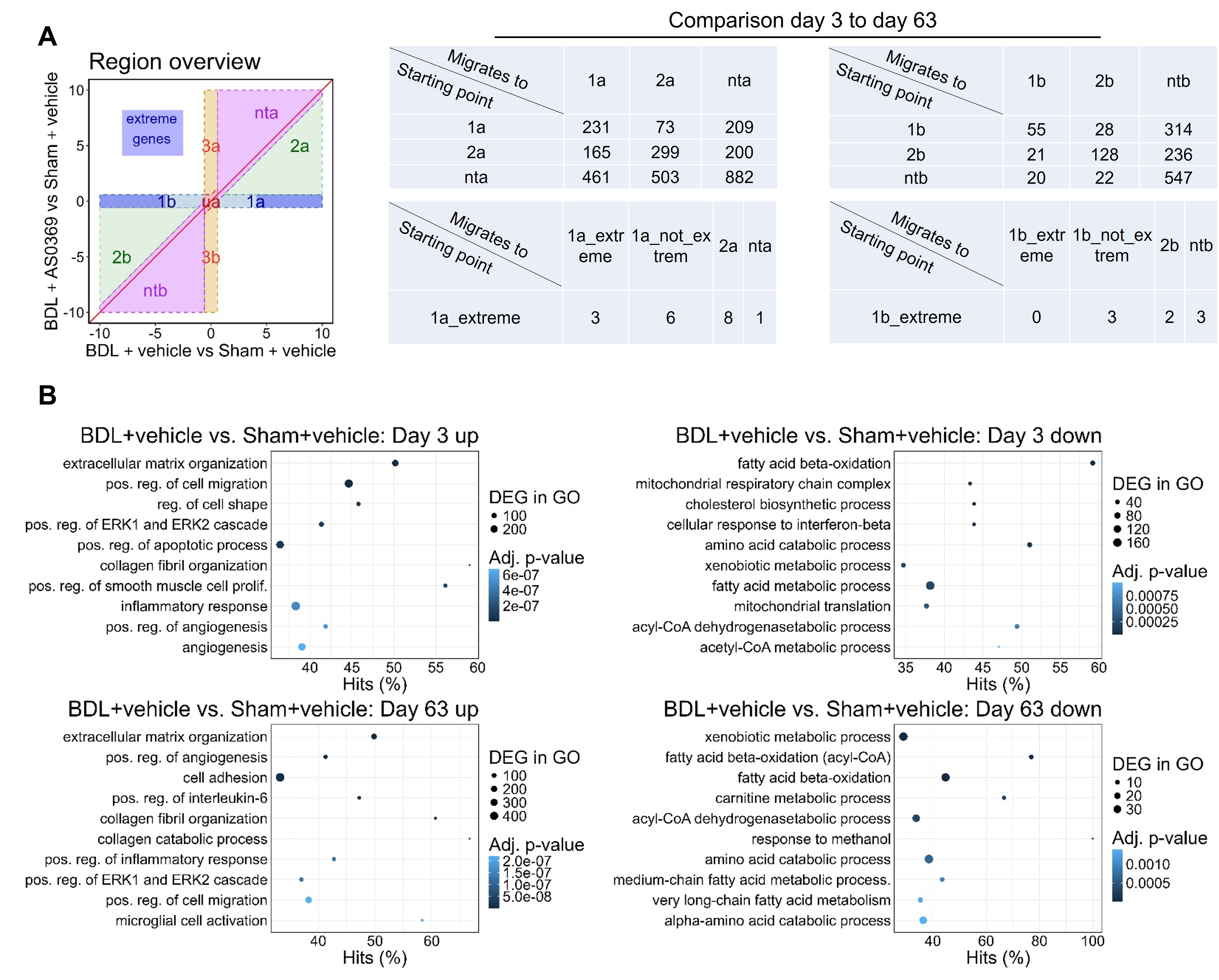
Suppl. Fig. 13: Gene expression migration and gene ontology analysis of RNA-seq data of liver tissue homogenate. The analysis corresponds to that shown for the kidney data in Suppl. Fig. 11.


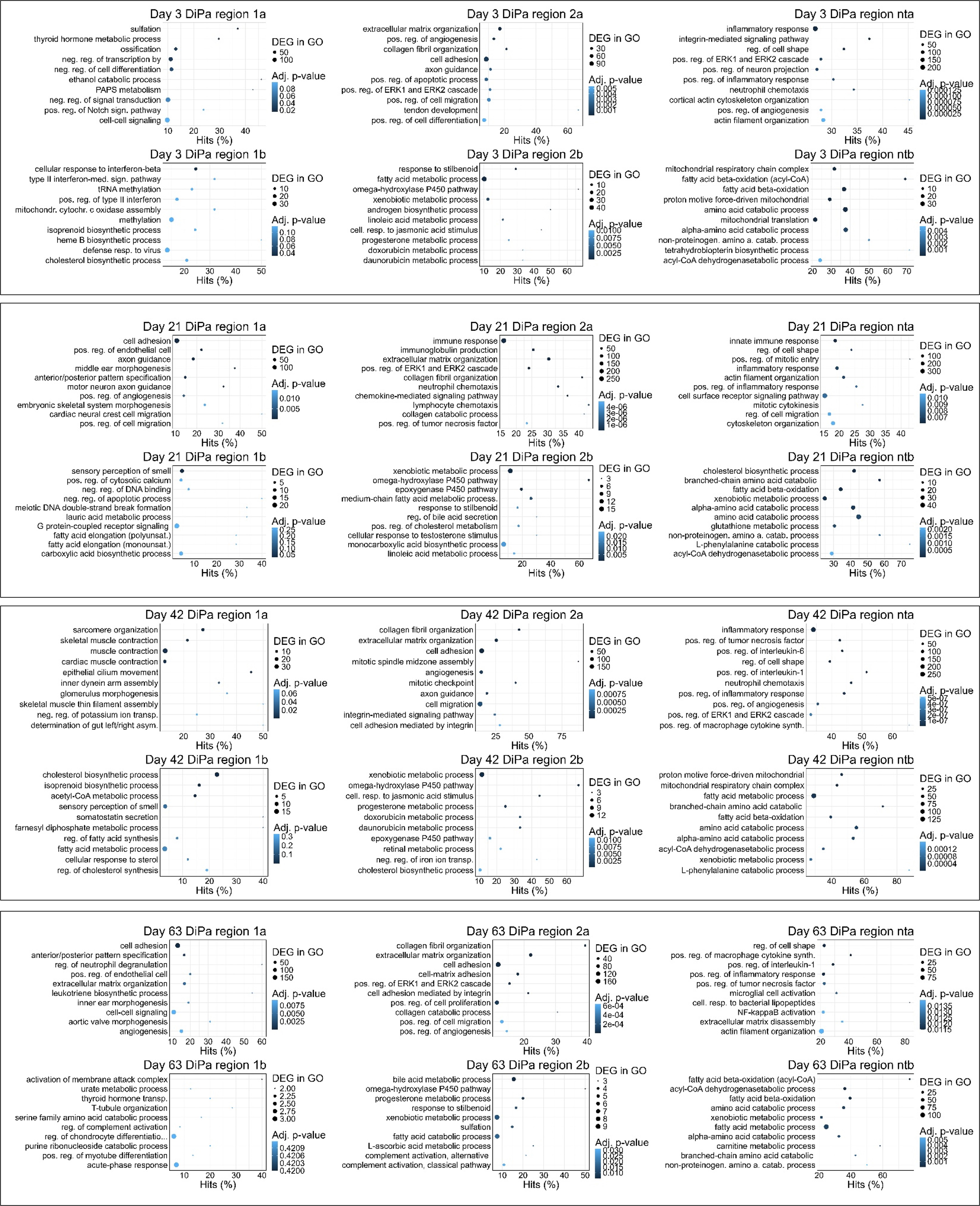
Suppl. Fig. 14: Gene ontology analysis of genes in the individual DiPa expression pattern groups for the RNA-seq analysis of liver tissue homogenate.

Suppl. Fig. 15. Systemic ASBT inhibition caused no major histological alteration either in the liver (A) nor in the kidneys (B).
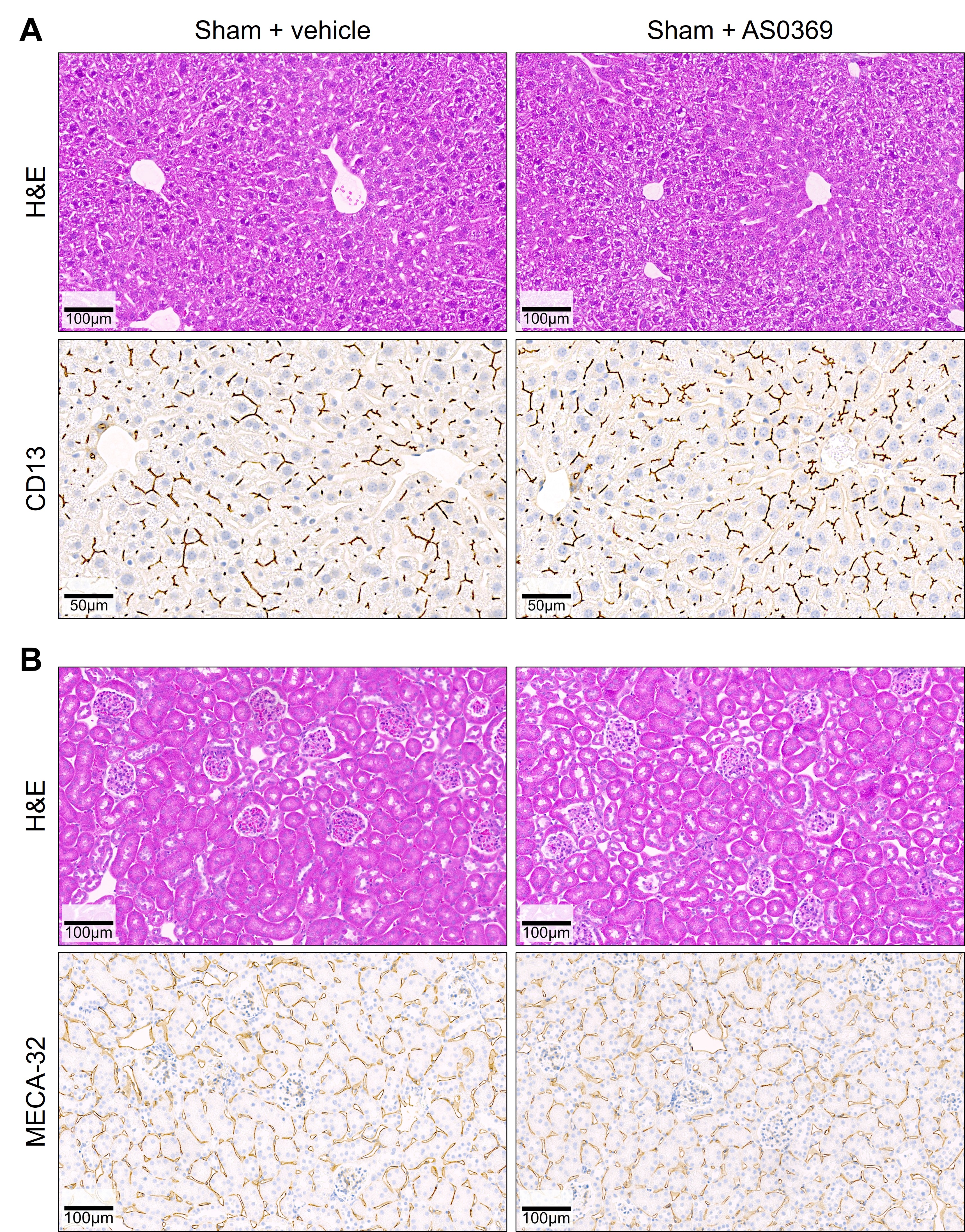


# **Supplementary Tables**

Suppl. Table 1. Antibodies used for immunohistochemistry.

| **Target** | **Primary antibodies** | | **Secondary antibodies** | |
| --- | --- | --- | --- | --- |
|  | **Antibody** | **Dilution** | **Antibody** | **Dilution** |
| Cholangiocyte | Anti-cytokeratin 19 antibody, rabbit | 1:500 | Ultra-Map anti rabbit HRP | Automatic Discovery Ready to use |
| Bile canaliculi | Recombinant anti-CD13 antibody, rabbit | 1:16000 | Ultra-Map anti rabbit HRP |  |
| Leukocytes | Anti-CD45 antibody, rat | 1:400 | Omni-Map anti rat HRP |  |
| Endothelial cells | Anti-MECA-32 antibody, rat | 1:250 | Ultra-Map anti rat HRP |  |
| ASBT | Anti-ASBT antibody, goat | 1:500 | Ultra-Map anti goat HRP |  |
| Proliferating cells | Anti-Ki-67 antibody, rabbit | 1:100 | Ultra-Map anti rabbit HRP |  |
| NTCP | Anti-NTCP antibody, rabbit | 1:3000 | Ultra-Map anti rabbit HRP |  |

Suppl. Table 2. TaqMan gene assays.

| **Gene** | **TaqMan Assay ID** |
| --- | --- |
| *Abcb11* | Mm00445168_m1 |
| *Abcc2* | Mm00496899_m1 |
| *Abcc3* | Mm00551550_m1 |
| *Abcc4* | Mm01226381_m1 |
| *Egr1* | Mm00656724_m1 |
| *GAPDH* | 4352932E |
| *Slc1a2* | Mm01275814_m1 |
| *Slc10a1* | Mm00441421_m1 |
| *Slc10a2* | Mm00488258_m1 |
| *Slc51a* | Mm00521530_m1 |
| *Slc22a8* | Mm00459534_m1 |

Suppl. Table 3: Bioinformatics of the kidney: Gene movements from day 3 to day 63


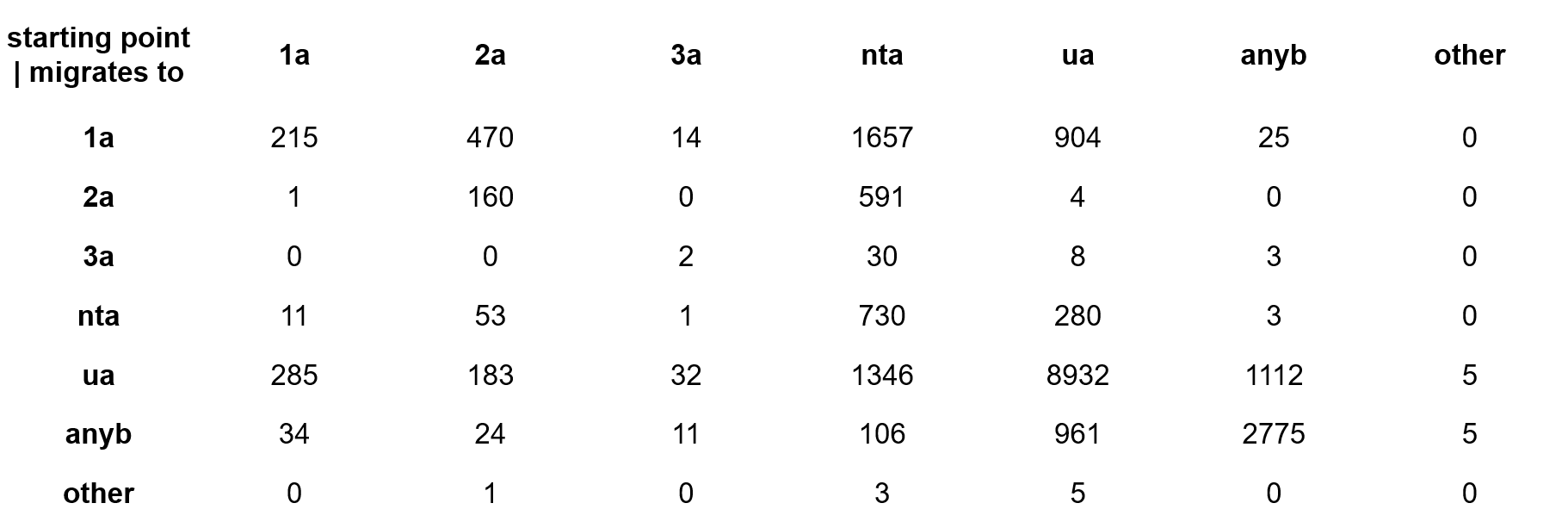

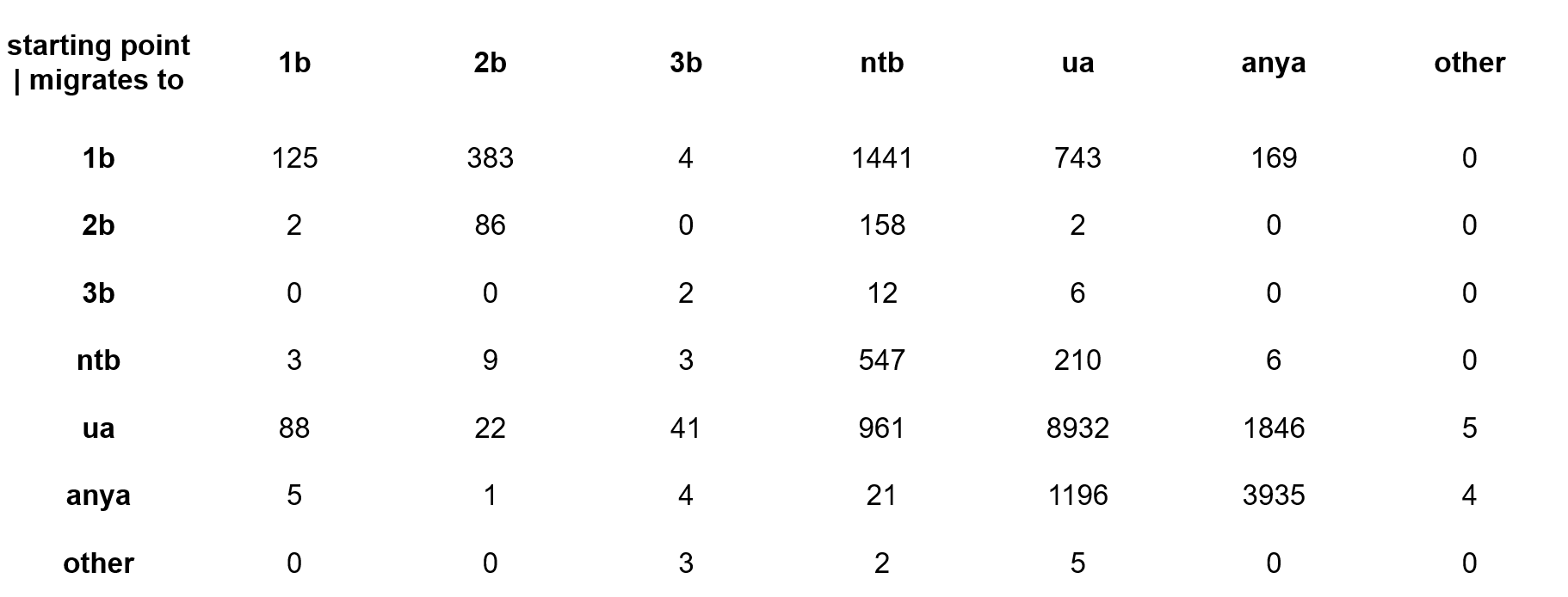


Suppl. Table 4: Bioinformatics of the liver: Gene movements from day 3 to day 63


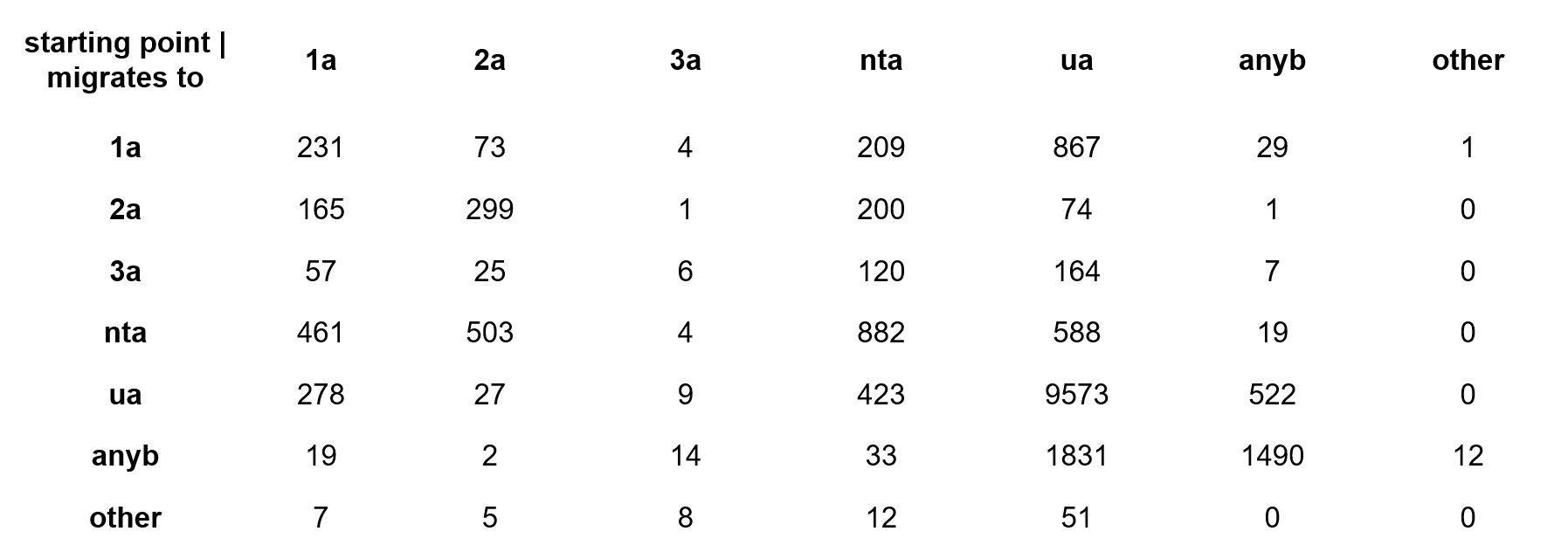

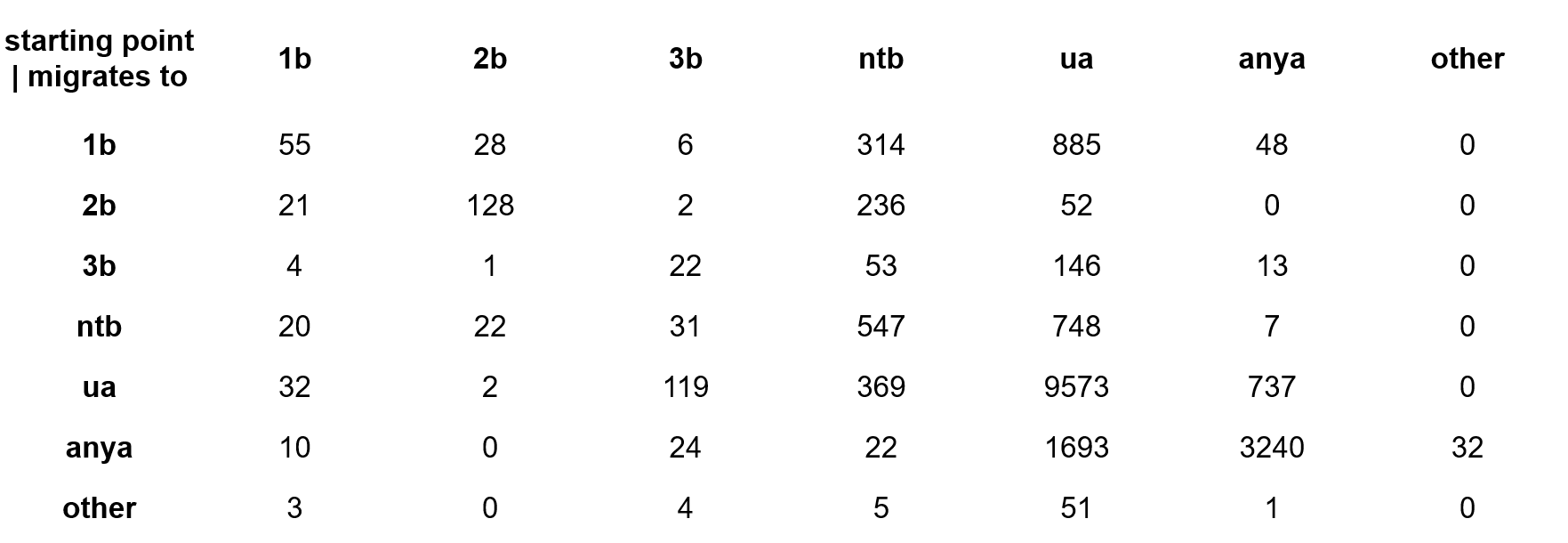


# **Supplementary references**

**Author names in bold designate shared co-first authorship.**

[1] Ghallab A, González D, Strängberg E, et al. Inhibition of the renal apical sodium dependent bile acid transporter prevents cholemic nephropathy in mice with obstructive cholestasis. J Hepatol 2024;80:268-281.

[2] Ghallab A, Hofmann U, Sezgin S, et al. Bile Microinfarcts in Cholestasis Are Initiated by Rupture of the Apical Hepatocyte Membrane and Cause Shunting of Bile to Sinusoidal Blood. Hepatology 2019;69:666-683.

[3] Ghallab A, Hassan R, Hofmann U, et al. Interruption of bile acid uptake by hepatocytes after acetaminophen overdose ameliorates hepatotoxicity. J Hepatol 2022;77:71-83.

[4] **Custodio RJP, Hobloss Z, Myllys M,** et al. Cognitive Functions, Neurotransmitter Alterations, and Hippocampal Microstructural Changes in Mice Caused by Feeding on Western Diet. Cells 2023;12.

[5] Holland CH, Ramirez Flores RO, Myllys M, et al. Transcriptomic Cross-Species Analysis of Chronic Liver Disease Reveals Consistent Regulation Between Humans and Mice. Hepatol Commun 2022;6:161-177.

[6] Hall MJ. A staining reaction for bilirubin in sections of tissue. Am J Clin Pathol 1960;34:313-316.

[7] Bankhead P, Loughrey MB, Fernández JA, et al. QuPath: Open source software for digital pathology image analysis. Sci Rep 2017;7:16878.

[8] Guttman A. R-trees: a dynamic index structure for spatial searching. ACM SIGMOD Conference; 1984; 1984.

[9] LeCun Y, Bengio Y, Hinton G. Deep learning. Nature 2015;521:436-444.

[10] Ronneberger O, Fischer P, Brox T. U-Net: Convolutional Networks for Biomedical Image Segmentation. In: Navab N, Hornegger J, Wells WM, Frangi AF, editors. Medical Image Computing and Computer-Assisted Intervention – MICCAI 2015; 2015 2015//; Cham: Springer International Publishing; 2015. p. 234-241.

[11] Isensee F, Jaeger PF, Kohl SAA, et al. nnU-Net: a self-configuring method for deep learning-based biomedical image segmentation. Nat Methods 2021;18:203-211.

[12] Schmidt U, Weigert M, Broaddus C, et al. Cell Detection with Star-Convex Polygons. In: Frangi AF, Schnabel JA, Davatzikos C, Alberola-López C, Fichtinger G, editors. Medical Image Computing and Computer Assisted Intervention – MICCAI 2018; 2018 2018//; Cham: Springer International Publishing; 2018. p. 265-273.

[13] Lee T-C, Kashyap RL, Chu CN. Building Skeleton Models via 3-D Medial Surface/Axis Thinning Algorithms. CVGIP Graph Model Image Process 1994;56:462-478.

[14] Patro R, Duggal G, Love MI, et al. Salmon provides fast and bias-aware quantification of transcript expression. Nature Methods 2017;14:417-419.

[15] Team RC. R: A language and environment for statistical computing. R Foundation for Statistical Computing, Vienna, Austria <https://wwwR-projectorg/> 2024.

[16] Love MI, Soneson C, Hickey PF, et al. Tximeta: Reference sequence checksums for provenance identification in RNA-seq. PLoS Comput Biol 2020;16:e1007664.

[17] Love MI, Huber W, Anders S. Moderated estimation of fold change and dispersion for RNA-seq data with DESeq2. Genome Biology 2014;15:550.

[18] Zhu A, Ibrahim JG, Love MI. Heavy-tailed prior distributions for sequence count data: removing the noise and preserving large differences. Bioinformatics 2019;35:2084-2092.

[19] Alexa A, Rahnenführer J, Lengauer T. Improved scoring of functional groups from gene expression data by decorrelating GO graph structure. Bioinformatics 2006;22:1600-1607.

[20] **Nell P, Kattler K, Feuerborn D**, et al. Identification of an FXR-modulated liver-intestine hybrid state in iPSC-derived hepatocyte-like cells. J Hepatol 2022;77:1386-1398.
