# Supplementary material for: Stage-dependent effects of systemic ASBT inhibition in a cholestasis-induced cholemic nephropathy mouse model
Source: JHEP Rep. 2025 Sep 23;7(12):101599. doi: 10.1016/j.jhepr.2025.101599 (PMC12657750; doi:10.1016/j.jhepr.2025.101599)
Supplement: Multimedia component 2 [file mmc2.docx]

**Journal of Hepatology**

**CTAT methods**

Tables for a “Complete, Transparent, Accurate and Timely account” (CTAT) are now mandatory for all revised submissions. The aim is to enhance the reproducibility of methods.

- Only include the parts relevant to your study
- Refer to the CTAT in the main text as ‘Supplementary CTAT Table’
- Do not add subheadings
- Add as many rows as needed to include all information
- Only include one item per row

**If the CTAT form is not relevant to your study, please outline the reasons why:**

|  |
| --- |

- 1. **Antibodies**

| **Name** | **Citation** | **Supplier** | **Cat no.** | **Clone no.** |
| --- | --- | --- | --- | --- |
| Recombinant anti-cytokeratin 19 antibody | https://www.abcam.com/en-us/products/primary-antibodies/cytokeratin-19-antibody-ep1580y-cytoskeleton-marker-ab52625 | Abcam | ab52625 |  |
| Purified rat anti-mouse CD45 | https://www.bdbiosciences.com/en-de/products/reagents/microscopy-imaging-reagents/immunohistochemistry-reagents/purified-rat-anti-mouse-cd45.550539?tab=product_details | BD-Pharmingen | 550539 |  |
| Ki-67 (D3B5) Rabbit mAb (IHC Formulated) | https://www.cellsignal.com/products/primary-antibodies/ki-67-d3b5-rabbit-mab-ihc-formulated/12202 | Cell Signaling Technology | 12202S |  |
| Anti-MECA-32 | https://dshb.biology.uiowa.edu/MECA-32 | DSHB | AB_531797 |  |
| SLC10A2 polyclonal antibody/ASBT | https://www.thermofisher.com/antibody/product/SLC10A2-Antibody-Polyclonal/PA5-18990 | Invitrogen | PA5-18990 |  |
| NTCP Polyclonal Antibody | https://www.thermofisher.com/antibody/product/NTCP-Antibody-Polyclonal/PA5-80001 | Invitrogen | PA5-8001 |  |
| Ultra-Map anti rabbit HRP | https://shop.roche-diagnostics.ch/labor/05269717001 | Roche | 05 269 717 001 |  |
| Ultra-Map anti rabbit Alk Phos | https://shop.roche-diagnostics.ch/labor/05269709001 | Roche | 05 269 709 001 |  |
| Recombinant anti-CD13 antibody, rabbit | https://www.abcam.com/products/primary-antibodies/cd13-antibody-epr4058-ab108310.html | Abcam | ab108310 |  |
| UltraMap anti-goat multimer | https://shop.roche-diagnostics.ch/laboratoire/06607241001 | Roche | 06 607 241 001 |  |
| OmniMap anti-rat HRP | https://shop.roche-diagnostics.ch/labor/05891892001 | Roche | 760-4457 |  |

- 1. **Cell lines**

| **Name** | **Citation** | **Supplier** | **Cat no.** | **Passage no.** | **Authentication test method** |
| --- | --- | --- | --- | --- | --- |
|  |  |  |  |  |  |

- 1. **Organisms**

| **Name** | **Citation** | **Supplier** | **Strain** | **Sex** | **Age** | **Overall n number** |
| --- | --- | --- | --- | --- | --- | --- |
| Mouse | https://janvier-labs.com/en/fiche_produit/c57bl-6n_mouse/ | Janvier Labs | C57BL/6N | Male | 8-10 weeks | 92 |

- 1. **Sequence based reagents**

| **Name** | **Sequence** | **Supplier** |
| --- | --- | --- |
| Abcb11 (Mm00445168_m1) | - | Thermo Fisher Scientific |
| Abcc2 (Mm00496899_m1) | - | Thermo Fisher Scientific |
| Abcc3 (Mm00551550_m1) | - | Thermo Fisher Scientific |
| Abcc4 (Mm01226381_m1) | - | Thermo Fisher Scientific |
| Cyp7a1 (Mm00484150_m1) | - | Thermo Fisher Scientific |
| Egr1 (Mm00656724_m1) | - | Thermo Fisher Scientific |
| GAPDH (Mm99999915_g1) | - | Thermo Fisher Scientific |
| Slc22a8 (Mm00459534_m1) |  | Thermo Fisher Scientific |
| Slc10a1 (Mm00441421_m1) | - | Thermo Fisher Scientific |
| Slc10a2 (Mm00488258_m1) | - | Thermo Fisher Scientific |
| Slco1b2 (Mm00451510_m1) | - | Thermo Fisher Scientific |
| Slc51a (Mm00521530_m1) | - | Thermo Fisher Scientific |

- 1. **Biological samples**

| **Description** | **Source** | **Identifier** |
| --- | --- | --- |
| Kidney | Mouse | - |
| Plasma | Mouse | - |
| Urine | Mouse | - |
| Liver | Mouse | - |
| Bile | Mouse | - |

- 1. **Deposited data**

| **Name of repository** | **Identifier** | **Link** |
| --- | --- | --- |
| Sequence Read Archive (SRA) | https://www.ncbi.nlm.nih.gov/sra/ | <https://www.ncbi.nlm.nih.gov/sra/PRJNA1224581> |

- 1. **Software**

| **Software name** | **Manufacturer** | **Version** |
| --- | --- | --- |
| GraphPad Prism 10 Software | GraphPad | 10.4.0 |
| Zen | Carl-Zeiss |  |
| QuestTM | Thermo Scientific |  |
| QuPath v0.5.1 | Peter Bankhead | 0.5.1 |
| R | R Development Core Team | 4.4.1 |
| Salmon | Salmon Software | 1.5.0 |
| Scils Lab MVS | Bruker Daltonics | 2024b Pro |
| Skyline | University of Washington | 24.1 |

- 1. **Other (e.g. drugs, proteins, vectors etc.)**

| AS0369 | Albireo |  |
| --- | --- | --- |
| Hoechst 33258 | Thermo Fisher Scientific | H21491 |
| Tetramethylrhodamin-Ethylester (TMRE) | Thermo Fisher Scientific | T669 |
| Cholyl-lysyl-fluorescein (CLF) | Corning, USA | 451041 |
| Discovery inhibitor (H2O2 blocker) | Roche | 7017944001 |
| Eosin Y | Sigma-Aldrich | E4382-25g |
| Hematoxylin II | Roche | 05 277 965 001 |
| Mouse Lipocalin-2/NGAL DuoSet ELISA | R&D Systems | DY1857 |
| DuoSet ELISA Ancillary Reagent Kit 2 | R&D Systems | DY008 |
| Mouse KIM 1 ELISA Kit | Abcam | ab213477 |
| RNeasy Mini Kit | Qiagen | 74106 |
| TaqMan universal mastermix with UNG | Thermo Fisher Scientific | 4426710 |
| High-Capacity cDNA Reverse Transcription Kit | Thermo Fisher Scientific | 4368814 |
| Bile Acid Standard 1 Mix (unconjugated) | Cambridge Isotope Laboratories, Inc. | MSK-BA1-1 |
| Bile Acid Standard 1 Mix (unconjugated) unlabelled standard | Cambridge Isotope Laboratories, Inc. | MSK-BA1-US-1 |
| Bile Acid Standard 2 Mix (conjugated) | Cambridge Isotope Laboratories, Inc. | MSK-BA2-1 |
| Bile Acid Standard 2 Mix (conjugated) unlabelled standard | Cambridge Isotope Laboratories, Inc. | MSK-BA2-US-1 |
| 7α-Hydroxy-4-cholesten-3-one (C4) | Sigma-Aldrich | 52853-1MG |
| Cholic acid sulfate (CA-S) | IsoSciences LLC | 13098UNL3SO |
| Taurocholic acid sulfate, sodium salt (TCA-S) | IsoSciences LLC | 13232UNL3SO |
| α-Tauro-muricholate, sodium salt (αTMCA) | Toronto Research Chemicals | T009130 |
| β-Tauro-muricholate, sodium salt (βTMCA) | Toronto Research Chemicals | T009135 |
| α-Muricholate (αMCA) | Toronto Research Chemicals | M732750 |
| ω-Muricholate (ωMCA) | Toronto Research Chemicals | M732760 |
| d4- Cholic acid sulfate (d4-CA-S) | IsoSciences LLC | 130983SO |
| d4- Taurocholic acid sulfate, sodium salt (d4-TCA-S) | IsoSciences LLC | 132323SO |
| d4-α-Tauro-muricholate, sodium salt (d4-αTMCA) | Toronto Research Chemicals | T009132 |
| d5-α-Muricholate (d5-αMCA) | Toronto Research Chemicals | M732752 |
| Piccolo General Chemistry 13 | Hitado | AB-114-400-0029 |
| Picrosirius red stain kit | Polyscience, Inc. | 249410-250 |
| Bluing Reagent | Roche | 05 266 769 001 |
| Bile Stain Kit-Halls | StatLab | KTHBI |
| Discovery Purple Kit RUO | Roche | 07 053 983 001 |
| Chromo Map DAB | Roche | 05 266 645 001 |
| Picrosirius red stain kit | Polyscience, Inc. | 249410-250 |
| RNA 6000 Nano Kit | Agilent Technologies | 5067-1511 |
| DNA 1000 Kit | Agilent Technologies | 5067-1504 |
| Qubit™ RNA BR Assay Kit | Thermo Fisher Scientific | Q10210 |
| Qubit™ 1X dsDNA HS Assay Kit | Thermo Fisher Scientific | Q33230 |
| TruSeq® Stranded mRNA Library Prep | Illumina | 20020595 |
| IDT for Illumina TruSeq RNA UD Indices | Illumina | 20022371 |
| Agencourt AMPure XP 60 ml kit | Beckman Coulter Genomics | A63881 |
| SuperScript II Reverse Transcriptase | Thermo Fisher Scientific | 18064014 |
| NextSeq 500/550 High Output Kit v2.5 (150 Cycles) | Illumina | 20024907 |
| 2-Mercaptobenzothiazole [matrix] | Sigma Aldrich | 81467.0250 |

- 1. **Please provide the details of the corresponding methods author for the manuscript:**

| Prof. Dr. Ahmed Ghallab: E-mail: ghallab@ifado.de; Phone: +492311084356)  Prof. Dr. Jan G. Hengstler: E-mail: hengstler@ifado.de; Phone: +492311084348 |
| --- |

**2.0 Please confirm for randomised controlled trials all versions of the clinical protocol are included in the submission. These will be published online as supplementary information.**

| Not Applicable |
| --- |
