# Supplementary material for: Stage-dependent effects of systemic ASBT inhibition in a cholestasis-induced cholemic nephropathy mouse model
Source: JHEP Rep. 2025 Sep 23;7(12):101599. doi: 10.1016/j.jhepr.2025.101599 (PMC12657750; doi:10.1016/j.jhepr.2025.101599)
Supplement: Multimedia component 6 [file mmc6.pdf]

Stage-dependent effects of systemic ASBT inhibition in a cholestasis-induced cholemic nephropathy mouse model

Authors

Ahmed Ghallab, Maiju Myllys, Daniela González, …, Paul A. Dawson, Erik Lindström, Jan G. Hengstler

Correspondence

ghallab@ifado.de (A. Ghallab), hengstler@ifado.de (J.G. Hengstler).

Graphical abstract

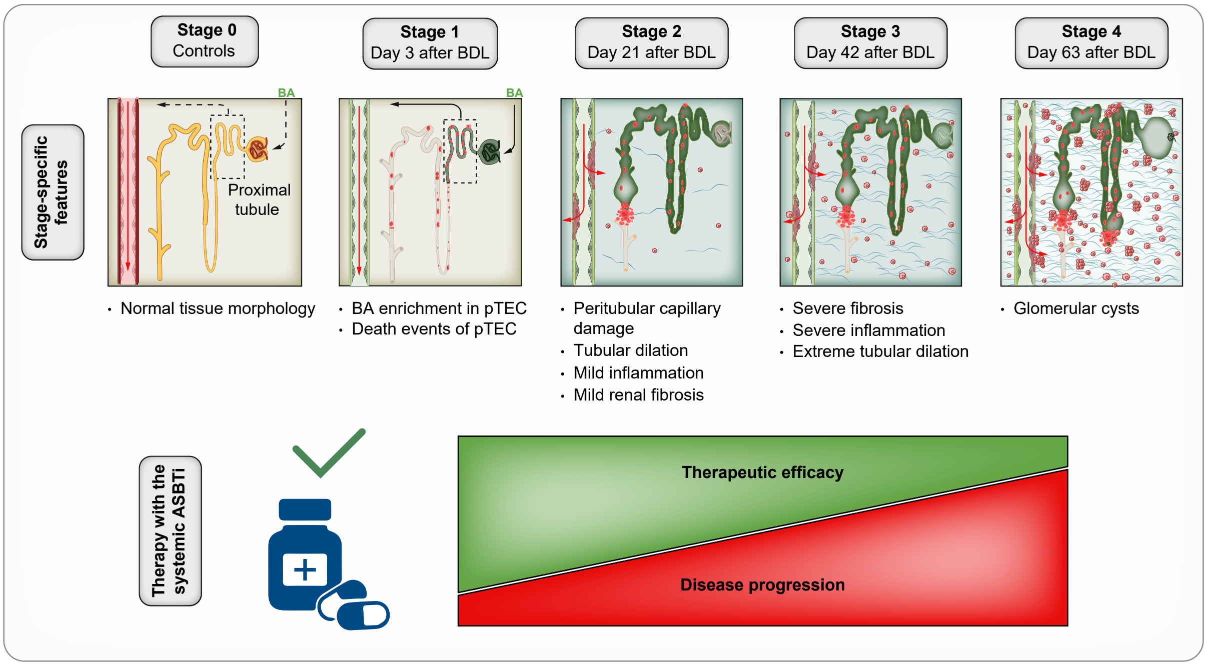

Highlights:

- The therapeutic effect of systemic ASBT inhibition in mice with cholemic nephropathy is disease stage dependent.
- The therapeutic effect is smaller in advanced compared to early stages but is still statistically significant.
- ASBT inhibition increases urinary excretion of bile acids and reduces bile acid levels in the kidney and blood.
- ASBT inhibition improves histological hallmarks of cholemic nephropathy, even in advanced stages.
- ASBT inhibition in normal mice has only minor effects on the liver and kidneys.

Impact and implications:

This study demonstrates that systemic inhibition of the apical sodium-dependent bile acid transporter (ASBTi) alleviates cholemic nephropathy across disease stages in a bile duct ligation mouse model. The greatest benefit was achieved when treatment was initiated early, coinciding with proximal tubular epithelial cell death, but even advanced features such as glomerular cysts were partially reversed. These findings highlight ASBTi as a promising therapeutic strategy for cholemic nephropathy, addressing a major unmet need in cholestatic liver disease. By targeting bile acid accumulation and related injury pathways, ASBTi may improve renal outcomes and broaden treatment options in affected patients.

https://doi.org/10.1016/j.jhepr.2025.101599
© 2025 The Author(s). Published by Elsevier B.V. on behalf of European Association for the Study of the Liver (EASL). This is an open access article under the CC BY license (http://creativecommons.org/licenses/by/4.0/). JHEP Reports, 2025, 7, 1–13

JHEP Reports

Stage-dependent effects of systemic ASBT inhibition in a cholestasis-induced cholemic nephropathy mouse model☆

Ahmed Ghallab[1,2,*], Maiju Myllys[1], Daniela González[1], Adrian Friebel[3], Zaynab Hobloss[1], Reham Hassan[1,2], Hannah Schmidt[1], Qasim Siddiqui[3], Deng Zhipeng[3], Rama Hendawi[1], Brigitte Begher-Tibbe[1], Joerg Reinders[1], Katharina Derksen[1], Ute Hofmann[4], Julia C. Duda[5], Lucia Ameis[6], Kathrin Möllenhoff[6], Abdellatief Seddek[2], Noha Abdelmageed[7], Ellen Strängberg[8], Peter Åkerblad[8], Mihael Vucur[9], Tom Luedde[9], Guido Stirnimann[10], Matthias Schwab[4,11], Tahany Abbas[12], Benedikt Hild[13], Hartmut Schmidt[13], Saul J. Karpen[14], Benedikt Simbrunner[15], Mattias Mandorfer[15], Jörg Rahnenführer[5], Karolina Edlund[1], Stefan Hoehme[3], Michael Trauner[15], Paul A. Dawson[16], Erik Lindström[8], Jan G. Hengstler[1,*]

JHEP Reports **2025**. vol. 7 | 1–13

Background & Aims: Cholemic nephropathy (CN) is a severe complication of liver diseases associated with cholestasis and represents an unmet medical need. Recently, we identified the molecular mechanism of CN and showed that the systemic apical sodium-dependent bile acid transporter inhibitor (ASBTi) AS0369 prevented CN in mice. However, it is not clear if ASBTi is effective in a therapeutic rather than a preventive setting.

Methods: AS0369 was administered daily for 4 weeks to bile duct-ligated (BDL) mice at four CN stages: (1) early stage with proximal tubular epithelial cell (pTEC) death (BDL-day 3); (2) inflammation, leaky peritubular capillaries, and tubular dilatation (BDL-day 21); (3) fibrosis (BDL-day 42); and (4) advanced stage with glomerular cysts (BDL-day 63). Disease progression was evaluated by biochemical, histopathological, and RNA-sequencing analysis.

Results: ASBTi increased urinary excretion of bile acids (BAs) and reciprocally reduced BA concentrations in blood and renal tissue at all disease stages. Therapeutic efficacy was highest when ASBTi was given at early disease stages, *e.g.* urinary BA excretion was increased 9-fold (p <0.001) at the early stage compared to 4-fold (p = 0.021) at the late stage. ASBTi reduced the pTEC injury biomarker KIM-1, tissue damage, replacement proliferation, peritubular capillary damage and renal fibrosis. Additionally, late-stage disease features, such as glomerular cysts, were ameliorated (46% at the late stage, p = 0.005) by the ASBTi. RNA-sequencing revealed that ASBTi attenuated BDL-induced gene deregulation at all stages, with a larger effect size at early stages.

Conclusions: Early systemic ASBTi therapy, initiated at the onset of pTEC death, provides the greatest therapeutic benefit. Nonetheless, even at later stages, ASBTi can ameliorate features of advanced CN.

© 2025 The Author(s). Published by Elsevier B.V. on behalf of European Association for the Study of the Liver (EASL). This is an open access article under the CC BY license (http://creativecommons.org/licenses/by/4.0/).

Introduction

Cholemic nephropathy (CN) is an acute renal dysfunction, associated with high morbidity and mortality, that occurs in the context of liver diseases associated with cholestasis, including acute liver failure, alcohol-related hepatitis and decompensated cirrhosis with acute-on-chronic liver failure, as well as obstructive jaundice.[1–5] In preclinical studies of CN, bile duct ligated (BDL) mice are commonly used, because the animals develop several features observed in patients with CN, such as tubular epithelial cell damage, tubular casts, cystic dilatation of renal tubules, fibrosis, and compromised renal function.[1,6,7] Recently, the spatio-temporal sequence of events leading to CN was studied in BDL mice using intravital microscopy.[7] Already within the first hours after BDL, bile acids (BA) strongly increase in blood leading to enhanced glomerular filtration and increased BA concentrations in renal tubules. Consequently, proximal tubular epithelial cells (pTECs) enrich BA due to the activity of the a*pical* sodium-dependent bile acid transporter (ASBT; SLC10A2) leading to oxidative stress and cell death. Dying pTECs release cell debris, which floats downstream and forms obstructions in distal tubules and collecting ducts leading to dilatations upstream of the casts. Concurrently, pTECs enrich BA at the interstitial side via the activities of OSTα/β (SLC51A/SLC51B) and MRP3 (ABCC3), which leads to leaky peritubular capillaries. Next, fibrosis is triggered and – as a late event – glomerular cysts are formed.

☆ Given their role as Associate Editors, Mattias Mandorfer had no involvement in the peer-review of this article and had no access to information regarding its peer-review. Full responsibility for the editorial process for this article was delegated to the Co-Editor Sophie Lotersztajn and Editor-in-Chief Josep M Llovet.

* Corresponding authors. Address: Department of Toxicology, Leibniz Research Centre for Working Environment and Human Factors, Technical University Dortmund, Ardeystr. 67, 44139, Dortmund, Germany; Tel. +492311084356 (A. Ghallab), or +492311084348 (J.G. Hengstler).
E-mail addresses: ghallab@ifado.de (A. Ghallab), hengstler@ifado.de (J.G. Hengstler).

https://doi.org/10.1016/j.jhepr.2025.101599

An early key event initiating the above-described hallmarks of CN is ASBT-mediated BA enrichment in pTECs.[7] Blocking BA enrichment in pTECs via inhibition of renal ASBT has been shown to prevent cell death events and further adverse effects of CN. Recently, a systemically bioavailable ASBT inhibitor (ASBTi) AS0369 was developed.[7] AS0369 potently inhibits the mouse ASBT with a half-maximal inhibitory concentration (IC_{50}) of 1.31 nM, shows a more than 100-fold higher affinity for mouse ASBT compared to mouse NTCP (sodium-taurocholate co-transporting polypeptide; SLC10A1), and has appropriate pharmacokinetics after oral administration so that inhibitory concentrations can be reliably maintained by two oral doses of 60 mg/kg a day.

A key limitation of the above-described study was that systemic ASBT inhibition was initiated on the same day as BDL was performed.[7] Using this experimental design, it was demonstrated that systemic ASBT inhibition prevented CN when applied at the same time as the intervention that causes cholestasis. It is not yet known whether systemic ASBTi can also be used for therapy, and if their efficacy depends on the stage of the disease. Notably, patients with the above-mentioned conditions often present when acute kidney injury (AKI) has already occurred. Moreover, even in those without AKI at presentation, the need to apply ASBTi preventively would result in a fundamentally different approach to clinical development compared to only treating the subset of patients with evident kidney injury.

In the present study, we investigated the therapeutic efficacy of ASBTi in mice representing four increasingly advanced stages of CN. We report that while early ASBTi application in CN is essential for maximum benefit, treatment with the systemic ASBTi AS0369 yielded a clear improvement in renal histopathology and gene expression changes, even when given at the most advanced CN stage.

Materials and methods

A detailed description of the materials and methods is provided in the supplementary data and CTAT table.

Induction of obstructive cholestasis in mice and administration of the systemic ASBTi

Eight-to-ten-week-old male C57BL/6N mice (Janvier Labs, France) were used. The mice were housed at standard environmental conditions with free access to water, and *ad libitum* feeding on a standard rodent diet (Ssniff, Soest, Germany). All experiments were ethically approved by the local committee (LANUV, North Rhine-Westphalia, Germany, application number: 81-02.04.2022.A286). To induce obstructive cholestasis, the extrahepatic common bile duct was ligated at a position between the gallbladder and the duodenum, as previously described.[7] Sham control mice underwent the same operative procedure but without BDL. The BDL mice received AS0369 (60 mg/kg) or vehicle (0.5% methyl cellulose and 0.1 % tween 80) orally by gavage twice per day for 4 weeks starting on either day 3, 21, 42, or 63 after the surgery.

BA analysis

Concentrations of BA in liver and kidney tissues were determined by negative electrospray liquid chromatography-tandem mass spectrometry in MRM (multiple-reaction-monitoring) mode on an Agilent 6495B triple quadrupole mass spectrometer (Agilent, Germany) coupled to an Agilent Infinity II HPLC system. BA analysis in blood plasma and urine was accomplished by liquid chromatography-mass spectrometry (supplementary methods).

Results

Stage-dependent improvement of kidney injury markers with systemic ASBTi therapy

To study the stage-dependent therapeutic efficacy of systemic ASBT inhibition we chose the following disease stages after BDL: (1) day 3, when pTEC death occurs but peritubular capillaries, renal tubules, and glomeruli are intact (Fig. S1A and B); (2) day 21, when peritubular capillaries are compromised as evidenced by reduced MECA-32 immunostaining (Fig. S1A) and capillary leakage in intravital imaging (Fig. S1B) – moreover, tubular dilatation, leukocyte infiltration, but only very mild renal fibrosis are observed (Fig. S1A); (3) day 42, with severe fibrosis and extreme tubular dilatation (Fig. S1A); (4) day 63, when glomerular cysts occur, and all other above-mentioned disease characteristics become more severe (Fig. S1A).

An interesting difference in the adaptation of the liver and kidney to cholestasis was observed with respect to BA uptake transporters (Fig. S2A). Protein levels of NTCP, the major BA uptake transporter in hepatocytes, were strongly downregulated on day 3 after BDL and thereafter (Fig. S2A and B). In contrast, ASBT, the major BA uptake transporter in renal pTECs was unaffected or only moderately downregulated (Fig. S2A and C). Thus, the liver and kidney show marked differences in their ability to adapt to cholestasis.

To study the influence of the stage of CN on therapeutic efficacy, we used a 4-week treatment period with the systemic ASBTi AS0369, starting 3 (stage 1), 21 (stage 2), 42 (stage 3), or 63 (stage 4) days after BDL (Fig. 1A). BDL caused a macroscopically visible greenish discoloration of the kidneys, which was ameliorated by AS0369 therapy in all disease stages (Fig. 1B). BDL massively increased the size of the gallbladder and bile volume, which was reduced by AS0369 (Fig. 1B,C). This macroscopic observation was evident when quantified as the bile volume to body weight ratio, and the AS0369 effect size was more pronounced when initiated at the earlier stages (Fig. 1B,C). Total bilirubin concentrations in the blood were significantly decreased by the AS0369 therapy at all disease stages (Fig. 1D). KIM-1, a biomarker of pTEC injury, is known to increase only in the first days after BDL.[7] Consistent with previous results, KIM-1 strongly increased in blood at the early stage after BDL, followed by lower levels in the later stages (Fig. 1D). This early (day 3) increase was completely suppressed by AS0369 (Fig. 1D). Neutrophil gelatinase-associated lipocalin (NGAL) is an injury marker of all tubular epithelial cells and increased in blood to a similar extent at all periods after BDL (Fig. 1D). AS0369 significantly reduced NGAL levels when therapy started on days 3 and 21 but the reduction in NGAL levels did not reach statistical significance at the later stages (Fig. 1D). BDL caused a loss of body weight that was ameliorated by AS0369 if initiated up to stage 3, but not at stage 4, where no significant effect was obtained (Fig. S3A). Approximately 20–30% of BDL mice either died or exceeded our health score criteria, requiring euthanasia. Treatment with

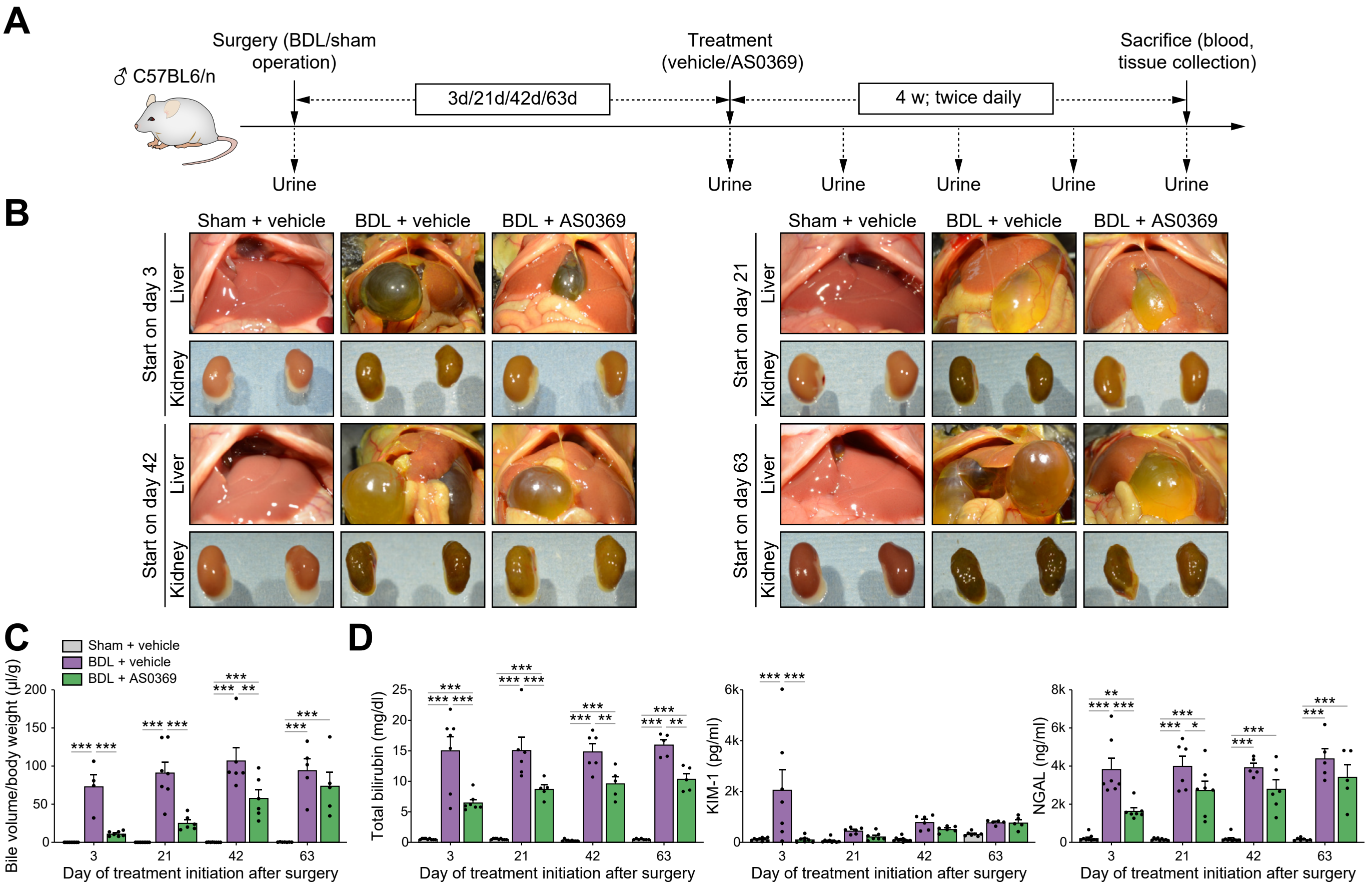

Fig. 1. Therapeutic efficacy of AS0369 in four disease stages. (A) Experimental design; 60 mg/kg AS0369 or vehicle were given twice daily by gavage for 4 weeks. (B) Macroscopic appearance of the livers and kidneys; (C) Ratio of bile volume to body weight; (D) Concentrations of total bilirubin KIM-1 and NGAL in the blood. **p* <0.05; ***p* <0.01; ****p* <0.001; Tukey's multiple comparisons test. Data are presented as mean± SEM. n = 4-9 mice per group; the dots in the bars represent individual mice. BDL: bile duct ligation; KIM-1: kidney injury molecule1; NGAL: neutrophil gelatinase-associated lipocalin.

AS0369 improved survival (defined by death or exceedance of the score sheet criteria) for the groups treated at stages 1 to 3 after BDL (Fig. S3B). If therapy was initiated at stage 4, the percentage of surviving mice during the 4-week treatment period was higher, which may reflect adaptation to the cholestatic situation for mice that have survived the longer BDL periods (*i.e.* survivorship bias).

Target protection and reduced systemic BA load by systemic ASBT inhibition in all disease stages

Therapeutic effects of the systemic ASBTi can be achieved via two mechanisms: by reduced uptake of BA into the target cells (pTECs) of the kidney (target protection), and by reduced systemic BA load because of increased urinary BA excretion (systemic effect). AS0369 caused a marked increase in urinary BAs and a corresponding decrease in blood BA at all four disease stages, with a smaller effect when therapy was initiated later after BDL (Fig. 2A,B). Total BAs detected in kidney tissue homogenate were significantly reduced by AS0369 when treatment began up to stage 3, indicating efficient target protection (Fig. 2C). The results obtained by mass spectrometry of tissue homogenate were confirmed by matrix assisted laser desorption/ionization mass spectrometry imaging of tissue sections, demonstrating that AS0369 strongly reduced the TCA signal in the kidney tissue of BDL mice at all disease stages (Figs 2D,E and S4). Also in liver tissue, AS0369 significantly reduced levels of BAs in BDL mice in the two earliest (days 3 and 21) but not the later (days 42 and 63) disease stages (Figs 2C-E and S5). The changes in BA concentrations in kidney tissues following AS0369 treatment were associated with changes in BA transporter expression (Fig. S6). AS0369 ameliorated the influence of BDL on *Asbt, Oat3, Mrp3* and *Mrp4* RNA levels in the kidney at early disease stages, with the effect size decreasing in later stages (Fig. S6). No significant alterations in *Ost-α* and *Mrp2* expression were observed in the kidney tissues following AS0369 treatment (Fig. S6). In contrast to the kidney, BDL-induced alterations of the hepatic BA transporters remained mainly unaffected by the ASBTi (Fig. S7).

Stage-dependent improvement of renal tissue injury by systemic ASBT inhibition

A relatively early feature of CN is the occurrence of damaged tissue and dilated renal tubules, which occur 3-4 weeks after BDL.[7] H&E-stained kidney tissue revealed that AS0369 therapy ameliorates renal damage, including tubular dilation, across all disease stages (Fig. 3A). To quantify the extent of damaged renal tissue in whole-organ sections, the intensity of the eosin

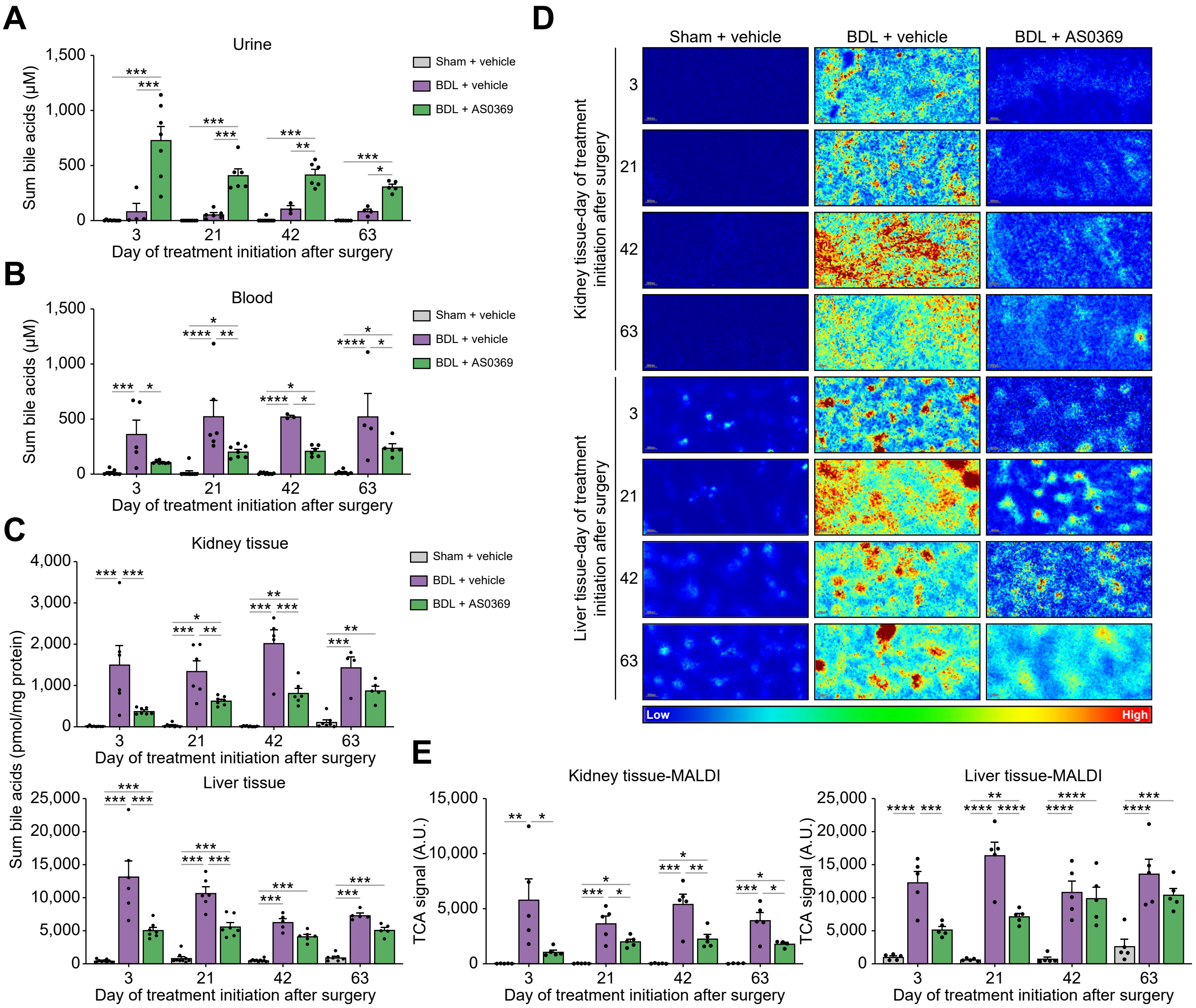

Fig. 2. Influence of AS0369 on BA concentrations. (A-C) Sum BA in urine, blood plasma, and kidney as well as liver tissue homogenates. (D) Tissue TCA levels analyzed by MALDI-MSI in frozen kidney and liver tissues. (E) Quantifications of TCA MALDI-MSI signals in kidney and the liver tissues. *p <0.05; **p <0.01; ***p <0.001; ****p <0.0001; Tukey's multiple comparisons test. Data are presented as mean± SEM. n = 3-9 mice per group; the dots in the bars represent individual mice. BA, bile acid; BDL: bile duct ligation; MALDI-MSI, matrix assisted laser desorption/ionization mass spectrometry imaging; TCA, taurocholic acid; TCMA, tauro-muricholic acid.

staining can be used,[7] since features of tissue damage, such as dilated tubules, fibrosis, and peritubular capillary damage occur predominantly in the paler regions (Fig. S8). Automated whole-organ analysis revealed a numerical reduction in damaged tissue across all disease stages following AS0369 treatment, but statistical significance was only achieved in the two earliest stages (Fig. 3A, B). Glomerular cyst formation, a hallmark of advanced CN[7] was assessed by quantifying the areas of glomeruli and Bowman's space in whole-organ scans. This analysis revealed that this advanced CN feature could be significantly ameliorated even when therapy started at stages 3 and 4 (Fig. 3A,C). This finding was further supported by automated analysis of Bowman's space on whole-organ sections, which showed increased Bowman's space area at stage 3 and 4 post-BDL. Notably, AS0369 therapy significantly mitigated this pathological expansion (Fig. 3C).

A typical response to tissue injury is replacement proliferation to regenerate the lost tissue. Following BDL, tubular epithelial cells begin to proliferate because of cell death (Fig. 3D). AS0369 treatment significantly reduced proliferation when initiated on stage 1 or 3, while no signification reduction was observed for stage 2 or 4 (Fig. 3E). Compromised capillary integrity, a further feature of BDL-induced damage (Fig. 4A), was also improved by AS0369 when therapy began on stage 1 or 2 post-BDL (Fig. 4B); however, statistical significance was not achieved for later stages. Renal fibrosis, a key feature of CN, was strongly reduced by AS0369 treatment at all disease stages except for stage 3 where statistical significance was not reached (Fig. 4C, D). Additionally, *Egr1* RNA, a well-established marker of tissue inflammation and fibrogenesis,[7] was significantly reduced by AS0369 therapy at stages 1 and 2 but not at the later stages (Fig. 4E).

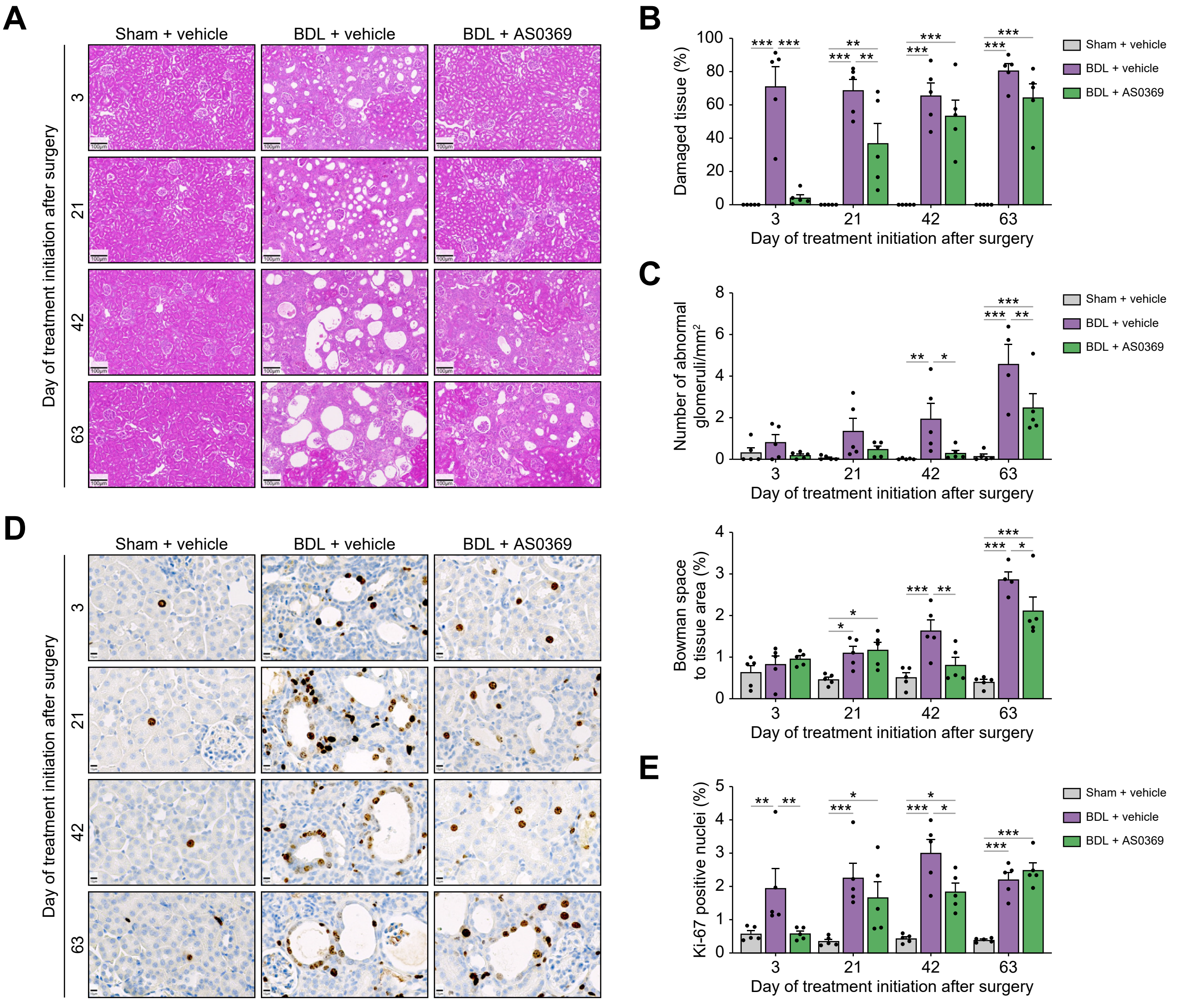

Fig. 3. Renal tissue damage and replacement proliferation after obstructive cholestasis and therapy by AS0369. (A) H&E-stained renal tissue sections; scale bars: 100 μm. (B) Quantification of damaged tissue; (C) Number of abnormal glomeruli per area and overall area of Bowman space to the total tissue area; (D) Proliferating cells (brown nuclei) based on ki67 staining; scale bars: 10 μm; (E) Percentage of Ki-67 positive nuclei. *p <0.05; **p <0.01; ***p <0.001; Tukey's multiple comparisons test. Data are presented as mean± SEM. n = 4-5 mice per group; the dots in the bars represent individual mice. BDL: bile duct ligation.

Smaller therapeutic effect size on the liver compared to kidney after systemic ASBT inhibition in obstructive cholestasis

Next, we analyzed the livers of the same mice whose kidneys have already been described above, to directly compare the therapeutic efficacy of systemic ASBT inhibition in both organs. No significant reduction in the activity of transaminases was achieved with AS0369 (Fig. 5A,B). BDL caused a dilation of bile canaliculi that appeared to be ameliorated by AS0369 in tissue sections immunostained against CD13 (Fig. 5C). To quantify this effect, the bile canalicular diameter was determined in whole slide scans to avoid any selection bias. When all bile canalicular diameters were included into the analysis, an increase in the mean canalicular diameter due to BDL was seen, but the effect size of AS0369 therapy was extremely small (Fig. 5D). However, when only bile canaliculi with an abnormally wide diameter were included in the analysis (defined as bile canaliculi with diameter exceeding Q3+1.5 × [Q3–Q1], where Q3 and Q1 represent the upper and lower quartiles of the sham control distribution), a larger AS0369 treatment-associated reduction in canaliculi diameter was readily observed (Fig. 5D, right panel). It should be noted that when interpreting the results of the bile canaliculi analysis, diameters were measured for every pixel of the medial lines, to account for diameter variability along individual branches, leading to very high numbers of analyzed structures. To avoid test overfitting due to large numbers, we performed stratified statistical sampling using a fixed number of measurements per stratum (Fig. S9). Here, strata refer to the experimental groups formed by each unique combination of treatment and

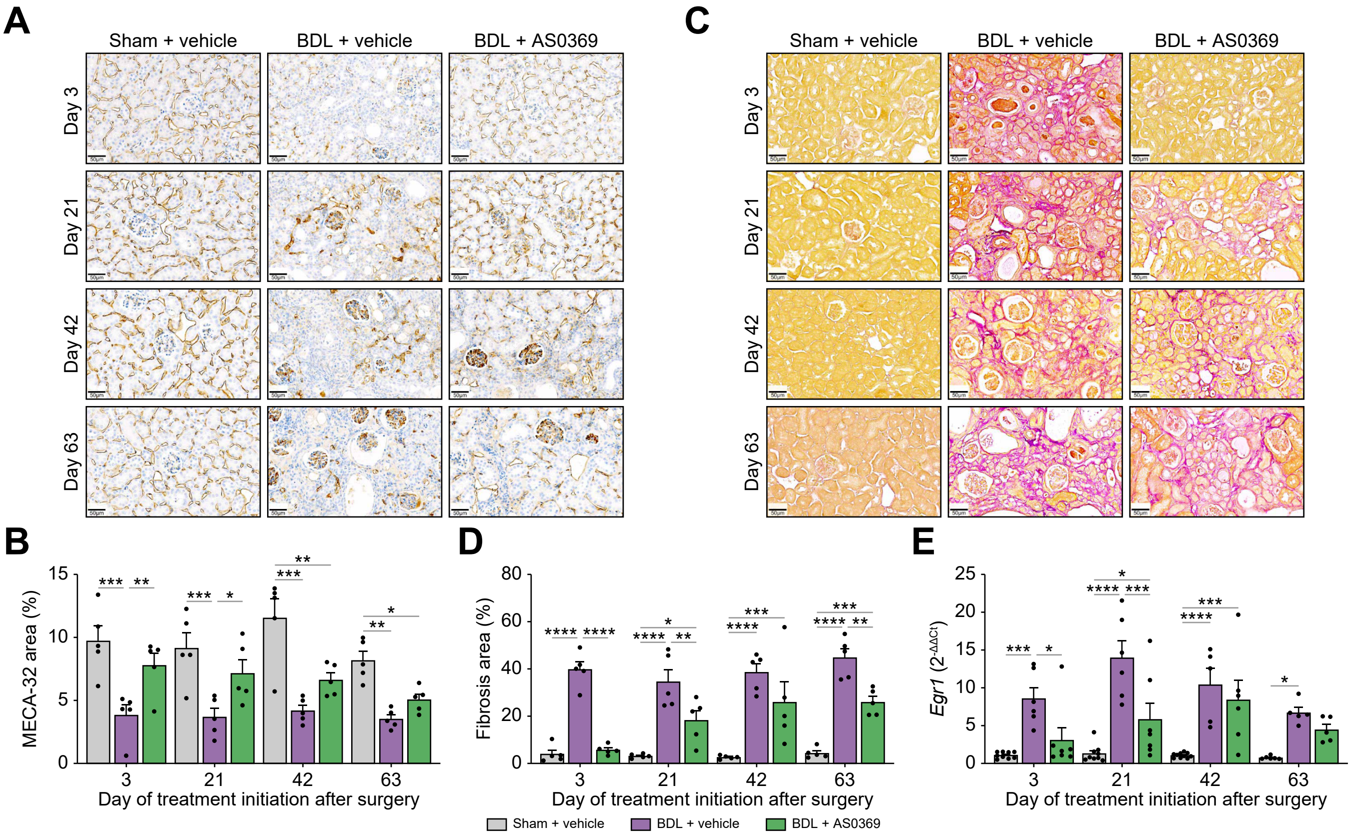

Fig. 4. Damage of peritubular capillaries and renal fibrosis after obstructive cholestasis and rescue by AS0369 treatment. (A, B) Immunostaining of the endothelial cell marker MECA-32 and corresponding quantifications; scale bars: 50 μm. (C, D) Sirius red staining and corresponding quantifications; scale bars: 50 μm. (E) RNA expression of *Egr1* in kidney tissues. **p* <0.05; ***p* <0.01; ****p* <0.001; *****p* <0.0001; Tukey's multiple comparisons test. Data are presented as mean± SEM. n = 5-7 mice per group; the dots in the bars represent individual mice. BDL: bile duct ligation.

timestamp. Initially, we selected a large subset comprising 1,000,000 measurements per group, subsequently reducing the subset sizes by one-tenth increments, down to 1,000 measurements, and examined their statistical features (mean and standard deviation) compared to the base line of the original groups. The analysis showed that the statistical features remained consistent across subsets down to sample sizes of 10,000 measurements, with maximum changes of 0.49% in the mean and 10.67% in the standard deviation compared to the original sample. In contrast, for subsets of 1,000 measurements maximal changes were 2.19% in the mean and 10.60% in the standard deviation (Fig. S9).

Quantification of the cholangiocyte marker CK19-positive area on whole slide scans revealed a numerical reduction with AS0369 therapy in all disease stages, but statistical significance was only obtained for day 21 (Fig. 5E,F). Analysis of the Sirius red-positive fibrotic area showed a reduction with AS0369 therapy, particularly in the perisinusoidal region, with a relatively small effect size for all disease stages, but statistical significance was only reached for day 42 (Fig. S10).

Amelioration of BDL-induced gene expression changes by systemic ASBT inhibition

RNA-sequencing (RNA-seq) analysis of the above-described kidney tissue specimens (Fig. 1A) was performed using genome-wide expression changes as an unbiased measure of the efficacy of therapy. Sham-operated controls and vehicle-treated BDL mice clustered in distinct regions of the principal component analysis (Fig. 6A). Treatment of BDL mice with AS0369 led to a shift almost completely back to controls when the therapy began at stage 1 (3 days after BDL). The extent of this shift decreased when therapy began later. Number, fold-change, and *p* values of the individual genes were visualized by volcano plots, illustrating the stage dependence of the ASBT-inhibiting therapy (Fig. 6B). For example, the number of downregulated genes in BDL mice in the day 3-cohort (stage 1) was 3,522 (BDL and vehicle), compared to only 372 down-regulated genes in BDL mice who were treated with AS0369. The corresponding numbers were 3,538 (BDL and vehicle) and 2,672 (BDL and AS0369) when AS0369 therapy was initiated at stage 4 (63 days after BDL).

The efficacy of systemic ASBT inhibition was visualized by contrasting the ratio of BDL (vehicle) *vs.* sham (vehicle) against the ratio of BDL (AS0369) *vs.* sham (vehicle), with each dot representing an individual gene in the resulting "differentiation pattern (DiPa) plot" (Fig. 6C). If the treatment with AS0369 did not affect gene expression, the individual genes would cluster on and around the diagonal. In contrast, an effect on gene expression is represented by expression pattern groups 1a and 1b, including genes that are up- (or down-) regulated by BDL but brought back to their normal

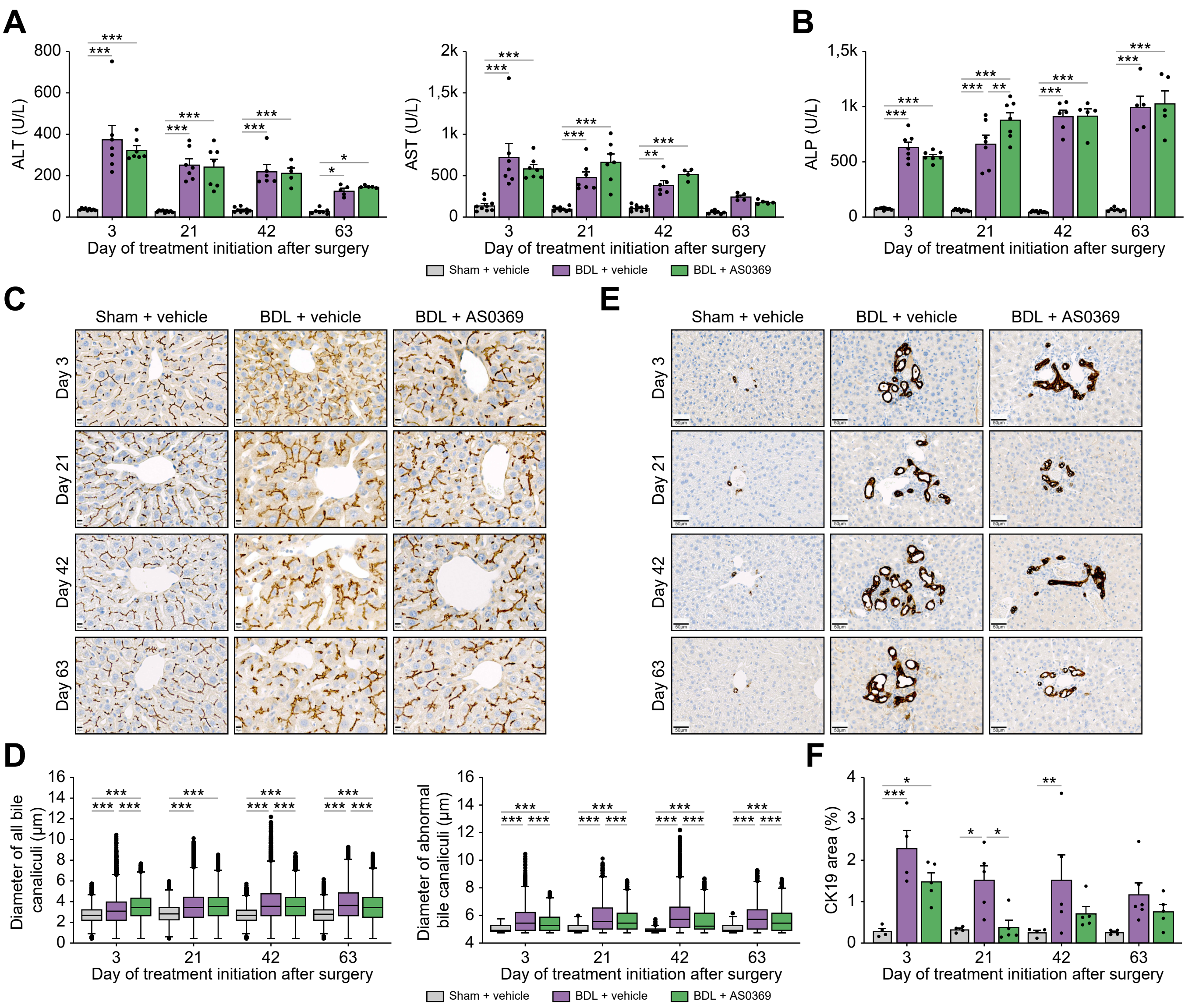

Fig. 5. Characterization of liver damage after obstructive cholestasis and influence of AS0369 treatment. (A, B) Transaminases and alkaline phosphatase activities measured in plasma; n = 4-10 mice per group; the dots in the bars represent individual mice. (C) Visualization of bile canaliculi by immunostaining against CD13; scale bars: 10 µm; (D) Quantification of bile canalicular diameter; n = 5 mice per group. (E) Visualization of bile ducts by immunostaining against CK19; scale bars: 50 µm; (F) Percentage of CK19-positive area to the total tissue area; n = 4-5 mice per group; the dots in the bars represent individual mice. Data are presented as mean± SEM. *p <0.05; **p <0.01; ***p <0.001; Tukey's multiple comparisons test. BDL: bile duct ligation; ALT: alanine transaminase; AST: aspartate transaminase; ALP: alkaline phosphatase.

expression range by AS0369 therapy. Correspondingly, expression pattern groups 2a and 2b represent a partial success regarding AS0369 counteracting gene expression changes induced by BDL. On the contrary, for genes in the purple region (nta= not treatable) AS0369 therapy would even enhance the effect of BDL. It is interesting to note the relatively high number of genes in expression pattern groups 2a and 2b when therapy was initiated at stage 1 after BDL, which decreased when treatment was initiated in more advanced disease stages. Correspondingly, the number of genes on and around the diagonal increased the later ASBT inhibition was initiated. Expression pattern groups 3a and 3b represent genes that were not influenced by BDL but were up- or downregulated by AS0369.

Since the efficacy of AS0369 therapy decreased when initiated at stage 4 compared to stage 1, we next focused specifically on the set of initially (stage 1) responsive genes (Fig. S11A; Table S3). Genes falling into expression pattern group 1a upon treatment initiation at stage 1 (n = 2,342) were, when therapy was initiated stage 4, primarily found in the nta expression pattern group (n = 1657), while 470 genes "migrated" to expression pattern group 2a and 215 genes remained in 1a; a similar pattern was observed for genes initially located in expression pattern group 1b (Fig. S11A). This highlights the stage-dependent effect of the treatment: with delayed initiation, genes that were fully rescued at stage 1 (1a) were largely unaffected at stage 4 (nta), some were only partially rescued (2a), and only a small fraction remained fully

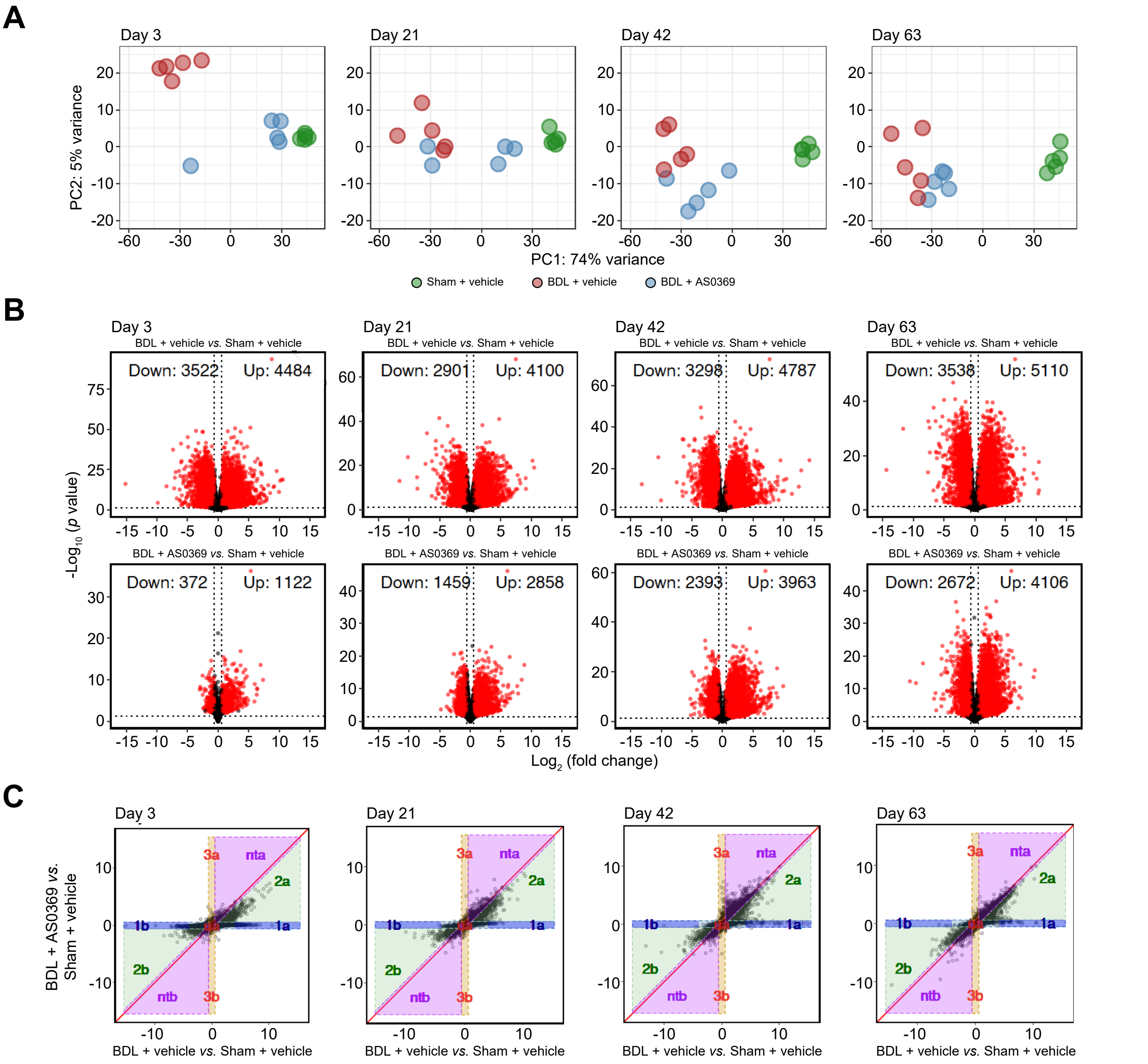

Fig. 6. RNA-sequencing analysis of renal tissue after obstructive cholestasis with and without ASBT inhibition. (A) Principal component analysis; each dot represents an individual mouse. (B) Volcano plots illustrate differentially expressed genes; red dots: significantly deregulated genes; black dots: not significantly altered genes; vertical dotted lines: threshold values $\log_2(1.5)$ and $-\log_2(1.5)$; horizontal dotted line: threshold value 0.05 transformed via $-\log_{10}(0.05)$. (C) DiPa-plots illustrating the therapeutic efficacy of AS0369. The nta area contains "not treatable" genes. n = 5 mice per group. BDL: bile duct ligation; DiPa, differentiation pattern.

rescued (1a). Conversely, most genes initially in expression pattern groups nta and ntb remained in these groups upon later treatment initiation. Interestingly, genes found in the subsets of expression pattern groups 1a and 1b which are here denoted as "extreme regions" (1a extreme or 1b extreme), for which BDL caused a particularly strong (at least 17-fold) up- or downregulation, were mostly responsive and primarily migrated to expression pattern group 2a and 2b (Fig. S11A). This demonstrates that the expression of genes most strongly altered due to BDL at stage 1, were still modified with partial success by AS0369 treatment initiated at stage 4 after BDL.

Finally, to gain functional insights, we analyzed Gene Ontology groups influenced by BDL as well as BDL followed by AS0369 therapy. Genes upregulated in response to BDL were mostly inflammation-associated, such as 'inflammatory response', 'interleukin-6', 'neutrophil chemotaxis', 'ERK1, ERK2', and 'lipopolysaccharide' (Fig. S11B). These motives were similar in all disease stages. Among downregulated genes, mostly metabolism-associated functions were enriched, such as beta-oxidation or cholesterol biosynthesis. Upregulated genes that were 'treatable' (1a and 2a) at different time points showed an enrichment of inflammation-associated

motives (Fig. S12); downregulated genes (1b and 2b) that were not treatable were enriched with metabolism-associated motives. This suggests that treatable and not treatable genes do not show major differences with respect to their Gene Ontology groups.

A similar RNA-seq analysis as for the kidney was also performed in liver tissue of the same **mice** (Figs 7, S13, and S14; Table S4). An important difference compared to kidney was the much smaller effect size of AS0369 therapy for the liver. Principal component analysis of the liver highlights the strong effect of BDL on global gene expression and the only moderate effect of AS0369, which decreased from the stage 1 to stage 4 treatment groups (Fig. 7A). This corresponds to the results of the volcano plots, where 2,759 genes were downregulated by BDL in the stage 1 cohort (vehicle control) compared to only 1,334 downregulated genes with AS0369 therapy (Fig. 7B). Up to stage 4 these numbers decreased to 1,567 (BDL, vehicle) and 1,182 (BDL, AS0369 therapy), respectively (Fig. 7B). The decrease in deregulated genes when treatment is initiated after longer periods after BDL is

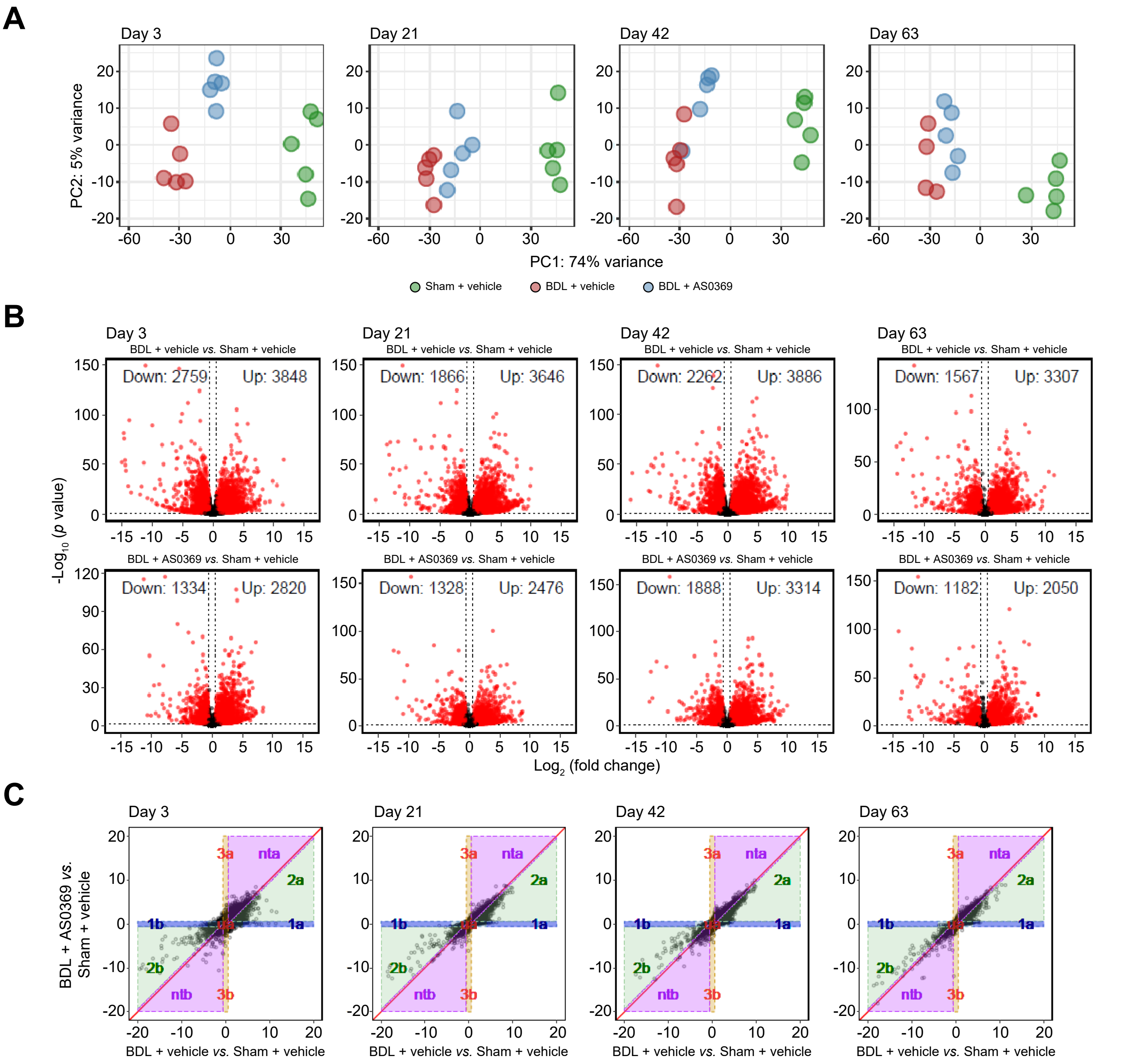

Fig. 7. RNA-sequencing analysis of liver tissue after obstructive cholestasis with and without ASBT inhibition. (A) Principal component analysis; each dot represents an individual mouse. (B) Volcano plots illustrate differentially expressed genes; red dots: significantly deregulated genes; black dots: not significantly altered genes; vertical dotted lines: threshold values $\log_2(1.5)$ and $-\log_2(1.5)$; horizontal dotted line: threshold value 0.05 transformed via $-\log_{10}(0.05)$. (C) DiPa-plots illustrating the therapeutic efficacy of AS0369. n = 5 mice per group. BDL: bile duct ligation; DiPa, differentiation pattern.

probably explained by hepatic adaptation to the cholestatic situation.[8] The smaller effect size of AS0369 therapy in the liver compared to the kidney is also illustrated by the relatively small fraction of genes in expression pattern groups 1a and 1b of the DiPa-plots (Figs 7C and S13). Gene Ontology analysis showed an enrichment of inflammation-associated motifs among upregulated genes and metabolism-associated motifs among downregulated genes (Fig. S13 and 14).

Systemic ASBT inhibition in healthy mice causes no morphological or functional alterations and only minor molecular changes in the liver and kidneys

To assess the influence of ASBTi in non-cholestatic controls, mice on day 3 after sham operation were repeatedly treated with AS0369 or vehicle twice daily for 4 weeks (Fig. 8A). Analysis of body weight changes revealed no difference in the ASBTi-treated group compared to the vehicle controls (Fig. 8B). No significant differences in BA concentrations in blood and urine were observed in response to ASBTi treatment; in contrast, ASBTi caused significant reduction of BA concentrations in bile (Fig. 8C). Analysis of total bilirubin, liver enzymes, albumin, cystatin C, blood urea nitrogen, and kidney injury biomarkers (KIM-1, NGAL) in blood revealed no significant changes between ASBTi- and vehicle-treated mice (Fig. 8C-E). In agreement, histological analysis of the liver and kidneys revealed no alterations (Fig. S15). Thus, ASBTi treatment in healthy mice causes no morphological/functional adverse effects.

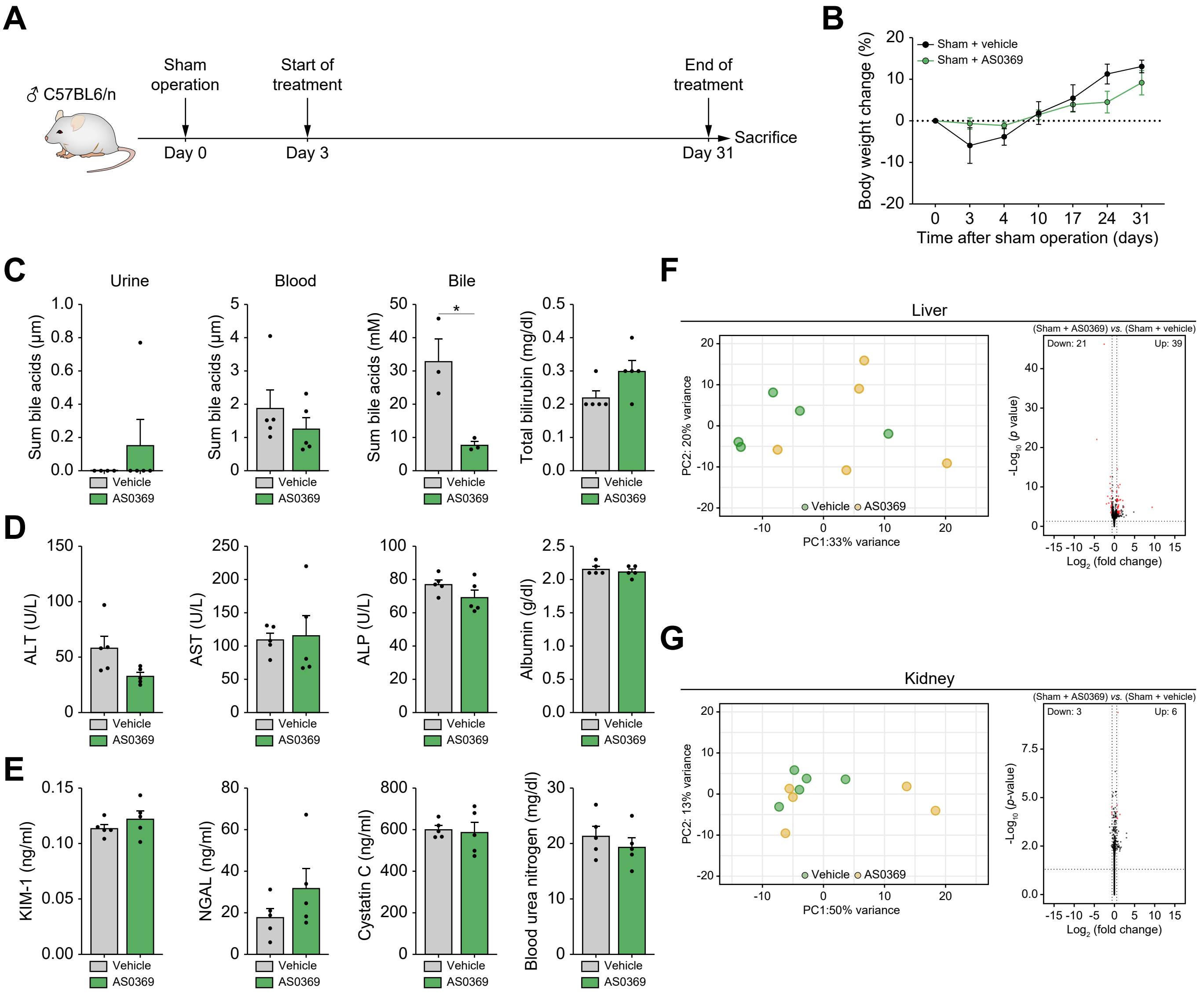

Fig. 8. Systemic ASBT inhibition has only minor effects in the liver and kidneys of healthy mice. (A) Experimental design. (B) Body weight changes. (C) Sum bile acid concentrations in blood, urine and bile (*p = 0.0206; Unpaired t test), and total bilirubin levels in blood. (D) Blood biomarkers of liver injury or function. (E) Biomarkers of kidney injury in urine (KIM-1, NGAL) and kidney functions in blood (cystatin C, blood urea nitrogen). Data are presented as mean± SEM. n = 3-5 mice per group; the dots in the bars represent individual mice. (F, G) RNA-sequencing analysis of liver (F) and kidney (G) tissues including principal component analysis (left panels) and volcano plots illustrating differentially expressed genes (right panels); red dots: significantly deregulated genes; black dots: not significantly altered genes; vertical dotted lines: threshold values $\log_2(1.5)$ and $-\log2(1.5)$; horizontal dotted line: threshold value 0.05 transformed via $-\log_{10}(0.05)$; n = 5 mice per group. ALT: alanine transaminase; AST: aspartate transaminase; ALP: alkaline phosphatase; KIM-1: kidney injury molecule1; NGAL: neutrophil gelatinase-associated lipocalin.

To investigate possible molecular changes, RNA-seq analysis was performed in the liver and kidney tissues. In the liver, a few moderate gene expression changes were observed (Fig. 8F; Table S5A). As expected, Cyp7a1 was among the upregulated genes, since ASBTi decreases BA reabsorption from the intestine and the upregulation of genes responsible for BA synthesis in hepatocytes represents an adaptive response. Nevertheless, the extent of global expression changes in the liver in response to AS0369 is small. In the kidney, only very minor gene expression changes were observed after ASBTi (Fig. 8G; Table S5B).

Discussion

CN is a severe, often lethal complication of liver disease associated with cholestasis and represents an unmet therapeutic need. To study the therapeutic efficacy of the systemically bioavailable ASBT inhibitor AS0369, we used an animal model of obstructive cholestasis where four distinct stages ranging from an early to a severe stage of CN can be reproducibly generated. Mice in stages 1 to 4 were treated for 4 weeks with two oral doses of AS0369 per day. For interpretation of the data, it should be considered that the time periods of the four disease stages (days 1, 21, 42 and 63 after BDL) indicate the beginning of the 4-week treatment period with AS0369 or vehicle. Data after 4 weeks of AS0369 therapy were then compared to the corresponding vehicle controls.

The results of this study demonstrate that systemic ASBT inhibition caused specific improvements in all stages, but the effect on some disease features was stage dependent. In all stages, AS0369 therapy caused an increase of total BA concentrations in urine and a reciprocal BA reduction in the blood, as well as in renal and liver tissues. Also, total bilirubin concentrations decreased in all disease stages. In contrast, the levels of the pTEC injury marker KIM-1 decreased only in stage 1, where AS0369 reduced KIM-1 almost to control levels. This stage dependency is probably explained by the fact that the death events for the pTECs are mostly observed early after BDL. A strong reduction of kidney tissue damage was seen in stage 1, while the effect size of AS0369 therapy decreased for stage 2 and was no longer significant for stages 3 and 4. A similar tendency was observed for the replacement proliferation of TECs and amelioration of tubular dilatation. In addition, the therapeutic effect on fibrogenesis was much stronger in early compared to late stages. Nevertheless, AS0369 therapy also ameliorated some specific features of stages 3 and 4. The size of glomeruli (glomerular cysts) begins to increase at stage 3 and progresses to extremely large glomeruli with dilated Bowman's spaces in stage 4. In both stage 3 and 4, AS0369 treatment significantly reduced glomerular cysts. The results demonstrate that the onset of therapy with systemic ASBT inhibitors should be early, ideally when cell death events are beginning for pTECs. This aspect is critical for evaluating potential indications and clinical development, as the management of AKI in patients with liver disease largely relies on serum creatinine as an indicator of kidney dysfunction, which rises after damage has occurred.[9] Besides monitoring the pTEC injury marker KIM-1 which may be an early marker of CN in patients with cholestasis,[7] monitoring serum and/or urinary NGAL is a promising strategy for detecting tubular damage (*i.e.* subclinical AKI) that preceded the functional impairment that defines AKI in the clinic. This concept (damage *vs.* function) has been emphasized in the recent ADQI (Acute Disease Quality Initiative) and ICA (International Club of Ascites) joint multidisciplinary consensus meeting.[4] Moreover, urinary levels of the latter biomarker have been shown to differentiate between prerenal AKI, hepatorenal syndrome, and acute tubular necrosis, *i.e.* AKI subtypes in patients with cirrhosis that are characterized by increasing severity of tubular injury and decreasing probability of functional recovery.[10–12] Thus, even in patients with clinically evident AKI/kidney dysfunction, low levels of damage markers may identify those with prerenal AKI, an exclusively functional and reversible form of kidney dysfunction due to volume depletion. Notably, in patients with acute tubular necrosis, urinary NGAL remained persistently high,[13] which resembles observations in animals with CN, in which ASBTi lowered NGAL levels, particularly if initiated within the first two stages of CN. In line, ASBTi decreased KIM-1, which is also increased in human AKI in the context of liver disease.[14] However, systemic ASBTi may still be therapeutically beneficial in advanced stages, since systemic and tissue levels of BAs can also be reduced in stages 3 and 4, which possibly explains the amelioration of stage-specific disease features such as glomerular cysts. Since the current study summarizes data from a 4-week treatment period with AS0369, it cannot be excluded that longer treatment periods would ameliorate advanced CN further. From a clinical perspective, ASBTi treatment, if effective in human disease, could serve as a bridging therapy until the recovery of liver function/cholestasis in those with acute or acute-on-chronic liver injury/failure, or liver transplantation.

Interestingly, a striking difference was observed in the therapeutic efficacy of systemic ASBTi in the kidney *vs.* the liver in this model of obstructive cholestasis. While the therapeutic effect of AS0369 therapy on the kidney was large, only a comparatively small effect size was observed in the liver. A consistent, statistically significant influence of AS0369 in the liver in all four disease stages was the amelioration of bile canalicular dilation caused by BDL. It is known that cholestasis leads to the dilation of bile canaliculi,[8] and the improved canalicular diameter may be a particularly sensitive marker that is responsive to the reduced total BA concentrations in the blood and liver tissue. It should be considered that hepatocytes adapt to BDL-induced cholestasis by strongly downregulating NTCP and inducing expression of sinusoidal membrane BA exporters in an attempt to reduce the hepatocyte BA burden. However, hepatocyte BA synthesis is ongoing, and plasma BAs continue to be taken up via non-NTCP BA carriers and any residual NTCP activity. In contrast to hepatocytes, renal pTECs do not show a similarly efficient adaptation since ASBT remained expressed and functional in all disease stages. Thus, protection against intracellular BA enrichment in the pTECs can be achieved solely by inhibiting ASBT. Although AS0369 increased urinary BA excretion and reduced plasma BA levels, the treatment does not block ongoing hepatocyte BA synthesis, and the new steady-state hepatic BA burden remains above the threshold for liver injury in this model of severe obstructive cholestasis. These differences in target protection achieved in the kidney but not in the liver may explain the different therapeutic efficacy of ASBTi in both organs.

Analysis of renal genome-wide expression profiles by RNA-seq after BDL demonstrated a remarkable therapeutic effect size of AS0369. In the earliest disease stage (day 3 after BDL, stage 1), AS0369 therapy rescued 89.4% of all genes downregulated in response to BDL and 74.9% of all upregulated genes. This percentage decreased when therapy began at later stages, but the number of rescued genes was still 24.5% (down) and 19.6% (up) in the most advanced stage (stage 4). Plotting the effects of BDL with vehicle *vs*. BDL with ASBT inhibition resulted in three expression pattern groups, where genes up- or downregulated in response to BDL are either completely, partially or not rescued by the therapy. A fraction of the genes completely rescued in stage 1 moved to the partially or not rescued category when the therapy began at later disease stages. RNA-seq analysis of the livers of the same mice demonstrates a smaller therapeutic effect compared to the kidney. However, in disease stage 1, AS0369 therapy rescued 51.6% of downregulated genes and 26.7% of upregulated genes in liver tissue, illustrating that the effect of the ASBTi could still be quantified.

Although systemic ASBTi efficiently enhances urinary BA excretion and reduces BA blood concentrations, it should be considered that the blood BA concentrations of BDL mice were not reduced to normal levels of less than 2 μM (in mice) after AS0369 treatment but remained between 100 and 200 μM. As noted above, this is likely explained by continued synthesis of BA in the cholestatic liver as evidenced by high levels of C4 under AS0369 exposure.[7] Future studies should address if blood BA concentrations in cholestasis can be decreased even further by combining systemic ASBTi[7,15] with additional drugs, such as synthetic FXR agonists, FGF15/19 mimetics or PPAR agonists to inhibit BA synthesis,[16–20] NTCP inhibitors to block hepatic BA uptake, or with therapeutic BAs to reduce lipophilicity of the BA pool.[21–23]

In conclusion, systemic inhibition of ASBT is an efficient therapeutic option for CN in mice. Our study demonstrated that therapy should ideally begin as soon as pTEC damage occurs to maximize treatment benefit, but ASBTi still showed an effect at late stages of CN. These observations are instrumental for identifying potential indications in humans as well as informing clinical drug development.

Affiliations

[1]*Department of Toxicology, Leibniz Research Centre for Working Environment and Human Factors, Technical University Dortmund, Ardeystr. 67, 44139, Dortmund, Germany;* [2]*Forensic Medicine and Toxicology Department, Faculty of Veterinary Medicine, South Valley University, Qena, Egypt;* [3]*Interdisciplinary Centre for Bioinformatics (IZBI) & Saxonian Incubator for Clinical Research (SIKT), University of Leipzig, Haertelstraße 16-18, 04107 Leipzig, Germany;* [4]*Dr. Margarete Fischer-Bosch Institute of Clinical Pharmacology and University of Tübingen, Auerbachstr. 112, 70376 Stuttgart, Germany;* [5]*Department of Statistics, TU Dortmund University, 44227 Dortmund, Germany;* [6]*Institute of Medical Statistics and Bioinformatics, University of Cologne, Germany;* [7]*Department of Pharmacology, Faculty of Veterinary Medicine, Sohag University, 82524 Sohag, Egypt;* [8]*Ipsen, Göteborg, Sweden;* [9]*Department of Gastroenterology, Hepatology and Infectious Diseases, University Hospital Duesseldorf, Medical Faculty at Heinrich-Heine-University, 40225 Dusseldorf, Germany;* [10]*University Clinic for Visceral Surgery and Medicine, Inselspital University Hospital, University of Bern, 3010 Bern, Switzerland;* [11]*Departments of Clinical Pharmacology, and of Biochemistry and Pharmacy, University Tuebingen, Tuebingen, Germany;* [12]*Histology Department, Faculty of Medicine, South Valley University, 83523 Qena, Egypt;* [13]*Clinic for Gastroenterology, Hepatology and Transplantation Medicine, University Hospital Essen, Essen, Germany;* [14]*Stravitz-Sanyal Institute for Liver Disease and Metabolic Health, Virginia Commonwealth University, Richmond, VA, United States;* [15]*Division of Gastroenterology and Hepatology, Department of Internal Medicine III, Medical University of Vienna, 1090 Vienna, Austria;* [16]*Department of Pediatrics, Division of Gastroenterology, Hepatology, and Nutrition, Emory University, Atlanta, GA 30322, United States*

Abbreviations

ASBT, apical sodium-dependent bile acid transporter; BAs, bile acids; BDL, bile duct ligation; CN, cholemic nephropathy; DiPa, differentiation pattern; Egr1, early growth response protein 1; KIM-1, kidney injury molecule 1; MRP, multidrug resistance-associated protein; NGAL, neutrophil gelatinase-associated lipocalin; NTCP, Na+-taurocholate co-transporting polypeptide; OAT3, organic anion transporter3; OST-α/β, Organic Solute Transporter-α/β; pTECs, proximal tubular epithelial cells.

Financial support

A.G. was funded by the German Research Foundation (DFG; Project IDs 517010379& 457840828). UH and MS were funded by the Robert Bosch Stiftung, Stuttgart, Germany. LA was funded by the DFG (the Research Training Group "Biostatistical Methods for High-Dimensional Data in Toxicology"; RTG 2624, Project P7; Project Number 427806116). MT was funded by the Austrian Science Fund FWF (F7301). P.A.D. was supported by NIH R01 DK140485. SH was supported by the BMBF (031L0257J, 031L0256C, 031L0314I, 031L0313C); DFG (HO4772/5-2); and EU (ARTEMIS/101136299).

Conflict of interest

AG and JGH have advised for Albireo. M.M. served as a speaker and/or consultant and/or advisory board member for AbbVie, AstraZeneca, Collective Acumen, Eli Lilly, Gilead, Echosens, Ipsen, Takeda and W. L. Gore & Associates and received grants from Echosens as well as travel support from AbbVie and Gilead. MT has received research grants from Albireo, Alnylam, Cymabay, Falk, Genentech, Gilead, Intercept, MSD, Takeda and Ultragenyx and travel grants from AbbVie, Falk, Gilead Intercept and Jannsen; he further has advised for AbbVie, Albireo, Agomab, BiomX, Boehringer Ingelheim, Chemomab Falk, Genfit, Gilead, Hightide, Intercept, Ipsen, Janssen, MSD, Mirum, Novartis, ProQR Therapeutics Phenex, Pliant, Regulus, Siemens and Shire and has served as speaker for Albireo, BMS, Boehringer Ingelheim, Falk, Gilead, Intercept, Ipsen, Madrigal and MSD. He is a co-inventor of patents for the medical use of norUDCA (nor-ursodeoxycholic acid/norucholic acid) filed by the Medical Universities of Graz and Vienna (service inventions). Outside of the submitted work, MS received funding from Green Cross WellBeing, Gilead Sciences, Robert Bosch GmbH, CORAT Therapeutics, HepaRegeniX GmbH, Boehringer Ingelheim, and Agena Bioscience. GS declares consulting or advisory board activities for Albireo, Ipsen, Sanofi, CSL Behring, Ideogen and Advanz Pharma and travel grants/educational support from Alnylam, Falk and Gilead. PAD has received research grant support from Albireo.

Please refer to the accompanying ICMJE disclosure forms for further details.

Authors' contributions

AG and JGH: study concept and design, data acquisition, analysis and interpretation of data, manuscript writing, funding, study supervision; MM (Myllys), DG, ZH, RH, Hannah S, RH (Hendawi) BBT, JR (Reinders), KD, MV, AS, NA, MV, TA, BH: contributed to study concept and design, data acquisition, manuscript writing, analysis and interpretation of data; AF, QS, DZ, SH: image analysis, contributed to manuscript writing, critical revision of the manuscript; JD, LA, KM, JR: bioinformatics analysis, contributed to manuscript writing, critical revision of the manuscript; UH, MS: bile acid analysis, contributed to manuscript writing, critical revision of the manuscript; ES, PÅ, EL: synthetized AS0369, contributed to study concept and design, manuscript writing, analysis and interpretation of data; KE: RNA-sequencing, contributed to bioinformatics analysis, study concept and design, data acquisition, analysis and interpretation of data, critical revision of the manuscript; TL, GS, Hartmut S, SJK, BS, MM (Mandorfer), MT, PAD: contributed to study concept and design, analysis and interpretation of data, critical revision of the manuscript.

Data availability

All data presented in this manuscript will be made available to other researchers upon request. The raw RNA-sequencing data can be accessed via the Sequence Read Archive (SRA) with the accession number PRJNA1224581.

Supplementary data

Supplementary data to this article can be found online at https://doi.org/10.1016/j.jhepr.2025.101599.

References

Author names in bold designate shared co-first authorship

[1] **Fickert P**, **Krones E**, Pollheimer MJ, et al. Bile acids trigger cholemic nephropathy in common bile-duct-ligated mice. Hepatology 2013;58:2056–2069.
[2] Fickert P, Rosenkranz AR. Cholemic nephropathy reloaded. Semin Liver Dis 2020;40:91–100.
[3] **Tonon M**, **Rosi S**, Gambino CG, et al. Natural history of acute kidney disease in patients with cirrhosis. J Hepatol 2021;74:578–583.
[4] Nadim MK, Kellum JA, Forni L, et al. Acute kidney injury in patients with cirrhosis: acute Disease Quality Initiative (ADQI) and International Club of Ascites (ICA) joint multidisciplinary consensus meeting. J Hepatol 2024;81:163–183.
[5] European Association for the Study of the Liver. EASL Clinical Practice Guidelines for the management of patients with decompensated cirrhosis. J Hepatol 2018;69:406–460.
[6] Fickert P. Detour of bile acid routes as therapeutic roadmap for cholemic nephropathy. J Hepatol 2024;80:188–190.
[7] Ghallab A, Gonzalez D, Strangberg E, et al. Inhibition of the renal apical sodium dependent bile acid transporter prevents cholemic nephropathy in mice with obstructive cholestasis. J Hepatol 2024;80:268–281.
[8] Ghallab A, Hofmann U, Sezgin S, et al. Bile microinfarcts in cholestasis are initiated by rupture of the apical hepatocyte membrane and cause shunting of bile to sinusoidal blood. Hepatology 2019;69:666–683.
[9] Ghallab A, Mandorfer M, Stirnimann G, et al. Enteronephrohepatic circulation of bile acids and therapeutic potential of systemic bile acid transporter inhibitors. J Hepatol 2025;83:1204–1217.
[10] Huelin P, Solà E, Elia C, et al. Neutrophil gelatinase-associated lipocalin for assessment of acute kidney injury in cirrhosis: a prospective study. Hepatology 2019;70:319–333.
[11] Allegretti AS, Parada XV, Endres P, et al. Urinary NGAL as a diagnostic and prognostic marker for acute kidney injury in cirrhosis: a prospective study. Clin Transl Gastroenterol 2021;12:e00359.
[12] Gambino C, Piano S, Stenico M, et al. Diagnostic and prognostic performance of urinary neutrophil gelatinase-associated lipocalin in patients with cirrhosis and acute kidney injury. Hepatology 2023;77:1630–1638.
[13] Solé C, Ma AT, Solà E, et al. Sequential changes in urinary biomarker levels in patients with cirrhosis and severe hepatorenal syndrome. Liver Int 2021;41:2729–2732.
[14] Lei L, Li LP, Zeng Z, et al. Value of urinary KIM-1 and NGAL combined with serum Cys C for predicting acute kidney injury secondary to decompensated cirrhosis. Sci Rep 2018;8:7962.
[15] Caballero-Camino FJ, Rodrigues PM, Wångsell F, et al. A3907, a systemic ASBT inhibitor, improves cholestasis in mice by multiorgan activity and shows translational relevance to humans. Hepatology 2023;78:709–726.
[16] Chiang JYL, Ferrell JM. Discovery of farnesoid X receptor and its role in bile acid metabolism. Mol Cell Endocrinol 2022;548:111618.
[17] Kunst RF, Bolt I, van Dasselaar RDJ, et al. Combined inhibition of bile salt synthesis and intestinal uptake reduces cholestatic liver damage and colonic bile salts in mice. JHEP Rep 2024;6:100917.
[18] Kouno T, Liu X, Zhao H, et al. Selective PPARδ agonist seladelpar suppresses bile acid synthesis by reducing hepatocyte CYP7A1 via the fibroblast growth factor 21 signaling pathway. J Biol Chem 2022;298:102056.
[19] Schattenberg JM, Pares A, Kowdley KV, et al. A randomized placebo-controlled trial of elafibranor in patients with primary biliary cholangitis and incomplete response to UDCA. J Hepatol 2021;74:1344–1354.
[20] Honda A, Ikegami T, Nakamuta M, et al. Anticholestatic effects of bezafibrate in patients with primary biliary cirrhosis treated with ursodeoxycholic acid. Hepatology 2013;57:1931–1941.
[21] Fuchs CD, Dixon ED, Hendrikx T, et al. Tetrahydroxylated bile acids improve cholestatic liver and bile duct injury in the Mdr2(-/-) mouse model of sclerosing cholangitis via immunomodulatory effects. Hepatol Commun 2022;6:2368–2378.
[22] Krones E, Eller K, Pollheimer MJ, et al. NorUrsodeoxycholic acid ameliorates cholemic nephropathy in bile duct ligated mice. J Hepatol 2017;67:110–119.
[23] Trauner M, Fuchs CD, Halilbasic E, et al. New therapeutic concepts in bile acid transport and signaling for management of cholestasis. Hepatology 2017;65:1393–1404.

Keywords: cholestasis; acute kidney injury; bile duct ligation; entero-nephro-hepatic circulation; bile cast.
Received 19 December 2024; received in revised form 2 September 2025; accepted 16 September 2025; Available online 23 September 2025

Journal of Hepatology, Volume *7*

Supplemental information

Stage-dependent effects of systemic ASBT inhibition in a cholestasis-induced cholemic nephropathy mouse model

Ahmed Ghallab, Maiju Myllys, Daniela González, Adrian Friebel, Zaynab Hobloss, Reham Hassan, Hannah Schmidt, Qasim Siddiqui, Deng Zhipeng, Rama Hendawi, Brigitte Begher-Tibbe, Joerg Reinders, Katharina Derksen, Ute Hofmann, Julia C. Duda, Lucia Ameis, Kathrin Möllenhoff, Abdellatief Seddek, Noha Abdelmageed, Ellen Strängberg, Peter Åkerblad, Mihael Vucur, Tom Luedde, Guido Stirnimann, Matthias Schwab, Tahany Abbas, Benedikt Hild, Hartmut Schmidt, Saul J. Karpen, Benedikt Simbrunner, Mattias Mandorfer, Jörg Rahnenführer, Karolina Edlund, Stefan Hoehme, Michael Trauner, Paul A. Dawson, Erik Lindström, and Jan G. Hengstler

Stage-dependent Effects of Systemic ASBT Inhibition in a Cholestasis-induced Cholemic Nephropathy Mouse Model

Ahmed Ghallab, Maiju Myllys, Daniela González, Adrian Friebel, Zaynab Hobloss, Reham Hassan, Hannah Schmidt, Qasim Siddiqui, Deng Zhipeng, Rama Hendawi, Brigitte Begher-Tibbe, Joerg Reinders, Katharina Derksen, Ute Hofmann, Julia C Duda, Lucia Ameis, Kathrin Möllenhoff, , Abdellatief Seddek, Noha Abdelmageed, Ellen Strängberg, Peter Åkerblad, Mihael Vucur, Tom Luedde, Guido Stirnimann, Matthias Schwab, Tahany Abbas, Benedikt Hild, Hartmut Schmidt, Saul J Karpen, Benedikt Simbrunner, Mattias Mandorfer, Jörg Rahnenführer, Karolina Edlund, Stefan Hoehme, Michael Trauner, Paul A Dawson, Erik Lindström, Jan G Hengstler

Table of contents

Supplementary Methods 3

Supplementary Figures 8

Supplementary Tables 23

Supplementary References 26

Supplementary Methods

Induction of obstructive cholestasis in mice and administration of the systemic ASBT inhibitor. Eight-to-ten-week-old male C57BL/6N mice (Janvier Labs, France) were used. Obstructive cholestasis was induced by ligating the extrahepatic bile duct (BDL) at a position between the gallbladder and the duodenum. Sham control mice underwent the same operative procedure but without BDL. The BDL mice received AS0369 (60 mg/kg) or vehicle (0.5% methyl cellulose and 0.1 % tween 80) orally by gavage twice per day for 4 weeks starting on either day 3, 21, 42, or 63 after the surgery. The sham operated mice received AS0369 for 4 weeks starting on day 3 after the surgery or vehicle starting on either day 3, 21, 42, or 63 after the surgery.

Sample collection and processing. Blood sampling. Heart blood samples were collected from anaesthetised mice in syringes precoated with heparin and used freshly for analyses of liver damage biomarkers. The remaining blood samples were centrifuged, and the isolated plasma were stored at -80 °C until used for analysis. ***Urine sample collection.*** 24-hour urine was collected in single mouse metabolic cages (Tecniplast Deutschland GmbH, Germany) and stored at -80 °C until used for analysis. ***Tissue sample collection.*** Liver and kidney tissue samples were collected from defined anatomical positions and processed using standard protocols as previously described [1]. ***Biochemical analysis.*** Analysis of total bilirubin, alanine transaminase, aspartate transaminase, albumin, blood urea nitrogen and alkaline phosphatase was done in freshly collected heparinized heart blood using the Piccolo Xpress Chemistry Analyzer and the Piccolo General Chemistry 13 Panel Kit.

Bile acid assay. Concentrations of bile acids in liver and kidney tissues were determined by negative electrospray (ESI) liquid chromatography tandem mass spectrometry (LC-MS/MS) in multiple-reaction-monitoring (MRM) mode on an Agilent 6495B triple quadrupole mass spectrometer (Agilent, Germany) coupled to an Agilent Infinity II HPLC system as described previously [2, 3]. Bile acid analysis in bile, blood plasma and urine was accomplished by LC-MS as published previously [4]. Briefly, the urine and bile samples were diluted 10-fold and 100-fold with water for analysis of bile acids while

plasma samples were used undiluted. Proteins were precipitated with 80% methanol containing internal standards. After centrifugation, 5 µL of the supernatant were injected for LC-MS-measurement on a QExactive mass spectrometer coupled to a Vanquish Horizon UHPLC (ThermoFisher). Quantification was done using the Skyline software (version 24.1).

MALDI-MS-Imaging and quantifications. MALDI-MS images of taurocholic acid (TCA) were acquired on a timsTOF fleX (Bruker Daltonics, Bremen, Germany) as published earlier [1]. Briefly, 5 µm-thick frozen liver and kidney tissue sections were sprayed with 2-mercaptobenzothiazole and measured in negative mode over a mass range of 85-800 m/z and internally calibrated on taurocholic acid and the 2-mercaptobenzothiazole matrix peak. Data was evaluated using the Scils Lab MVS software (version 2024b Pro, build 12.01.16059). Since TCA and TMCA have the same sum formula (same m/z), the here reported TCA-signal is indeed the sum of TCA and TMCA.

NGAL, KIM1, and cystatin C assays. Concentrations of NGAL/Lcn-2 were analyzed in mouse plasma using the Mouse Lipocalin-2/NGAL DuoSet ELISA kit plus the DuoSet ELISA Ancillary Reagent Kit 2 from R&D systems according to manufacturer's protocol. Concentrations of Kim-1 were determined in mouse plasma using the Mouse TIM1 ELISA Kit (KIM-1) from Abcam according to the manufacturer's protocol. Concentrations of cystatin C were determined in plasma using the Mouse Cystatin C ELISA Kit from Abcam according to manufacturer's protocol.

Histopathology. Histopathological analysis was performed in 4 µm-thick paraformaldehyde-fixed paraffin-embedded liver and kidney tissue sections. Hematoxylin and eosin (H&E) staining was performed using the Discovery Ultra Automated Slide Preparation System [1, 5]. Sirius red staining was done using a commercially available kit, according to the manufacturer's instruction [6]. Whole slide scans were acquired (Axio Scan.Z1) for the quantifications and representative snapshots are shown in the result section.

Immunohistochemistry. Immunostainings were performed in 4 µm-thick paraformaldehyde-fixed paraffin-embedded liver or kidney tissue sections using the Discovery Ultra Automated Slide Preparation System, as previously described [1]. The used antibodies and staining conditions are given in Table S1. Whole slide scans were acquired (Axio Scan.Z1) for the quantifications and representative snapshots are shown in the result section.

Image analysis. Image processing and quantification of series of brightfield tissue slides stained with H&E, Sirius Red, CK19, MECA-32, ASBT, NTCP, Ki-67, and CD13, was conducted to segment relevant regions of interest (ROI) and investigate region-specific features as described below:

For the H&E, ASBT, and MECA-32 staining, tissue and ROI segmentation was performed interactively with QuPath [7] using Random Trees R-Tree [8] or Artificial Neural Network [9] based Pixel classifiers, working at 1.77 µm/px (ROI) / 14.15 µm/px (Tissue) resolution on the Eosin channel (H&E staining), and at 0.22/0.88 µm/px resolution on the DAB channel (ASBT and MECA-32 staining).

For the Sirius Red, CK19, NTCP, and CD13 staining, U-Net convolutional neural networks [10] were trained and deployed for the automated semantic segmentation of tissue and ROIs, using the nnU-Net framework [11]. Training data was generated interactively using QuPath for representative image subsets and downscaled by a factor of two (0.44 µm/px) due to GPU memory limitations for U-Net training. The glomeruli segmentation model was trained with data from H&E and MECA-32 staining in order to focus on structural aspects for a staining independent model, improving generalization.

Nuclei segmentation in Ki-67 staining was performed using the StarDist [12] algorithm. For subsequent classification into Ki-67+/-nuclei, an ANN-based object detector was interactively trained in QuPath.

Quantification of H&E, Sirius Red, CK19, and MECA-32 was implemented as relative ROI area measurement normalized by tissue area. For Ki-67 quantifications, the density of Ki-67+ nuclei were computed.

For the H&E staining, glomeruli with dilated Bowman's space (BS) were defined as glomeruli with BS area exceeding $Q3+1.5\times(Q3-Q1)$, where Q3 and Q1 represent the

upper and lower quartiles of the sham controls (from all analysed time periods) distribution. The number of dilated glomeruli was normalized by tissue area.

To assess bile canaliculi (BC) diameters in the CD13 staining, a connectivity preserving medial axis skeleton was computed for each BC object using a thinning algorithm [13]. For each skeleton pixel, its distance to the boundary was measured. These measurements provided a comprehensive representation of BC diameter. To minimize the influence of outliers and false-positive segmentations, any diameter measurements above the 99th percentile were discarded. The dilated diameters were defined as BC with diameter exceeding $Q3+1.5\times(Q3-Q1)$, where Q3 and Q1 represent the upper and lower quartiles of the sham controls (from all analysed time periods) distribution.

Gene expression analysis

RNA isolation and cDNA synthesis. RNA was isolated from snap-frozen liver and kidney tissue samples using RNeasy Mini Kit, followed by cDNA synthesis using the High-Capacity cDNA Reverse Transcription Kit. ***qRT-PCR assays.*** Quantitative RT-PCR analyses were performed with cDNA using TaqMan 7500 Real-Time PCR, TaqMan universal PCR Master Mix, and TaqMan gene expression assays (Table S2). The data were normalized to the housekeeping gene GAPDH, and gene expression changes were calculated using the ΔΔCt method. The values were expressed as fold changes over the corresponding control samples.

RNA-sequencing. RNA was isolated from liver and kidney tissue using the RNeasy Mini Kit (Qiagen). RNA integrity was assessed on a 2100 Bioanalyzer with the RNA 6000 Nano Kit (Agilent Technologies); all samples had an RNA Integrity Value (RIN) above 8 or slightly below (7.8, 7.9 and 7.9). RNA concentrations were measured on a Qubit 4 Fluorometer with the RNA BR Assay Kit (Thermo Fisher, Waltham, USA). Sequencing libraries were generated from 500 ng RNA, using the TruSeq Stranded mRNA Kit with unique dual indexes (Illumina, San Diego, CA, USA), according to the manufacturer's protocol. Quantification of the final libraries was performed with the Qubit 1X dsDNA HS Assay Kit (Thermo Fisher, Waltham, USA), and library sizes were checked on an Agilent 2100 Bioanalyzer with the DNA 1000 Kit (Agilent Technologies, Santa Clara, CA, USA).

The libraries were then normalized, pooled, diluted to 1.015-1.05 pM, and paired end sequenced (bp) using the 500/550 High Output Kit v2.5 (Illumina, San Diego, CA, USA) on an Illumina NextSeq 550.

Bioinformatics. The transcript quantification and mapping of the FASTQ files were pre-processed with the software salmon, using option 'partial alignment' and the online provided decoy-aware index for the mouse genome [14]. All further analyses were performed with R, version 4.4.1 [2]. Transcript reads were summarized on gene level using the R package tximeta [15]. The following analyses were performed identically, but independently on the kidney and liver samples. For pre-filtering, from all 35,727 genes that have reads, those with less than 10 reads across all mice were removed from the data, such that 20,982 genes for kidney and 19,306 genes for liver remained. Principal component analysis was performed using the top 1,000 most variable genes after variance stabilization of the gene counts. Differential gene expression analysis was calculated using the R package DESeq2 [16]. A general linear model with one factor that combines the treatments "Sham + vehicle", "BDL + vehicle" and "BDL + AS0369" with the time points day 3, day 21, day 42, and day 63 was fitted to calculate differentially expressed genes (DEGs). This leads to a single factor model with 12 levels "Sham + vehicle, day 3", "Sham + vehicle, day 21", …, "BDL + AS0369, day 42", "BDL + AS0369, day 63". DEGs were calculated for the comparisons "Sham + vehicle vs. BDL + vehicle" at each time point. For more reliable effect estimates, adaptive shrinkage was applied [5]. This leads to shrinkage of log-2 transformed fold changes (log_2(FCs)) towards zero if expression changes are mostly due to noise, whereas relevant log_2(FCs) are preserved. For each comparison, a gene is finally counted as DEG if the effect size fulfils $log_2(FC) > log_2(1.5)$ for upregulation ($log_2(FC) < -log_2(1.5)$ for downregulation) and the estimate is significantly different from zero (i.e. no effect) with a false discovery rate (FDR)-adjusted p-value $p_{adj} < 0.05$. Enrichment analysis for biological processes gene ontology (GO) terms was applied separately for up and down regulated genes for the above-described comparison "Sham + vehicle vs. BDL + vehicle" for the first and last time point and of each area defined in the differentiation pattern (DiPa) plot for all time points. The R package topGO, version 2.56.0, with the elim algorithm was used to focus on more

specific biological process GO groups [6]. P-values of GO groups were FDR-adjusted and considered significant if the adjusted p-value was smaller than 0.05. The DiPa plot was inspired by previous works of [7]. It defines the gene groups unaffected (ua), 1a/1b, 2a/2b and not treatable a (nta) and not treatable b (ntb) by comparing the $\log_2$(FC)s of the comparisons BDL + vehicle vs. Sham + vehicle (x-axis) and BDL + AS0369 vs. Sham + vehicle (y-axis). Using *thres* = $\log_2(1.5)$, the areas are defined as:

- ua: (|x| < thres and |y| < thres) or (|x| < thres and |x-y| < thres)
- 1a: x > thres and |y| < thres and y<x –thres
- 2a: x > thres and y > thres and y<x – thres
- nta: x>thres and y>x-thres.

And accordingly, we define

- 1b: x < -thres and |y|<thres and y>x
- 2b: x < thres and y < -thres and y>x+thres
- ntb: x < thres and y<x+thres
- 3b: |x|<thres and y < -thres.

Genes in 1a that additionally fulfill the stricter $|x|>7\cdot \log_2(1.5)$ are considered extreme genes 1a (accordingly for 1b), as they are extremely affected by the BDL, but rescued by AS0369.

Statistical analysis. Data were analyzed using GraphPad Prism version 10.4.0 Software. The statistical tests used are indicated in the figure legends.

Supplementary Figures

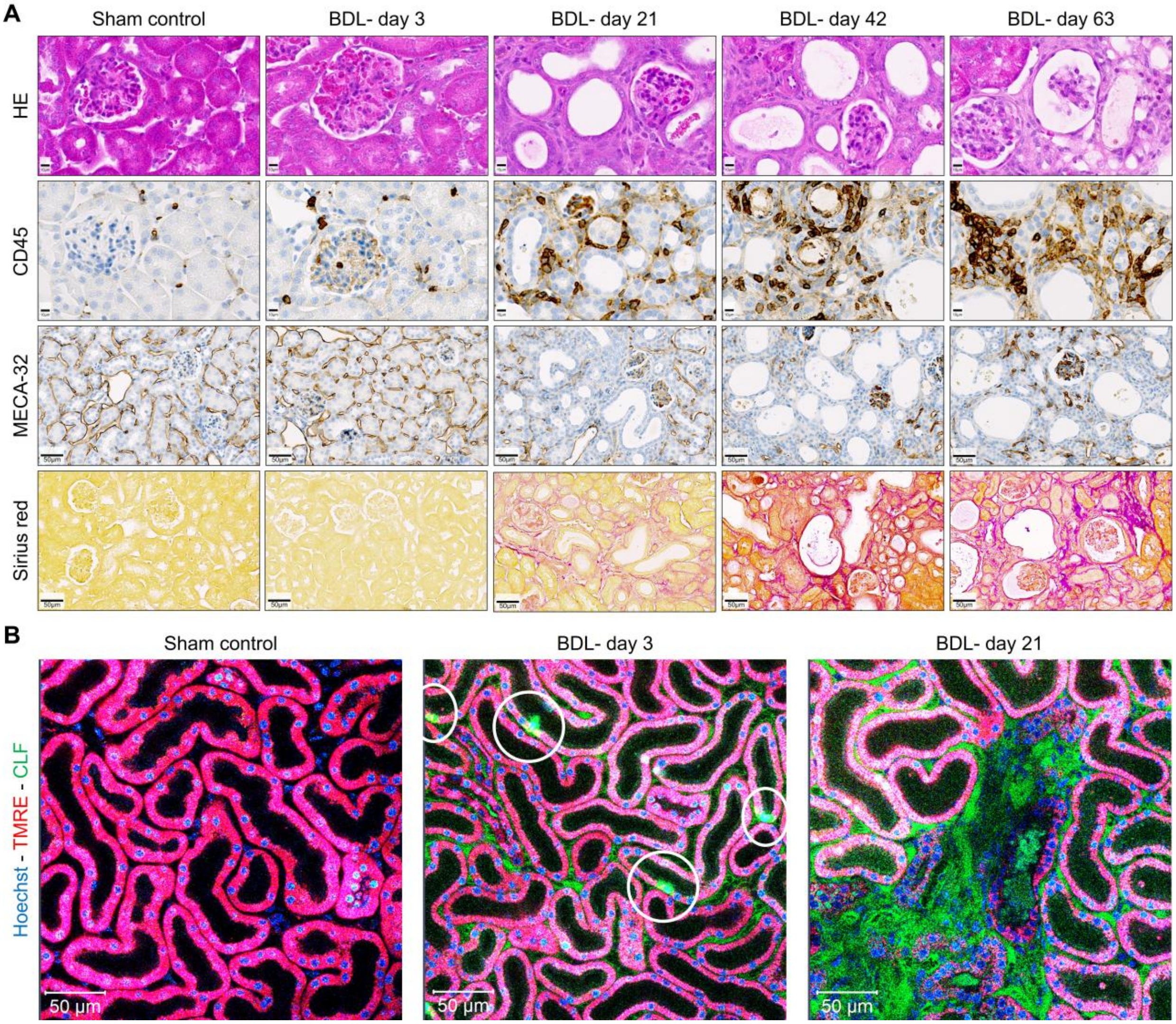

Fig. S1: Renal tissue at days 3, 21, 42 and 63 after BDL to characterize the tissue before the onset of therapy. (A) H&E staining, immunostaining against the leukocyte marker CD45, the endothelial marker MECA-32 and staining of fibrotic structures by Sirius red. (B) Intravital imaging of the kidney. Hoechst dye for visualization of nuclei; tetramethylrhodamine ethyl ester (TMRE) for visualization of mitochondria; Cholyl-lys-fluorescein (CLF): green-fluorescent bile salt analogue; the circles on BDL-day 3 indicate CLF flooded dead cells.

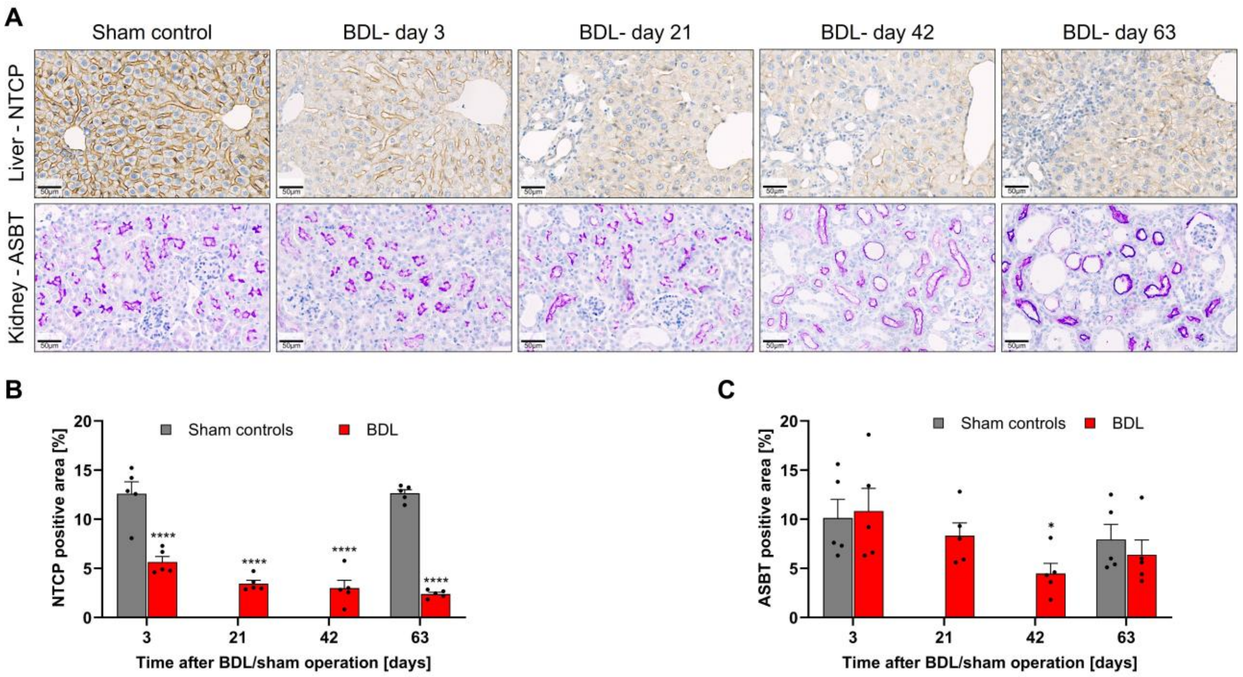

Fig. S2: Expression of the bile acid uptake transporters NTCP in liver and ASBT in kidney tissue. (A) Immunostaining of liver sections against NTCP; (B) Immunostaining of kidney sections against ASBT; (C) Percentage of the NTCP positive area; (D) Percentage of the ASBT positive area. * p<0.05; **** p<0.0001, Šídák's multiple comparisons test.

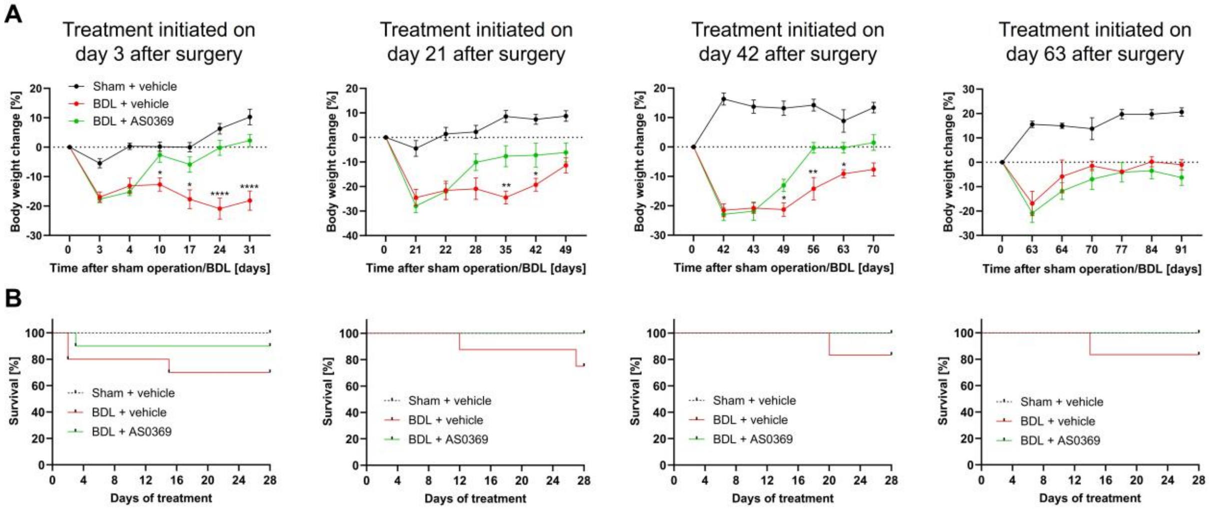

Fig. S3: Influence of AS0369 treatment on body weight and survival. (A) body weight changes; *p <0.05; **p <0.01; ****p <0.0001 compared to the corresponding BDL + AS0369 group; Unpaired t test; (B) Survival during the 28-days treatment period.

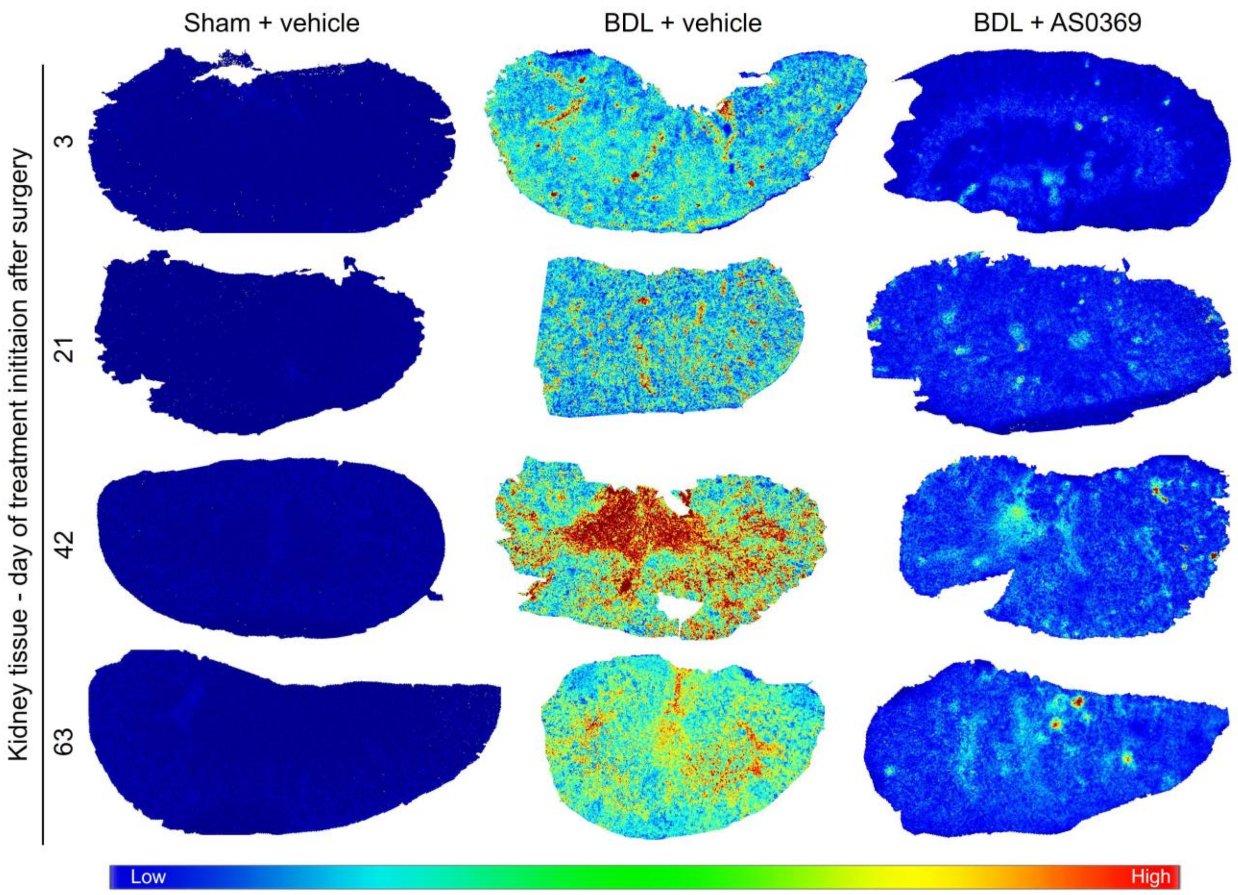

Fig. S4: MALDI-MSI imaging of taurocholic acid (TCA) in renal whole organ sections.

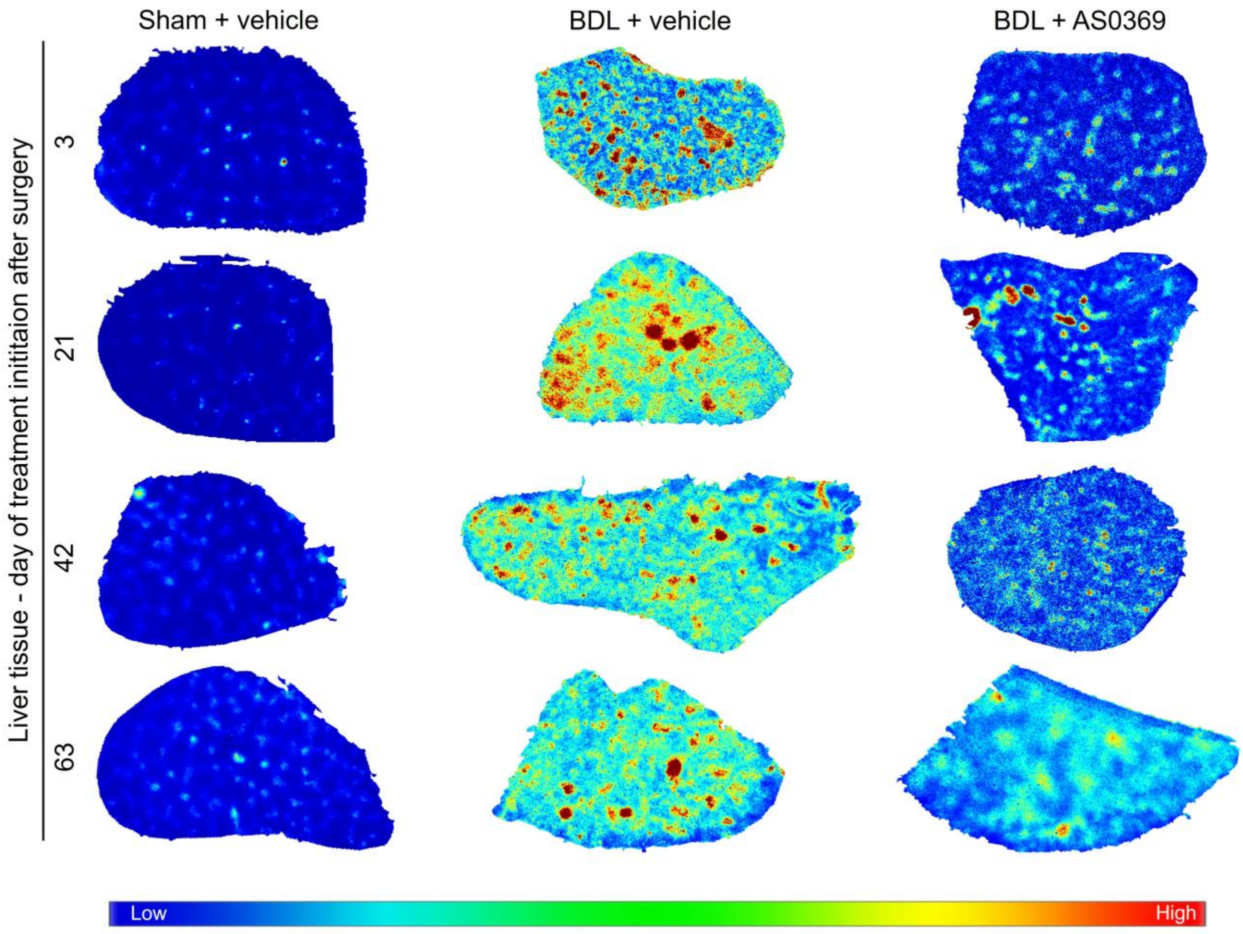

Fig. S5: MALDI-MSI imaging of taurocholic acid (TCA) in whole slide liver tissue sections.

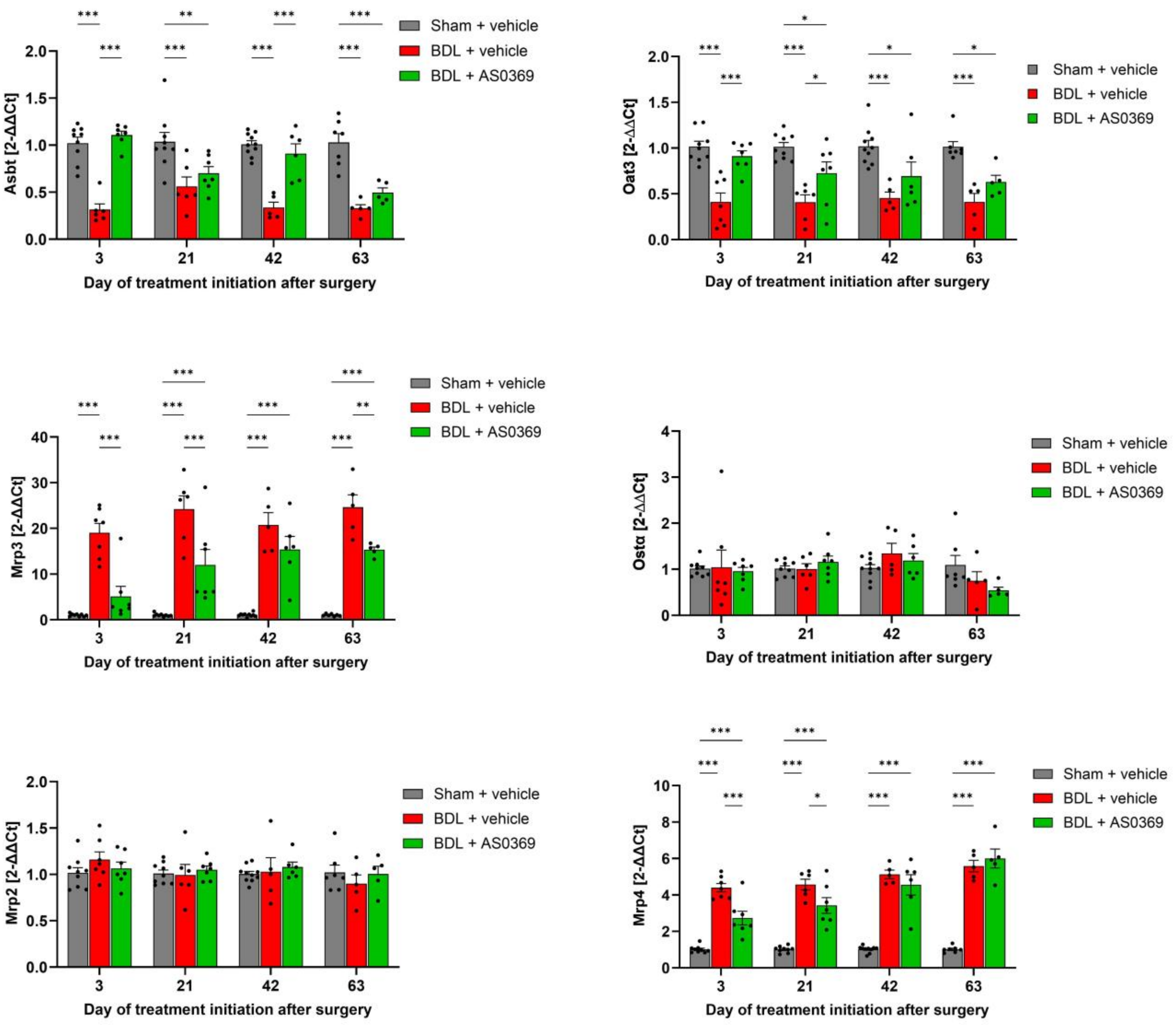

Fig. S6: RNA levels of bile acid transporters in renal tissue homogenate analyzed by qRT-PCR. *p<0.05; **p<0.01; ***p<0.001; Tukey's multiple comparisons test.

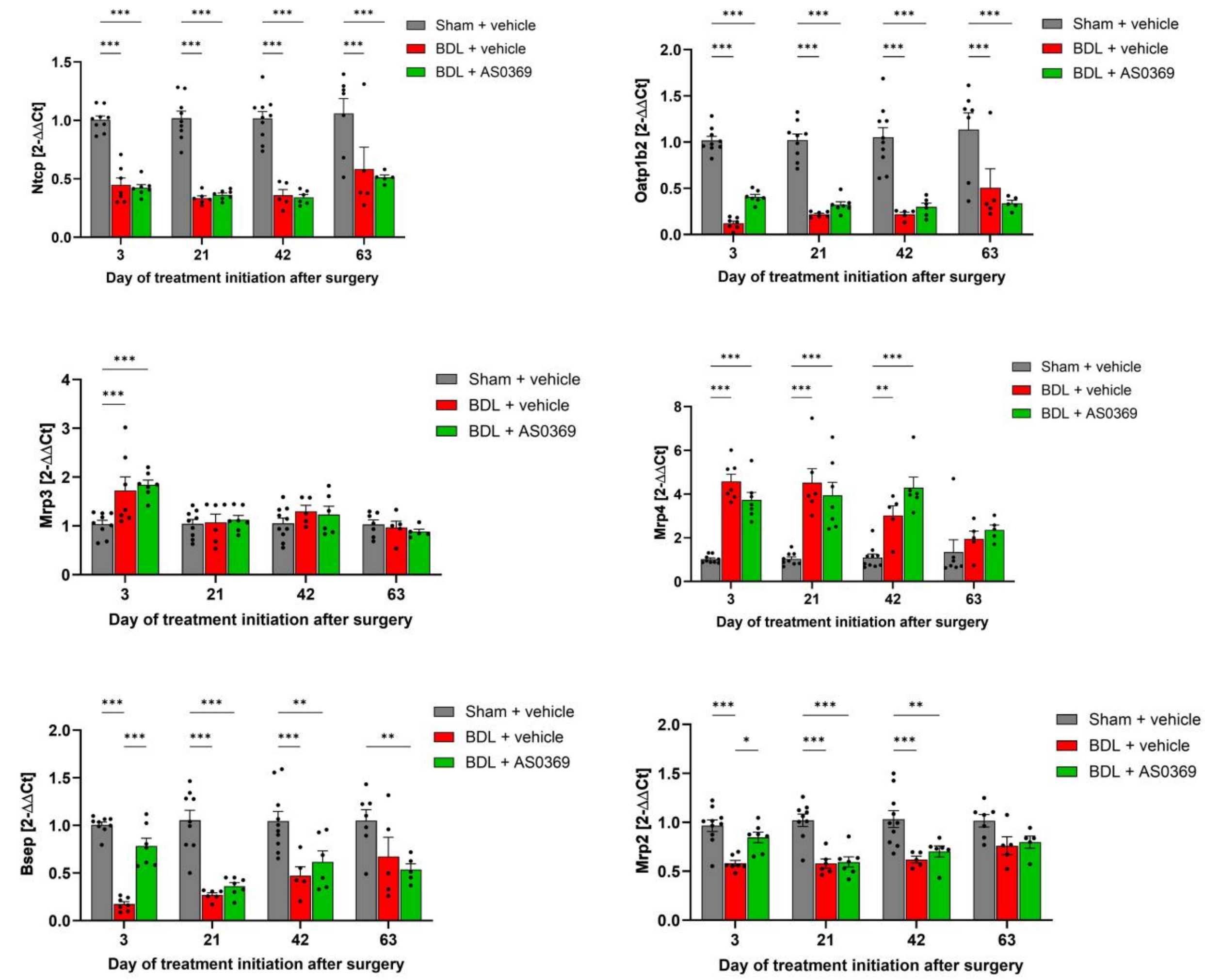

Fig. S7: RNA levels of bile acid transporters in liver tissue homogenate analyzed by qRT-PCR. *p<0.05; **p<0.01; ***p<0.001; Tukey's multiple comparisons test.

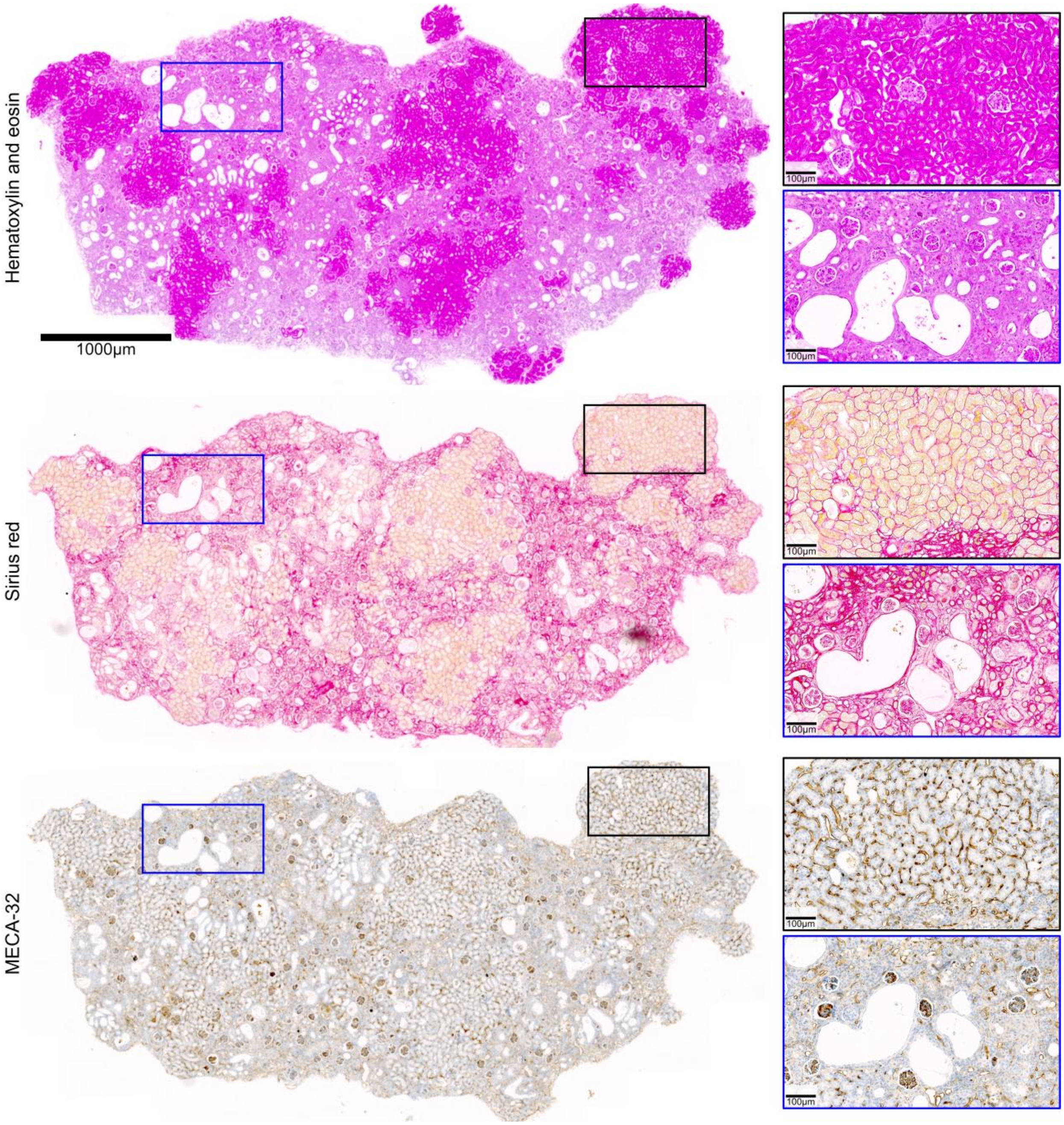

Fig. S8. Hematoxylin and eosin staining of 10-week bile duct-ligated mouse; the pale eosin staining indicates damaged tissue. This fits well with the massive fibrosis (indicated by Sirius red staining) and damage of the peritubular capillaries (indicated by loss of MECA-32 staining).

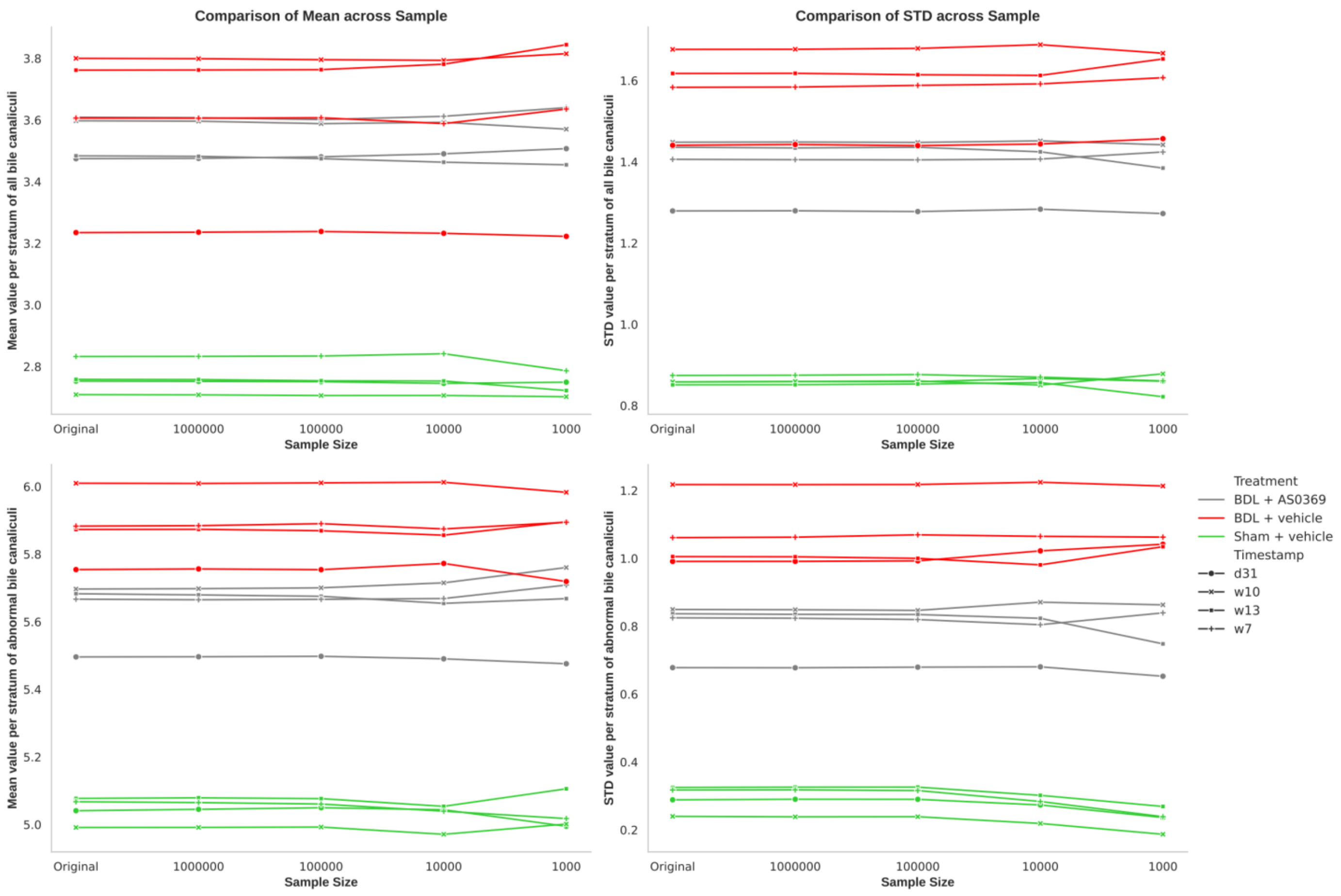

Fig. S9. Consistency of mean and standard deviation under stratified stochastic sampling. The plots show the mean and standard deviation (STD) per experimental group for all bile canaliculi (top row) and abnormal bile canaliculi (bottom row), using decreasing sample sizes per group. Results remain consistent down to 10,000 samples, with only minor changes (maximum changes: mean 0.49%, STD 10.67%), but increase notably at 1,000 samples (maximum changes: mean 2.19%, STD 10.60%).

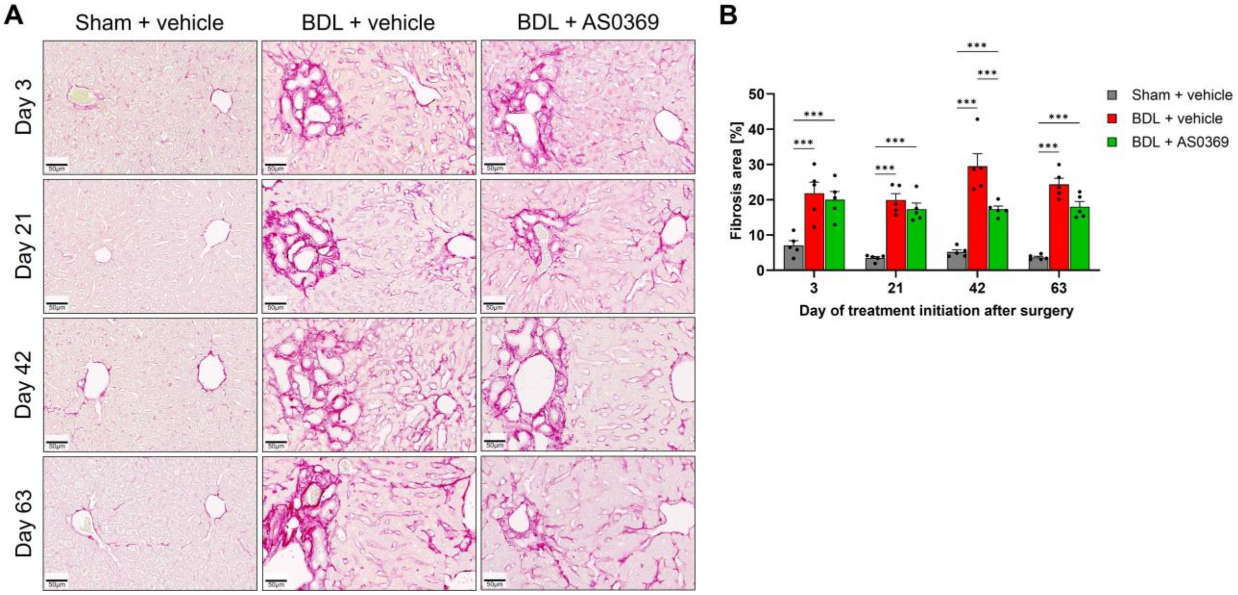

Fig. S10: Fibrosis of liver tissue. (A) Sirius red staining visualizing periportal and perisinusoidal fibrosis. (B) Quantification of the Sirius red positive (fibrotic) area. *** p<0.001, Tukey's multiple comparisons test.

A

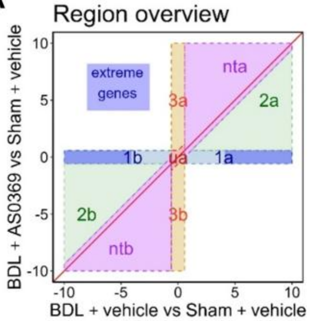

Comparison day 3 to day 63

Starting point \ Migrates to	1a	2a	nta
1a	215	470	1657
2a	1	160	591
nta	11	53	730

Starting point \ Migrates to	1b	2b	ntb
1b	125	383	1441
2b	2	86	158
ntb	3	9	547

Starting point \ Migrates to	1a_extreme	1a_not_extrem	2a	nta
1a_extreme	5	10	52	28

Starting point \ Migrates to	1b_extreme	1b_not_extrem	2b	ntb
1b_extreme	0	2	42	7

B

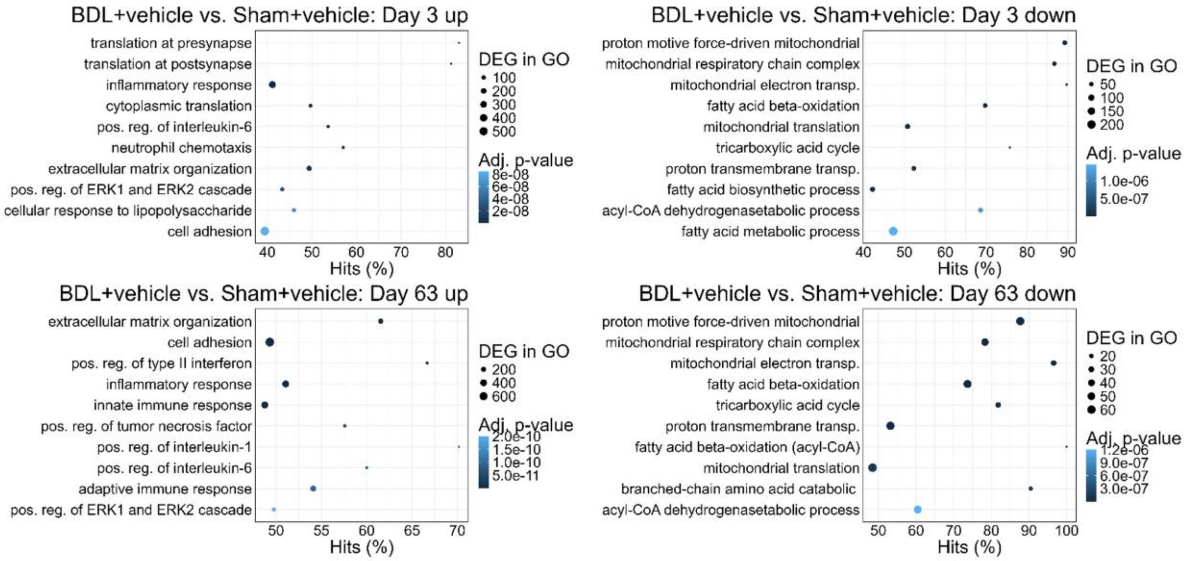

Fig. S11: Gene expression migration and gene ontology (GO) analysis of RNA-seq data of kidney tissue homogenate. (A) DiPa-plot illustrating response to AS0369 therapy. Expression pattern groups 1a and 1b contain genes that are up or down regulated by BDL and are brought back to the normal range by AS0369 therapy. Expression pattern groups 2a and 2b contain genes up or down regulated by BDL that are improved but not completely back to the normal range by AS0369 therapy. nta and ntb represent genes for which AS0369 therapy did not lead to any improvement. The gene expression migration analysis (comparison day 3 to day 63) gives the number of genes in specific expression pattern groups of the day 3 treatment group and informs to which region they migrated in the day 63 treatment group. For example, genes (n=2342) that clustered to region 1a for the day 3 treatment/group clustered to nta (n=1657), 2a (n=470) or remained in 1a for the day 63 treatment group. Expression pattern groups “1a or 1b extreme” contain genes that were up or downregulated by BDL a factor of at least 17 (=1.5^7). A relatively high fraction of genes in the “1a or 1b extreme” expression pattern groups for the day 3 treatment remained in 1a/b or migrated to 2a/b compared to the day 63 treatment group. (B) Gene ontology (GO) analysis of genes up or down regulated in day 3 or day 63 treatment groups.

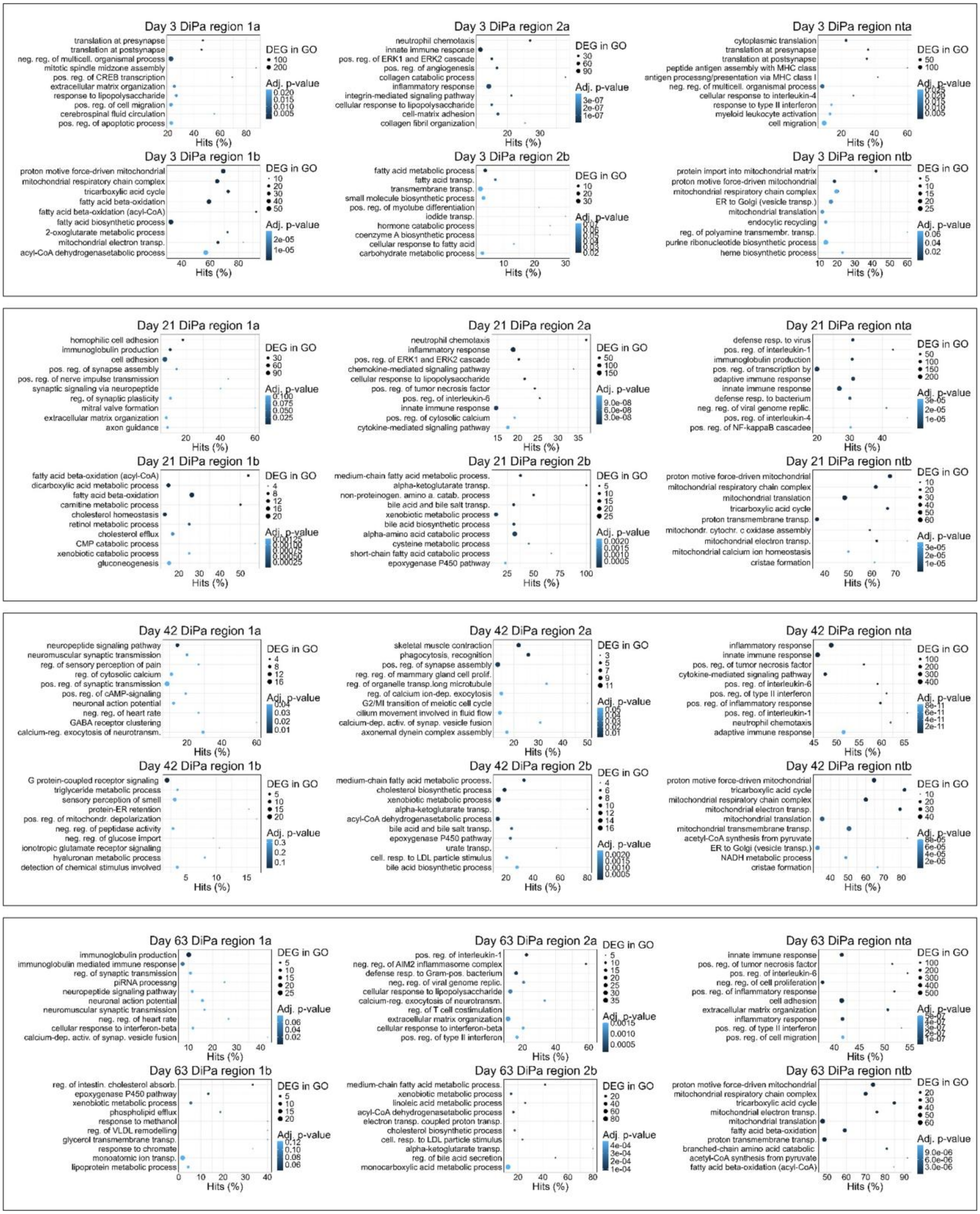

Fig. S12: Gene ontology analysis of genes in the individual DiPa cluster regions for the RNA-seq analysis of kidney tissue homogenate.

A

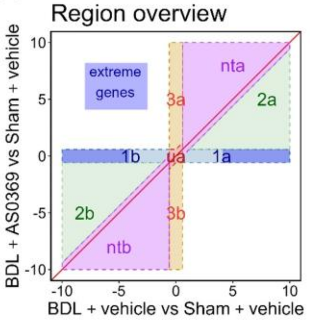

Comparison day 3 to day 63

Starting point \ Migrates to	1a	2a	nta
1a	231	73	209
2a	165	299	200
nta	461	503	882

Starting point \ Migrates to	1b	2b	ntb
1b	55	28	314
2b	21	128	236
ntb	20	22	547

Starting point \ Migrates to	1a_extreme	1a_not_extrem	2a	nta
1a_extreme	3	6	8	1

Starting point \ Migrates to	1b_extreme	1b_not_extrem	2b	ntb
1b_extreme	0	3	2	3

B

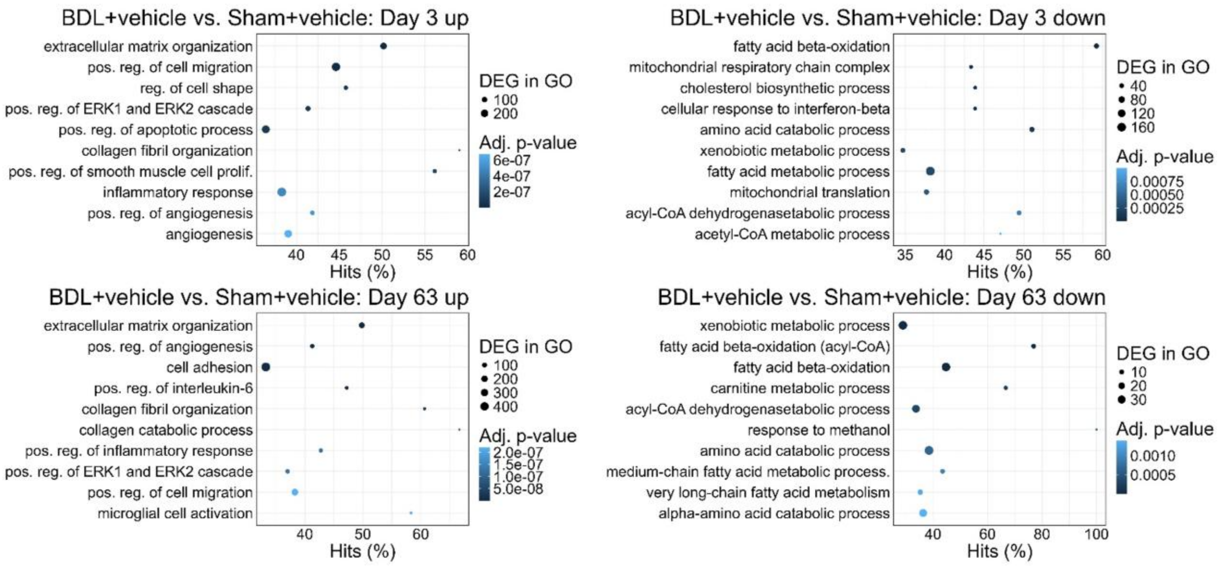

Fig. S13: Gene expression migration and gene ontology analysis of RNA-seq data of liver tissue homogenate. The analysis corresponds to that shown for the kidney data in Fig. S11.

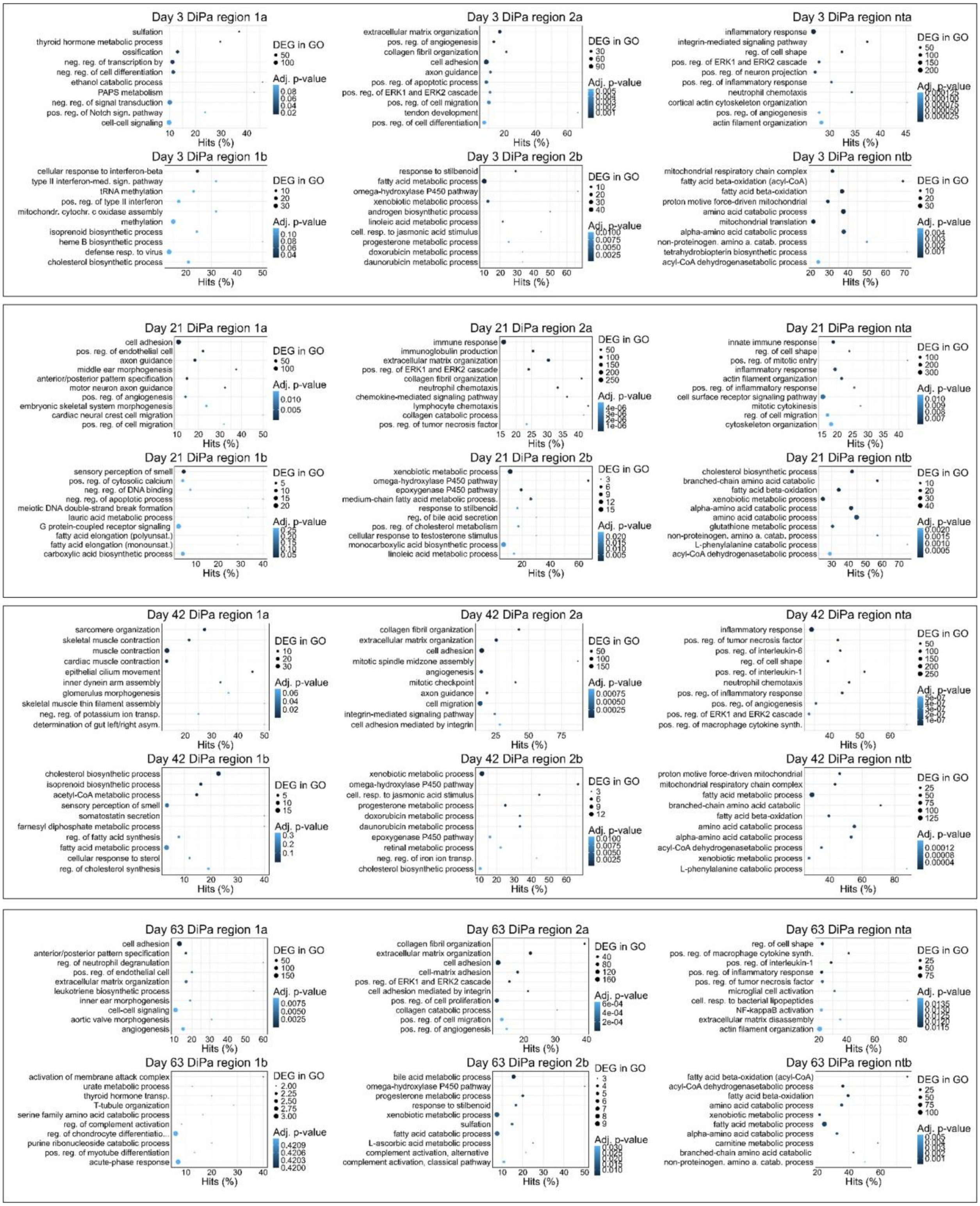

Fig. S14: Gene ontology analysis of genes in the individual DiPa expression pattern groups for the RNA-seq analysis of liver tissue homogenate.

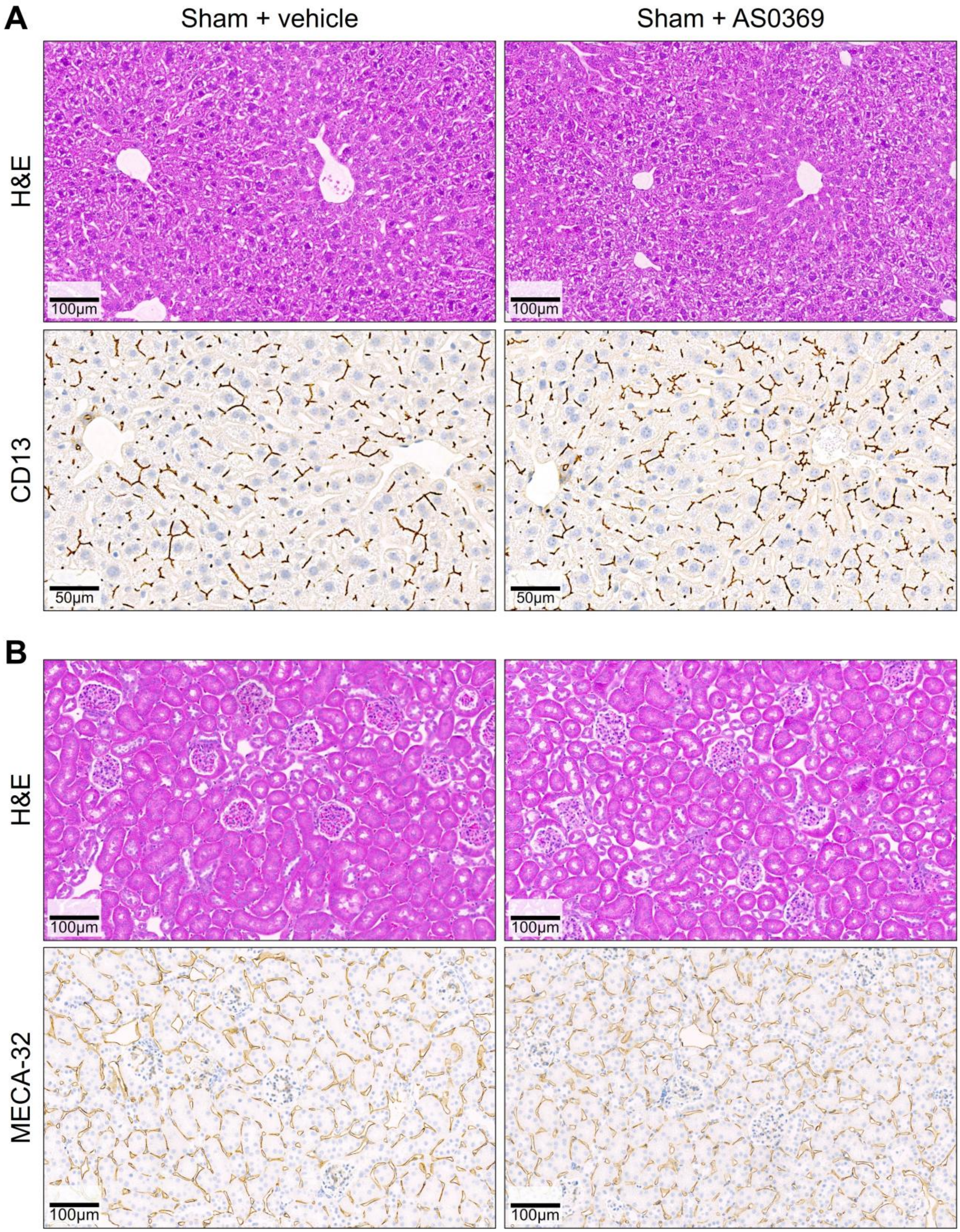

Fig. S15. Systemic ASBT inhibition caused no major histological alteration either in the liver (A) nor in the kidneys (B).

Supplementary Tables

Table S1. Antibodies used for immunohistochemistry.

Target	Primary antibodies		Secondary antibodies	
	Antibody	Dilution	Antibody	Dilution
Cholangiocyte	Anti-cytokeratin 19 antibody, rabbit	1:500	Ultra-Map anti rabbit HRP	Automatic Discovery Ready to use
Bile canaliculi	Recombinant anti-CD13 antibody, rabbit	1:16000	Ultra-Map anti rabbit HRP	
Leukocytes	Anti-CD45 antibody, rat	1:400	Omni-Map anti rat HRP	
Endothelial cells	Anti-MECA-32 antibody, rat	1:250	Ultra-Map anti rat HRP	
ASBT	Anti-ASBT antibody, goat	1:500	Ultra-Map anti goat HRP	
Proliferating cells	Anti-Ki-67 antibody, rabbit	1:100	Ultra-Map anti rabbit HRP	
NTCP	Anti-NTCP antibody, rabbit	1:3000	Ultra-Map anti rabbit HRP	

Table S2. TaqMan gene assays.

Gene	TaqMan Assay ID
Abcb11	Mm00445168_m1
Abcc2	Mm00496899_m1
Abcc3	Mm00551550_m1
Abcc4	Mm01226381_m1
Egr1	Mm00656724_m1
GAPDH	4352932E
Slc1a2	Mm01275814_m1
Slc10a1	Mm00441421_m1
Slc10a2	Mm00488258_m1
Slc51a	Mm00521530_m1
Slc22a8	Mm00459534_m1

Table S3: Bioinformatics of the kidney: Gene movements from day 3 to day 63

starting point | migrates to	1a	2a	3a	nta	ua	anyb	other
**1a**	215	470	14	1657	904	25	0
**2a**	1	160	0	591	4	0	0
**3a**	0	0	2	30	8	3	0
**nta**	11	53	1	730	280	3	0
**ua**	285	183	32	1346	8932	1112	5
**anyb**	34	24	11	106	961	2775	5
**other**	0	1	0	3	5	0	0

starting point | migrates to	1b	2b	3b	ntb	ua	anya	other
**1b**	125	383	4	1441	743	169	0
**2b**	2	86	0	158	2	0	0
**3b**	0	0	2	12	6	0	0
**ntb**	3	9	3	547	210	6	0
**ua**	88	22	41	961	8932	1846	5
**anya**	5	1	4	21	1196	3935	4
**other**	0	0	3	2	5	0	0

Table S4: Bioinformatics of the liver: Gene movements from day 3 to day 63

starting point | migrates to	1a	2a	3a	nta	ua	anyb	other
1a	231	73	4	209	867	29	1
2a	165	299	1	200	74	1	0
3a	57	25	6	120	164	7	0
nta	461	503	4	882	588	19	0
ua	278	27	9	423	9573	522	0
anyb	19	2	14	33	1831	1490	12
other	7	5	8	12	51	0	0

starting point | migrates to	1b	2b	3b	ntb	ua	anya	other
1b	55	28	6	314	885	48	0
2b	21	128	2	236	52	0	0
3b	4	1	22	53	146	13	0
ntb	20	22	31	547	748	7	0
ua	32	2	119	369	9573	737	0
anya	10	0	24	22	1693	3240	32
other	3	0	4	5	51	1	0

Supplementary References

Author names in bold designate shared co-first authorship.

[1] Ghallab A, Gonzalez D, Strangberg E, et al. Inhibition of the renal apical sodium dependent bile acid transporter prevents cholemic nephropathy in mice with obstructive cholestasis. J Hepatol 2024;80:268-281.

[2] Ghallab A, Hofmann U, Sezgin S, et al. Bile Microinfarcts in Cholestasis Are Initiated by Rupture of the Apical Hepatocyte Membrane and Cause Shunting of Bile to Sinusoidal Blood. Hepatology 2019;69:666-683.

[3] Ghallab A, Hassan R, Hofmann U, et al. Interruption of bile acid uptake by hepatocytes after acetaminophen overdose ameliorates hepatotoxicity. J Hepatol 2022;77:71-83.

[4] **Custodio RJP, Hobloss Z, Myllys M**, et al. Cognitive Functions, Neurotransmitter Alterations, and Hippocampal Microstructural Changes in Mice Caused by Feeding on Western Diet. Cells 2023;12.

[5] Holland CH, Ramirez Flores RO, Myllys M, et al. Transcriptomic Cross-Species Analysis of Chronic Liver Disease Reveals Consistent Regulation Between Humans and Mice. Hepatol Commun 2022;6:161-177.

[6] Hall MJ. A staining reaction for bilirubin in sections of tissue. Am J Clin Pathol 1960;34:313-316.

[7] Bankhead P, Loughrey MB, Fernández JA, et al. QuPath: Open source software for digital pathology image analysis. Sci Rep 2017;7:16878.

[8] Guttman A. R-trees: a dynamic index structure for spatial searching. ACM SIGMOD Conference; 1984; 1984.

[9] LeCun Y, Bengio Y, Hinton G. Deep learning. Nature 2015;521:436-444.

[10] Ronneberger O, Fischer P, Brox T. U-Net: Convolutional Networks for Biomedical Image Segmentation. In: Navab N, Hornegger J, Wells WM, Frangi AF, editors. Medical Image Computing and Computer-Assisted Intervention – MICCAI 2015; 2015 2015//; Cham: Springer International Publishing; 2015. p. 234-241.

[11] Isensee F, Jaeger PF, Kohl SAA, et al. nnU-Net: a self-configuring method for deep learning-based biomedical image segmentation. Nat Methods 2021;18:203-211.

[12] Schmidt U, Weigert M, Broaddus C, et al. Cell Detection with Star-Convex Polygons. In: Frangi AF, Schnabel JA, Davatzikos C, Alberola-López C, Fichtinger G, editors. Medical Image Computing and Computer Assisted Intervention – MICCAI 2018; 2018 2018//; Cham: Springer International Publishing; 2018. p. 265-273.

[13] Lee T-C, Kashyap RL, Chu CN. Building Skeleton Models via 3-D Medial Surface/Axis Thinning Algorithms. CVGIP Graph Model Image Process 1994;56:462-478.

[14] Patro R, Duggal G, Love MI, et al. Salmon provides fast and bias-aware quantification of transcript expression. Nature Methods 2017;14:417-419.

[15] Love MI, Soneson C, Hickey PF, et al. Tximeta: Reference sequence checksums for provenance identification in RNA-seq. PLoS Comput Biol 2020;16:e1007664.

[16] Love MI, Huber W, Anders S. Moderated estimation of fold change and dispersion for RNA-seq data with DESeq2. Genome Biology 2014;15:550.
